# Functionalization of 2-Mercapto-5-methyl-1,3,4-thiadiazole: 2-(ω-Haloalkylthio) Thiadiazoles vs. Symmetrical Bis-Thiadiazoles

**DOI:** 10.3390/molecules29091938

**Published:** 2024-04-24

**Authors:** Zhanina S. Petkova, Rusi I. Rusew, Boris L. Shivachev, Vanya B. Kurteva

**Affiliations:** 1 Institute of Organic Chemistry with Centre of Phytochemistry, Bulgarian Academy of Sciences, Acad. G. Bonchev Str., Bl. 9, 1113 Sofia, Bulgaria; zhanina.petkova@orgchm.bas.bg; 2 Institute of Mineralogy and Crystallography “Acad. Ivan Kostov”, Bulgarian Academy of Sciences, Acad. G. Bonchev Str., Bl. 107, 1113 Sofia, Bulgaria; r.rusev93@gmail.com

**Keywords:** 2-mercapto-5-methyl-1,3,4-thiadiazole, ω-haloalkylthio derivatives, bis-thiadiazole ligands, NMR, XRD

## Abstract

A study on the functionalisation of 2-mercapto-5-methyl-1,3,4-thiadiazole has been conducted, yielding two series of products: 2-(ω-haloalkylthio)thiadiazoles and symmetrical bis-thiadiazoles, with variable chain lengths. The experimental conditions were optimised for each class of compounds by altering the base used and the reagents’ proportions, leading to the development of separate protocols tailored to their specific reactivity and purification needs. The target halogenide reagents and bis-thiadiazole ligands were obtained either as single products or as mixtures easily separable by chromatography. Characterisation of the products was performed using 1D and 2D NMR spectra in solution, complemented by single crystal X-ray diffraction (XRD) for selected samples, to elucidate their structural properties.

## 1. Introduction

**Figure 1:**
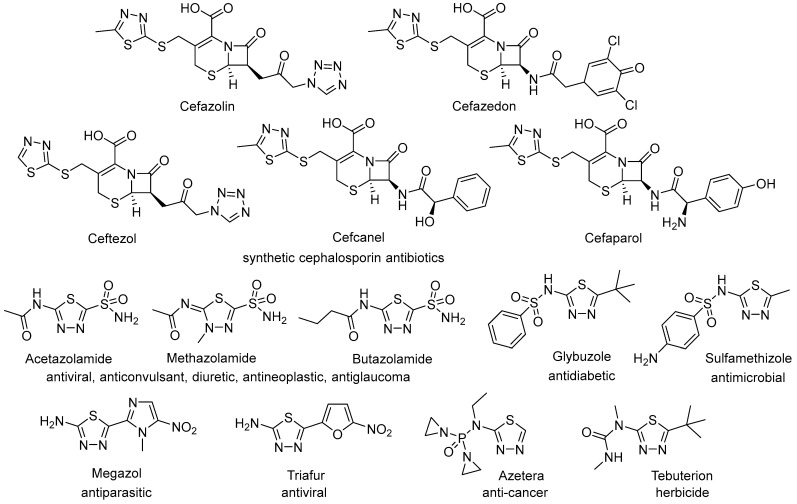
Products available on the market containing a thiadiazole unit.

The 1,3,4-thiadiazole motif serves as a structural subunit in numerous products with remarkable biological activities [1,2,3,4,5,6,7,8,9], such as anticancer [10,11,12,13,14,15], antimicrobial [16,17,18], antiepileptic [19,20,21], among others. Many drugs featuring the 1,3,4-thiadiazole unit are commercially available and have become widely prescribed medications. Notable examples include a series of synthetic and semi-synthetic cephalosporin antibiotics, sulfonamide drugs with multifunctional activities, among a few others, illustrated in Figure 1. In addition to their diverse biological activities, derivatives of 1,3,4-thiadiazole have also been employed as ligands for metal complexes [22,23], in chelating resins [24], as components of azo dyes [25], and as lubricant additives [26], demonstrating their versatility across various applications.

Several synthetic protocols for the preparation of 1,3,4-thiadiazole-containing bioactive compounds have been developed and summarised [2,8,17,27,28,29,30,31,32,33], the main part based on ring-closure reactions. Variable procedures are applied, like intramolecular cyclization of thiosemicarbazides [15,25] or potassium salt of hydrazinodithio formate [34] in concentrated sulphuric acid, condensation of thiosemicarbazide with carboxylic acids [35], benzoic acids [36,37], or N-arylsulfonylated amino acids [38], thiocarbohydrazides with aldehydes [39], hydrazinecarbodithioates or thiosemicarbazones with hydrazonoyl chlorides [40], sulfonamide diazonium chlorides with phenacyl thiocyanate [41], etc. At the same time, methods using an already built 1,3,4-thiadiazole unit are also exploited. The sulfonamides acetazolamide [42,43] and methazolamide [44] and similar bioactive products [45,46,47] are obtained by functionalisation of 5-amino-2-mercapto-1,3,4-thiadiazole, while the synthetic protocols for the preparation of cephalosporin antibiotics cefazolin [48,49], cefazedon [50,51], cefcanel [52], and cefaparol [53] involve 2-mercapto-5-methyl-1,3,4-thiadiazole. To the best of our knowledge, the synthesis of ω-haloalkylthio derivatives, which can be used as alkylating agents to obtain libraries of products, is not reported in the literature.

Sulphur-containing ligands, on the other hand, have shown remarkable coordination properties [54,55,56,57,58,59,60,61] and have displayed a wide range of applications [62,63,64,65,66,67,68]. Among them, heterocyclic thioethers have exhibited a broad spectrum of bioactivities [69,70,71,72] and efficiency as adsorbents for lead ion removal from water [73], ligands in N-heterocyclic carbene (NHC) complexes [74,75,76,77], metal–organic frame-works [78,79,80,81,82], etc. As a very particular example, it is shown that symmetrical bis-1,3,4-thiadiazole-containing bis-thioethers tune the framework formation of silver(I) coordination architectures [83]. Ligands with methylene and ethylene linkers are obtained and it is found that the prolongation of the hydrocarbon bridge results in increased flexibility and diversity in coordination modes.

Herein, we present a study on the functionalisation of 2-mercapto-5-methyl-1,3,4-thiadiazole with α,ω-dihalogenides with variable chain lengths, leading to 2-(ω-haloalkylthio) thiadiazoles and/or symmetrical bis-thiadiazoles, with an emphasis on the optimisation of conditions for the synthesis of both alkylation agents (bromides and chlorides) and bis-heterocyclic ligands.

## 2. Results and Discussion

**Scheme 1:**
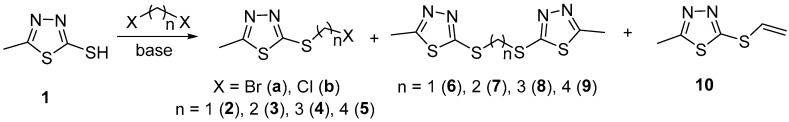
Synthesis of the alkylating agents **2**–**5** and bis-thiadiazole ligands **6**–**9**.

The functionalisation of 2-mercapto-5-methyl-1,3,4-thiadiazole (**1**) with α,ω-dihalogenoalkanes was performed by applying the one-step procedure shown in Scheme 1. The efforts were initially directed towards optimisation of the protocols for the synthesis of halogenide reagents. To obtain the methylene-bridged compound **2**, the starting thiadiazole (**1**) was first reacted with dibromomethane (DBM). Solvent, base, reagents’ proportions, and temperature were varied and it was observed that the bis-thiadiazole **6** was the only product in all cases, while not even traces of bromide **2a** were detected. Selected examples are presented in Table 1, entries 1–3. As seen, notwithstanding the distinction in the reaction conditions, ligand **6** was isolated in commensurable good yields; 74–76%. In an attempt to override the bis-alkylation and to obtain the corresponding alkylating agent, chloride (**2b**), the transformation was performed in dichloromethane used both as reagent and solvent, i.e., in vast excess. Since it is well documented that dichloromethane can act as a double alkylating agent towards amines in the presence of sodium hydroxide as a base [83], weaker organic bases were applied in the experiments. Independently of the latter, compound **6** was again the predominant product, but together with chloride **2b**, which was isolated in up to 25% yield (entries 4 and 5).

Further attempts were focused on the preparation of ethylene-bridged compounds. The reaction was carried out with two equiv. of dibromoethane (DBE) in order to facilitate the mono-substitution process. Variable results were obtained depending on the base used. When using potassium carbonate in acetonitrile at room temperature (entry 6) the bromide **3a** was isolated in only 13% yield in parallel with 30% of bis-thiadiazole **7** and 12% of a side-product, olefin **10**. The latter was obtained quantitatively by using potassium hydroxide in refluxing ethanol (98% yield), the protocol reported in the literature [23] for the preparation of **7**. This fact is an indication that the heating has to be avoided at least when using an excess of the reagent, which hinders the bis-alkylation process. Contrary, when we performed the reaction with triethylamine in ether, both products were isolated in high common yield (entry 7); the bromide **3a** being predominant. The reaction with dichloroethane (DCE), used both as reagent and solvent, led to a quantitative yield of the desired chloride **3b** (entry 10), as already reported by us [84].

The reactions with two equiv. of dibromopropane (DBP) and dibromobutane (DBB) were carried out in ether at room temperature and with triethylamine as a base. In both cases, the desired products **4a** (entry 11) and **5a** (entry 15) were obtained in good yields along with the corresponding bis-thiadiazoles **8** and **9** as mixtures easily separable by chromatography. No olefin formation was detected under these particular conditions. Dichloropropane (DCP) and dichlorobutane (DCB), used both as reagents and solvents, in the presence of triethylamine as a base led, like in the case of **3b**, to the corresponding chlorides **4b** (entry 14) and **5b** (entry 18) as sole products with 93% and 98% yield, respectively.

It should be noted that both bromides, **3a**–**5a**, and chlorides, **3b**–**5b**, are not stable enough and decompose after prolonged storage at room temperature; the process being faster in bromides.

Further attempts were focused on the optimisation of the protocols for the synthesis of bis-thiadiazole ligands **7**–**9**. In order to tune the reaction output towards predominant formation of disubstituted products, the experiments were performed by alteration of the thiadiazole/reagent proportions, namely with close to a 2:1 ratio instead of 1:2. For all three ligands, the 2:1.1 ratio was found to be the most effective and the bis-thiadiazoles **7** (entry 9), **8** (entry 13), and **9** (entry 17) were obtained in 69%, 73%, and 78% yield, respectively. Thus, it can be summarised that only methylene-bridged thiadiazole is obtained as a sole product, while the corresponding bromides are also formed with the lengthening of the hydrocarbon bridge.

These results show that the efficiency of the synthetic protocols for both alkylating agents and symmetrical ligands is strongly dependent on the length of the hydrocarbon bridge and the type of the halogen atom. It is clear that the transformations with dihalogenomethanes lead to complete or predominant bis-substitution, while with the prolongation of the linker length the reaction output follows a common pattern. Bromides are always obtained as mixtures with the corresponding bis-thiadiazole, except for the methylene-linked compound **2a**, where not even traces of the desired product are detected. Chlorides are formed, by using a vast excess of the reagent as a minor component, from dichloromethane (**2b**) or as sole products from the rest of dichlorides (**3b**–**5b**) in excellent yields. Bis-thiadiazole ligands are generated as the only products when using dibromomethane as a reagent (**6**) independently of the conditions, and as major components with the remaining bromides when using an excess of the starting mercaptothiadiazole.

**Figure 2:**
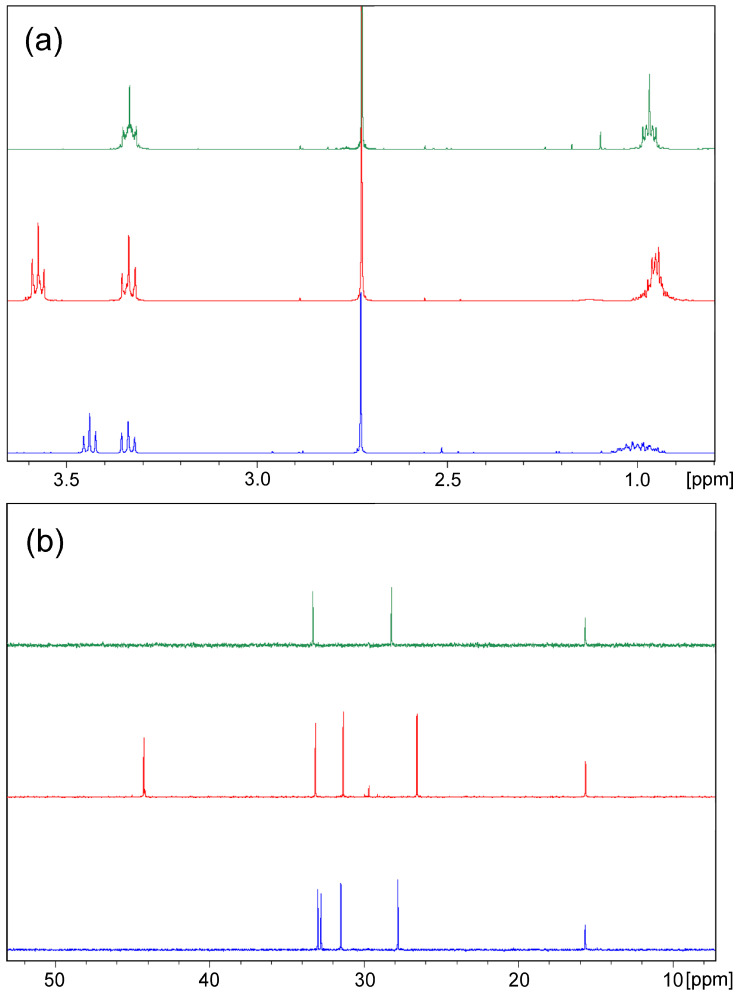
Aliphatic area of ^1^H (**a**) and ^13^C (**b**) NMR spectra of compounds **5a** (blue), **5b** (red), and **9** (green).

The structures of the products are assigned by 1D and 2D NMR spectra and confirmed by single crystal XRD of selected samples. The proton NMR spectra of the products with identical bridge length follow a characteristic pattern (Table S1); very similar to the chemical shifts of the signals for the methylene groups connected with sulphur but with double integral intensity in the spectrum of bis-thiadiazole compared to that of the corresponding halogenide. The main difference in carbon spectra is reasonably in the chemical shift of methylene group signals due to the influence of the halogen atom. The signals for the methylene groups directly connected to halogen are shifted up-field in bromides and down-field in chlorides, while those for methylene groups neighbouring to sulphur possess commensurable shifts, excluding methylene-bridged chloride **2b**. The observed pattern is illustrated by the example of the butylene-linked compounds **5a**, **5b**, and **9** in Figure 2.

**Figure 3:**
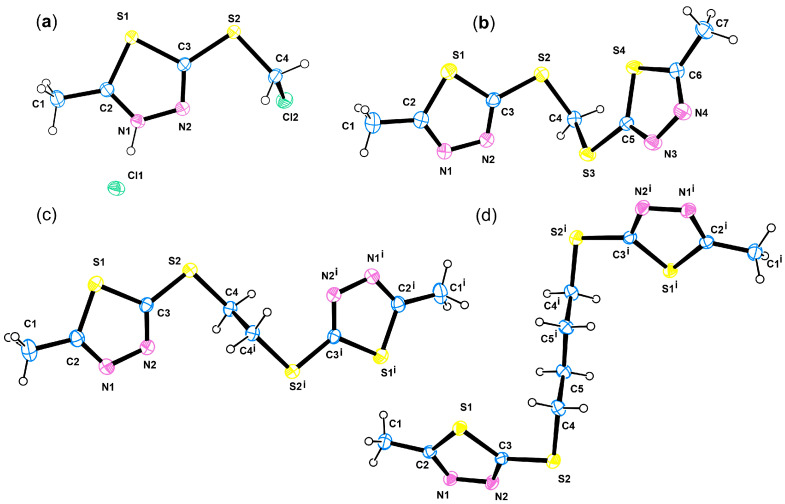
ORTEP views of **2b** hydrochloride (**a**), **6** (**b**), **7** (**c**), and **9** (**d**).

Single crystals for X-ray diffraction (XRD) analysis were grown through slow evaporation from unsaturated solutions of each compound; the colourless oil **2b** being crystallised as hydrochloride. ORTEP views, depicting the molecules present in the asymmetric unit of the crystal structure, are provided in Figure 3, while essential data collection and crystallographic refinement parameters are presented in Table S2.

**Figure 4:**
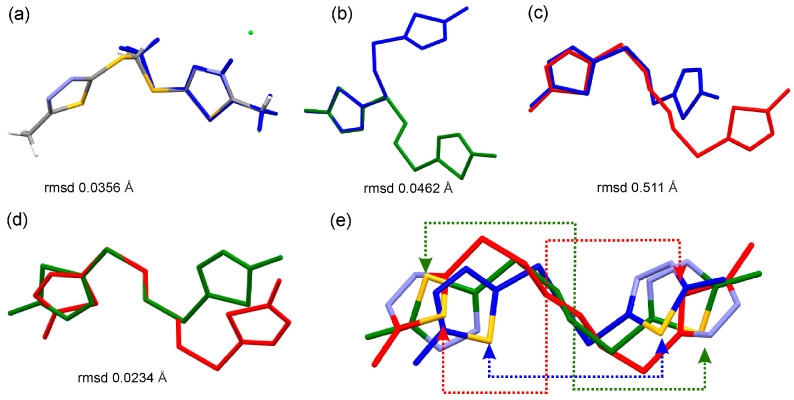
Overlay of (**a**) the molecules of **2b** hydrochloride (grey) and **6** (blue) based on the 1,3,4-thiadiazole moiety; (**b**) compounds **6** (blue) and **7** (green), (**c**) **6** (blue) and **9** (red), (**d**) **7** (green) and **9** (red) disclosing the conserved geometry of 1,3,4-thiadiazole moiety and a variable molecular structure due to the variable C–C chain lengths; and (**e**) overlay of **6**, **7**, and **9**, the dashed lines highlight the different rotations of the thiadiazole around the C–S bond, the arrows point at S atoms.

Compound **2b** has one 2-mercapto-5-methyl-1,3,4-thiadiazole moiety while compounds **6**, **7**, and **9** comprise two such moieties bridged by methyl, ethyl, and butyl linkers. The crystal structures of **2b**, **6**, **7**, and **9** disclosed a conserved geometry of the active 1,3,4-thiadiazole motif (Figure 4a). Indeed, the values of the bond distances and angles for the 1,3,4-thiadiazole are comparable to those of similar compounds [85,86,87].

In **2b**, with the exception of the Cl2 all other atoms lie basically on the mean plane of the 1,3,4-thiadiazole moiety (Figure S1a) disclosing a distinct electronic conjugation. In **6**, the angle between the mean planes of the 1,3,4-thiadiazole moieties is 65.9° (Figure S2). Further analysis of the molecular structure of **6** reveals that the rotation of the 1,3,4-thiadiazole ring along the S2–C3 and S3–C5 bond is permitted, resulting in two distinct arrangements of the thiadiazole rings around the S2/C4/S3 linker plane. The first arrangement of the 1,3,4-thiadiazole in **6** features a N2 atom pointing to the linker S2/C4/S3, thus it is closer to the linker plane. The second arrangement of the 1,3,4-thiadiazole in **6** the S4 is closer to the S2/C4/S3 plane (Figure S1b), e.g., it is pointing toward the linker. In compounds **7** and **9**, the asymmetric unit features half of the molecule, the second half is generated through symmetry operation. The result is that the rotation of the 1,3,4-thiadiazole moiety along the S–C bond is not permitted for the specific crystal structure of **7** and for the specific crystal structure of **9**. This man-made, introduced restriction has a limited individual crystallographic validity, as the comparison of the molecules of **7** and **9** demonstrates that 1,3,4-thiadiazole moieties have adopted different orientations. In compound **7**, the N2 atom is closer to the 2(CH_2_) spacer, while in **9**, the S atom is closer to the 4(CH_2_) spacer linking the two thiadiazole moieties (Figure 4e and Figure S1c,d).

It is noteworthy that the melting points of bis-thiadiazoles deviate from the anticipated trend, which suggests a decrease with the elongation of the (CH_2_)_n_ bridge. Contrary to expectations, the melting points demonstrate an ascending sequence as follows: propylene (compound **8**; oily solid) has the lowest melting point, followed by methylene (compound **6**; 88.6–89.0 °C) and butylene (compound **9**; 93.4–93.9 °C), and ethylene (compound **7**; 132.9–133.6 °C) has the highest. This observation indicates that the melting points of these compounds are significantly influenced by their spatial orientation, allowing for their categorisation into two distinct groups based on the number of methylene units in the linker: compounds with an odd number (**6** and **8**) and those with an even number (**7** and **9**) of methylene groups. This differentiation suggests a clear relationship between spatial configuration and melting behaviour.

**Figure 5:**
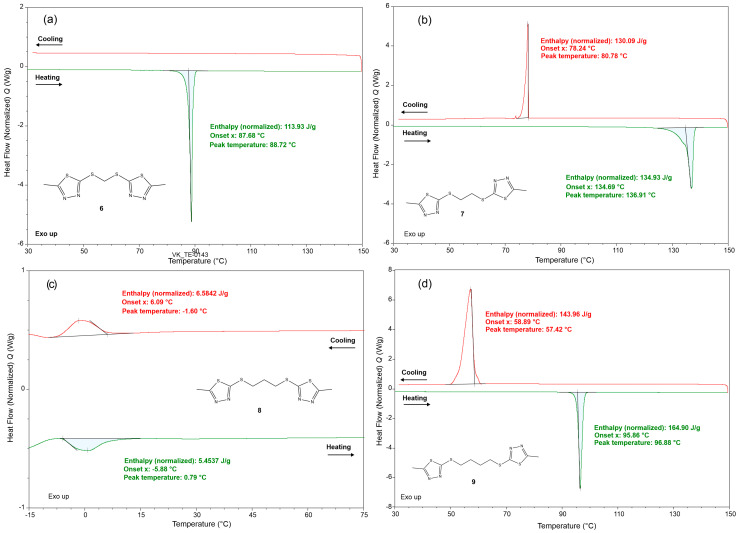
DSC thermograms of (**a**) compound **6**, (**b**) compound **7**, (**c**) compound **8**, and (**d**) compound **9**; the heating and cooling curves are given in green and red, respectively.

The DSC thermograms of compounds **6**, **7**, **8**, and **9** are presented in Figure 5.

The heating and cooling curves are given in green and red, respectively. The thermograms of compounds **6**, **7**, and **9** (Figure 5a,b,d) reveal that their crystal structures are conserved up to the melting points expressed with sharp and intensive endothermic peaks with maxima at 89 °C (H = 113.93 J/g), 137 °C (H = 134.93 J/g), and 97 °C (H = 164.90 J/g), respectively. No other thermal effects, such as phase transitions and decomposition, are observed for **6**, **7**, and **9** upon heating to 150 °C. The cooling of **6**, **7**, and **9** (from 150 to 30 °C) discloses exothermic effects only for **7** and **9** that are related to recrystallisation of the melt. This is confirmed by the similar values for the crystallisation and melting peak areas but also by the fact that the degree of supercooling (the difference between the onset temperatures of melting and crystallisation) does not exceed 50 K. For **6**, the recrystallisation is not registered within the used temperature range. At ambient conditions, compound **8** appears as a viscous oil. It was observed that it solidifies when stored in a refrigerator (6–8 °C). Thus, the DSC heating and cooling range for compound **8** was adjusted to the range –15 to 75 °C. The thermogram for **8** reveals that the liquid-to-solid transition is represented by a broad and shallow exothermic effect with a maximum at −1.6 °C starting from 6 °C. The heating from −15 to 75 °C of **8** produces only one endothermic effect, onset at −5.88 °C and maximum at 0.79 °C.

## 3. Materials and Methods

### 3.1. General

All reagents were purchased from Aldrich (St. Louis, MO, USA), Merck (Rahway, NJ, USA), and Fluka (Buchs, Switzerland) and were used without any further purification. The deuterated chloroform was purchased from Deutero GmbH (Kastellaun, Germany). Fluka silica gel (TLC-cards 60778 with fluorescent indicator 254 nm) was used for TLC chromatography and R_f_-value determination. Merck Silica gel 60 (0.040–0.063 mm) was used for flash chromatography purification of the products. The melting points were determined in capillary tubes on an SRS MPA100 OptiMelt (Sunnyvale, CA, USA) automated melting point system with heating rate of 1 °C per min. The NMR spectra were recorded on Bruker Avance II+ 600 or Bruker Avance NEO 400 spectrometers (Rheinstetten, Germany) in CDCl_3_; the chemical shifts are quoted in ppm in δ-values against tetramethylsilane (TMS) as an internal standard and the coupling constants were calculated in Hz. The assignment of the signals is confirmed by applying two-dimensional HSQC and HMBC techniques. The spectra were processed with the Topspin 3.6.3 program. The IR spectra were measured on a Shimadzu IR Spirit FT-IR spectrometer (Shimadzu Corporation, Columbia, SC, USA) using QATR-S as a single-reflection ATR measurement attachment. The mass spectra were recorded using a Q Exactive Plus Hybrid Quadrupole-Orbitrap Mass Spectrometer, Thermo Scientific (HESI HRMS) in positive mode. The spectra were processed by the Thermo Scientific FreeStyle program version 1.8 SP1 (Thermo Fisher Scientific Inc., Waltham, MA, USA). DSC experiments were performed on Discovery DSC 250 (TA Instruments, New Castle, DE, USA). Samples between 2 and 5 mg were heated in closed aluminum pans from 30 to 150 °C and then cooled back to 30 °C for compounds **6**, **7**, and **9**, while for **8** the range was −15 to 75 °C. In all cases, the heating and cooling rate was 4 °C·min^−1^ under nitrogen flow of 30 mL·min^−1^.

### 3.2. Reaction with α,ω-Dibromoalkanes

To a solution of 2-mercapto-5-methyl-1,3,4-thiadiazole (4 mmol) and base (8 mmol) in variable solvent (20–40 mL), α,ω-dibromoalkane (2–8 mmol) was added and the mixture was stirred at rt or at reflux for different time (cf. Table 1). The solid residue (if formed) was filtered off and the solvent was removed in vacuo. The crude mixture was purified by flash chromatography on a silica gel with a gradient of polarity from DCM to 5% acetone in DCM.

*Bromide* **3a**: R_f_ 0.46 (1% acetone in DCM); colourless solid; m. p. 34.1–34.4 °C; ^1^H NMR 2.738 (s, 3H, C*H*_3_), 3.724 (m, 2H, C*H*_2_-S), 3.743 (m, 2H, C*H*_2_-Br); ^13^C NMR 15.66 (*C*H_3_), 29.69 (*C*H_2_-Br), 35.18 (*C*H_2_-S), 163.69 (*C*_q_-2), 165.50 (*C*_q_-5); IR (ATR) 1433, 1413, 1381, 1200, 1178, 1078, 1057, 618, 601 cm^−1^; HR-MS (HESI^+^) *m*/*z* calcd. for C_5_H_7_BrN_2_S_2_^+^ [M + H]^+^ 238.9307, found 238.9308, ∆ = 0.1 mDa.

*Bromide* **4a**: R_f_ 0.30 (1% acetone in DCM); colourless oil; ^1^H NMR 2.372 (m, 2H, C-C*H*_2_-C), 2.729 (s, 3H, C*H*_3_), 3.451 (t, 2H, J 6.8, C*H*_2_-S), 3.551 (t, 2H, J 6.3, C*H*_2_-Br); ^13^C NMR 15.65 (*C*H_3_), 31.66 (*C*H_2_-Br), 31.72 (C-*C*H_2_-C), 32.09 (*C*H_2_-S), 164.79 (*C*_q_-2), 165.12 (*C*_q_-5); IR (ATR) 1499, 1205, 1184, 1050, 986, 655, 505 cm^−1^; HR-MS (HESI^+^) *m*/*z* calcd. for C_6_H_9_BrN_2_S_2_^+^ [M + H]^+^ 252.9463, found 252.9460, ∆ = −0.3 mDa.

*Bromide* **5a**: R_f_ 0.32 (1% acetone in DCM); colourless oil; ^1^H NMR 1.970 (m, 2H, C*H*_2_-C-S), 2.032 (m, 2H, C*H*_2_-C-Br), 2.726 (s, 3H, C*H*_3_), 3.337 (t, 2H, J 7.0, C*H*_2_-S), 3.438 (t, 2H, J 6.4, C*H*_2_-Br); ^13^C NMR 15.64 (*C*H_3_), 27.78 (*C*H_2_-C-S), 31.47 (*C*H_2_-C-Br), 32.76 (*C*H_2_-Br), 32.97 (*C*H_2_-S), 164.94 (*C*_q_-5), 165.22 (*C*_q_-2); IR (ATR) 1452, 1428, 1382, 1308, 1187, 1085, 732, 646, 606 cm^−1^; HR-MS (HESI^+^) *m*/*z* calcd. for C_7_H_11_BrN_2_S_2_^+^ [M + H]^+^ 266.9620, found 266.9618, ∆ = −0.2 mDa.

*Bis-thiadiazole* **6**: R_f_ 0.51 (5% acetone in DCM); colourless solid; m. p. 88.6–89.0 °C (lit. [23] 78–80 °C); ^1^H NMR 2.758 (s, 6H, C*H*_3_), 5.197 (s, 2H, C*H*_2_); ^13^C NMR 15.76 (*C*H_3_), 36.88 (*C*H_2_), 163.60 (*C*_q_-2), 165.91 (*C*_q_-5); IR (ATR) 1379, 1187, 1074, 1035, 814, 717, 669, 612 cm^−1^.

*Bis-thiadiazole* **7**: R_f_ 0.43 (5% acetone in DCM); colourless solid; m. p. 132.9–133.6 °C (lit. [23] 135–136 °C); ^1^H NMR 2.734 (s, 6H, C*H*_3_), 3.759 (s, 4H, C*H*_2_); ^13^C NMR 15.68 (*C*H_3_), 33.00 (*C*H_2_), 164.45 (*C*_q_-2), 165.34 (*C*_q_-5); IR (ATR) 1372, 1184, 1064, 1025, 729, 616 cm^−1^.

*Bis-thiadiazole* **8**: R_f_ 0.14 (5% acetone in DCM); colourless oil; ^1^H NMR 2.328 (quint, 2H, J 7.0, C-C*H*_2_-C), 2.726 (s, 6H, C*H*_3_), 3.445 (t, 4H, J 7.0, 2 C*H*_2_-S); ^13^C NMR 15.63 (*C*H_3_), 28.63 (C-*C*H_2_-C), 32.39 (2 *C*H_2_-S), 164.93 (*C*_q_-2), 165.10 (*C*_q_-5); IR (ATR) 1379, 1185, 1065, 1038, 759, 615 cm^−1^; HR-MS (HESI^+^) *m*/*z* calcd. for C_9_H_12_N_4_S_4_^+^ [M + H]^+^ 305.0018, found 305.0016, ∆ = −0.2 mDa.

*Bis-thiadiazole* **9**: R_f_ 0.14 (5% acetone in DCM); colourless solid; m. p. 93.4–93.9 °C; ^1^H NMR 1.968 (m, 4H, 2 C-C*H*_2_-C), 2.723 (s, 6H, C*H*_3_), 3.333 (m, 4H, 2 C*H*_2_-S); ^13^C NMR 15.64 (*C*H_3_), 28.20 (C-*C*H_2_-C), 33.29 (2 *C*H_2_-S), 164.92 (*C*_q_-5), 165.31 (*C*_q_-2); IR (ATR) 1389, 1194, 1068, 1048, 652, 608 cm^−1^; HR-MS (HESI^+^) *m*/*z* calcd. for C_10_H_14_N_4_S_4_^+^ [M + H]^+^ 319.0174, found 319.0175, ∆ = 0.1 mDa.

*Olefin* **10**: R_f_ 0.63 (5% acetone in DCM); colourless oil; ^1^H NMR 2.754 (s, 3H, C*H*_3_), 5.633 (d, 1H, ^3^J 9.4, C*H*_2_^cis^=), 5.681 (d, 1H, ^3^J 16.7, C*H*_2_^trans^=), 6.882 (dd, 1H, ^3^J_cis_ 9.4, ^3^J_trans_ 16.8, C*H*=); ^13^C NMR 15.72 (*C*H_3_), 120.30 (*C*H_2_=), 126.90 (*C*H=), 163.72 (*C*_q_-2), 165.81 (*C*_q_-5); IR (ATR) 1590, 1385, 1188, 1065, 1045, 953, 625, 603 cm^−1^; HR-MS (HESI^+^) *m*/*z* calcd. for C_5_H_6_N_2_S_2_^+^ [M + H]^+^ 159.0045, found 159.0045, ∆ = 0 mDa.

### 3.3. Reaction with α,ω-Dichloroalkanes

To a solution of 2-mercapto-5-methyl-1,3,4-thiadiazole (4 mmol) in α,ω-dichloroalkane (30 mL; 10 mL for **4b** and **5b**), a base (8 mmol) was added and the mixture was stirred at rt for 24 h (cf. Table 1). The solid residue (if formed) was filtered off and the solvent was removed in vacuo. The crude mixture was purified by flash chromatography on a silica gel with a gradient of polarity from DCM to 5% acetone in DCM.

*Chloride* **2b**: R_f_ 0.46 (1% acetone in DCM); colourless oil; ^1^H NMR 2.793 (s, 3H, C*H*_3_), 5.252 (s, 2H, C*H*_2_); ^13^C NMR 15.82 (*C*H_3_), 47.29 (*C*H_2_), 161.33 (*C*_q_-2), 166.91 (*C*_q_-5); IR (ATR) 1449, 1378, 1267, 1198, 1050, 739, 623 cm^−1^; HR-MS (HESI^+^) *m*/*z* calcd. for C_4_H_5_ClN_2_S_2_^+^ [M + H]^+^ 180.9655, found 180.9655, ∆ = 0 mDa.

*Chloride* **3b**: cf. ref. [84].

*Chloride* **4b**: R_f_ 0.69 (5% acetone in DCM); colourless oil; ^1^H NMR 2.293 (m, 2H, C-C*H*_2_-C), 2.727 (s, 3H, C*H*_3_), 3.453 (t, 2H, J 6.9, C*H*_2_-S), 3.692 (t, 2H, J 6.3, C*H*_2_-Cl); ^13^C NMR 15.63 (*C*H_3_), 30.89 (*C*H_2_-S), 31.69 (C-*C*H_2_-C), 43.12 (*C*H_2_-Cl), 164.84 (*C*_q_-2), 165.10 (*C*_q_-5); IR (ATR) 1441, 1268, 736 cm^−1^; HR-MS (HESI^+^) *m*/*z* calcd. for C_6_H_9_ClN_2_S_2_^+^ [M + H]^+^ 208.9968, found 208.1547, ∆ = 0 mDa.

*Chloride* **5b**: R_f_ 0.66 (5% acetone in DCM); colourless oil; ^1^H NMR 1.945 (m, 4H, 2 C-C*H*_2_-C), 2.724 (s, 3H, C*H*_3_), 3.337 (t, 2H, J 6.9, C*H*_2_-S), 3.573 (t, 2H, J 6.2, C*H*_2_-Cl); ^13^C NMR 15.62 (*C*H_3_), 26.54 (*C*H_2_-C-S), 31.33 (*C*H_2_-C-Cl), 33.13 (*C*H_2_-S), 44.24 (*C*H_2_-Cl), 164.92 (*C*_q_-5), 165.24 (*C*_q_-2); IR (ATR) 1432, 1382, 1187, 1070, 1035, 649 cm^−1^; HR-MS (HESI^+^) *m*/*z* calcd. for C_7_H_11_ClN_2_S_2_^+^ [M + H]^+^ 223.0125, found 223.0125, ∆ = 0 mDa.

### 3.4. Crystallography

Single crystals of compounds **2b**, **6**, **7**, and **9** with suitable size and diffracting quality were mounted on glass capillaries or nylon Cryoloop (Hampton research, Aliso Viejo, CA, USA). Diffraction data for **2b** were collected on a SupernovaDual diffractometer equipped with an Atlas CCD detector, while diffraction data for **6** were collected on a Bruker D8 Venture diffractometer equipped with a PHOTON II CPAD detector. Both diffractometers operate with a micro-focus sealed X-ray source, generating MoKα radiation (0.71073 Å). The collected data were processed with CrysAlisPro (41.117a-64bit) [88] (for **2b**) or APEX4 (2022.1-1) [89] (for **6**) programs. The structures were solved with intrinsic phasing methods and refined using the full-matrix least-squares method of *F*^2^ using ShelxT (2018.1) [90] and ShelxL [91] as implemented in the OLEX2-ver.1.5 graphical interface [92]. All non-hydrogen atoms were located successfully from a Fourier map and were refined anisotropically. Hydrogen atoms were placed on calculated positions riding on the parent carbon atoms (*U_eq_* = 1.2 for C-H_methyl_ = 0.93 Å and C-H_methylenic_ = 0.97 Å) while those riding on heteroatoms (N1 in **2b**) were placed from a difference Fourier map and refined freely. Ortep-3v2 software [93] was used to prepare the figures visualizing the molecules present in the in the asymmetric unit. Crystallography Open Database (COD) entries 3000465, 3000466, 3000493, and 3000494 contain the supplementary crystallographic data for this paper. These data, accessed on 30 January 2024, can be obtained free of charge via http://www.crystallography.net (accessed on 18 April 2024).

## 4. Conclusions

A series of 2-(ω-bromoalkylthio)-5-methyl-1,3,4-thiadiazoles, 2-(ω-chloroalkylthio)-5-methyl-1,3,4-thiadiazoles and symmetrical bis-thiadiazoles possessing variable chain lengths are obtained via optimised protocols and are characterised by 1D and 2D NMR spectra and by single crystal XRD of selected samples.

It is observed that the reaction output is strongly dependent both on the alkyl chain length and on the type of the halogen atoms. The symmetrical methylene-bridged bis-thiadiazole is isolated as a sole or major product from the reactions with dibromomethane and dichloromethane, respectively. Bromides and bis-thiadiazoles are formed as mixtures when using dibromoethane, dibromopropane, and dibromobutane; the ratios being dependent on the reagents’ proportions. Bromides are the predominant products when using a 2 molar excess of dibromides, while the opposite relation is achieved with a reversed ratio. Chlorides are isolated in quantitative yields from reactions with dichloroethane, dichloropropane, and dichlorobutane, used both as reagents and solvents.

Despite the fact that the chlorides **3b**–**5b** can be obtained quantitatively, the bromides **3a**–**5a** are also of interest because they are more reactive as alkylating agents. The obtained reagents offer unlimited opportunities for the preparation of targets by alkylation of diverse molecules. On the other hand, symmetrical bis-thiadiazoles, which are isolated in up to 80% yield, can find broad applications as polynuclear ligands in coordination chemistry, including in MOFs preparation.

## Figures and Tables

**Table 1:** Synthesis of the target reagents **2**–**5** and bis-thiadiazoles **6**–**9**; selected experiments.

Entry	Conditions ^a^	Products				
		Halogenide		Bis-Thiadiazole		Total Yield, %
		Compd.	Yield, %	Compd.	Yield, %	
1	DBM (8; 1:2), K_2_CO_3_, EtOH, reflux, 2.5 h	**2a**	-	**6**	76	76
2	DBM (8; 1:2), Et_3_N, ether	**2a**	-	**6**	74	74
3	DBM (2.2; 2:1.1), Et_3_N, ether	**2a**	-	**6**	75	75
4	DCM, DIPEA	**2b**	25	**6**	71	96
5	DCM, Et_3_N	**2b**	18	**6**	80	98
6	DBE (8; 1:2), K_2_CO_3_, MeCN	**3a**	13	**7**	30	55 ^b^
7	DBE (8; 1:2), Et_3_N, ether	**3a**	53	**7**	40	93
8	DBE (2.5; 2:1.25), Et_3_N, ether	**3a**	31	**7**	65	96
9	DBE (2.2; 2:1.1), Et_3_N, ether	**3a**	28	**7**	69	97
10	DCE, Et_3_N	**3b**	98	**7**	-	98 ^c^
11	DBP (8; 1:2), Et_3_N, ether	**4a**	63	**8**	33	96
12	DBP (2.5; 2:1.25), Et_3_N, ether	**4a**	35	**8**	59	94
13	DBP (2.2; 2:1.1), Et_3_N, ether	**4a**	25	**8**	73	98
14	DCP, Et_3_N	**4b**	93	**8**	-	93
15	DBB (8; 1:2), Et_3_N, ether	**5a**	66	**9**	31	97
16	DBB (2.5; 2:1.25), Et_3_N, ether	**5a**	29	**9**	58	87
17	DBB (2.2; 2:1.1), Et_3_N, ether	**5a**	8	**9**	78	86
18	DCB, Et_3_N	**5b**	98	**9**	-	98

^a^ A total of 4 mmols of thiadiazole **1** and 8 mmol of base (1:2 ratio) were used in all of the presented experiments. The dibromide amounts in mmol and thiadiazole/dibromide molar ratios are shown in parentheses. A total of 40 mL solvent were used in the reaction with dibromides; 20 mL for entry 1. Dichlorides were used as solvents; 30 mL DCM and DCE or 10 mL DCP and DCB. All reactions were carried out at rt for 24 h except entry 1. ^b^ Other isolated products: 12% of the corresponding olefin **10**. ^c^ Published in ref. [84].

## Data Availability

The data of the current study are available from the corresponding authors on reasonable request.

## Supplementary Materials

The following supporting information can be downloaded at: https://www.mdpi.com/article/10.3390/molecules29091938/s1, NMR data (Table S1), Crystallographic data collection and refinement parameters (Table S2), Crystallography (Figures S1 and S2), and original NMR, IR and HR-MS spectra (Section S1).
