# Supplementary material for: Functionalization of 2-Mercapto-5-methyl-1,3,4-thiadiazole: 2-(ω-Haloalkylthio) Thiadiazoles vs. Symmetrical Bis-Thiadiazoles
Source: Molecules. 2024 Apr 24;29(9):1938. doi: 10.3390/molecules29091938 (PMC11085375; doi:10.3390/molecules29091938)
Supplement: Supplementary file 1 [file molecules-29-01938-s001.zip › molecules-2948055-supplementary.pdf]

Supporting Information

Functionalization of 2-mercapto-5-methyl-1,3,4-thiadiazole: 2-(ω-haloalkylthio)-thiadiazoles vs symmetrical bis-thiadiazoles

Zhanina S. Petkova,[a] Rusi I. Rusew,[b] Boris L. Shivachev,[b]* and Vanya B. Kurteva[a]*

[a] Institute of Organic Chemistry with Centre of Phytochemistry, Bulgarian Academy of Sciences, Acad. G. Bonchev str., bl. 9, 1113 Sofia, Bulgaria; vanya.kurteva@orgchm.bas.bg

[b] Institute of Mineralogy and Crystallography "Acad. Ivan Kostov", Bulgarian Academy of Sciences, Acad. G. Bonchev str., bl. 107, 1113 Sofia, Bulgaria; blshivachev@gmail.com

Table of content

**Content**	**Page**
Table S1	2
Table S2	3
Figure S1	4
Figure S2	4
**Section S1**	5
NMR spectra of compound **3a**	5
NMR spectra of compound **4a**	7
NMR spectra of compound **5a**	9
NMR spectra of compound **6**	11
NMR spectra of compound **7**	12
NMR spectra of compound **8**	14
NMR spectra of compound **9**	16
NMR spectra of compound **10**	18
NMR spectra of compound **2b**	19
NMR spectra of compound **4b**	21
NMR spectra of compound **5b**	23
IR (ATR) spectra of the products	25
HR-MS (HESI$^+$) spectra of the products	31

Table S1. Methylene groups' signals in the NMR spectra of the products.

Compd.	CH_2-S	CH_2-C-S	CH_2-C-X	CH_2-X
**2b**	5.252/47.29			
**6**	5.197/36.88			
**3a**	3.724/35.18			3.743/29.69
**3b**	3.648/35.36			3.901/42.33
**7**	3.759/33.30			
**4a**	3.451/32.09	2.372/31.72		3.551/31.66
**4b**	3.453/30.89	2.293/31.69		3.692/43.12
**8**	3.445/32.39	2.328/28.63		
**5a**	3.337/32.97	1.970/27.78	2.032/31.47	3.438/32.76
**5b**	3.337/33.13	1.945 (common)/26.54	1.945 (common)/31.33	3.573/44.24
**9**	3.333/33.29	1.968/28.20		

Table S2. Most important data collection and crystallographic refinement parameters for compounds **2b** (as HCl salt), **6**, **7** and **9**.

Compound	**2b (HCl)**	**6**	**7**	**9**
Empirical formula	$C_4H_6Cl_2N_2S_2$	$C_7H_8N_4S_4$	$C_8H_{10}N_4S_4$	$C_{10}H_{14}N_4S_4$
Formula weight	217.13	276.41	290.44	318.49
Temperature/K	290	290	295.00	290.00
Crystal system	Monoclinic	Monoclinic	Monoclinic	Triclinic
Space group	$P2_1/c$	$P2_1/c$	$P2_1/c$	*P–1*
a/Å	8.3182(6)	13.6976(9)	12.3542(11)	5.640(3)
b/Å	11.8171(7)	5.6120(3)	5.4771(5)	6.849(4)
c/Å	9.5657(6)	16.5588(11)	9.8294(8)	9.469(6)
α/°	90	90	90	99.56(2)
β/°	114.594(9)	114.237(2)	113.270(2)	103.889(19)
γ/°	90	90	90	90.88(2)
Volume/$Å^3$	854.98(11)	1160.69(13)	611.00(9)	349.5(4)
Z	4	4	2	1
$\rho_{calc}g/cm^3$	1.687	1.582	1.579	1.513
μ/mm^{-1}	1.174	0.790	0.754	0.667
$F_{(000)}$	440.0	568.0	300.0	166.0
Crystal size/mm^3	0.35×0.35×0.35	0.4×0.1×0.08	0.22 × 0.2 × 0.15	0.22 × 0.19 × 0.18
Radiation	Mo *K*α λ = 0.71073	Mo *K*α λ = 0.71073	Mo *K*α λ = 0.71073	Mo *K*α λ = 0.71073
2Θ range for data collection/°	5.816 – 58.898	5.396 to 50.752	8.262 to 52.852	4.5 to 52.996
Reflections collected/independent	4819/2089	30650/2116	16853/1248	4686/1422
Index ranges R_{int} $I>=2\sigma(I)$/all data	0.0267/0.0308	0.0491/0.0190	0.0536/0.0349	0.0322/0.0335
Reflections/restraints/parameters	2089/0/97	2116/0/139	1248/0/83	1422/0/83
Goodness-of-fit on F^2	1.039	1.064	1.080	1.161
Final R indexes [$I>=2\sigma(I)$]	R_1 = 0.0274, wR_2 = 0.0692	R_1 = 0.0297, wR_2 = 0.0687	R_1 = 0.0392, wR_2 = 0.0970	R_1 = 0.0504, wR_2 = 0.1140
Final R indexes [all data]	R_1 = 0.0331, wR_2 = 0.0741	R_1 = 0.0345, wR_2 = 0.0722	R_1 = 0.0409, wR_2 = 0.0991	R_1 = 0.0596, wR_2 0.1178
Largest diff. peak/hole/e $Å^{-3}$	0.29/-0.29	0.52/-0.49	0.49/-0.30	0.48/-0.35
COD number	3000465	3000466	3000493	3000494

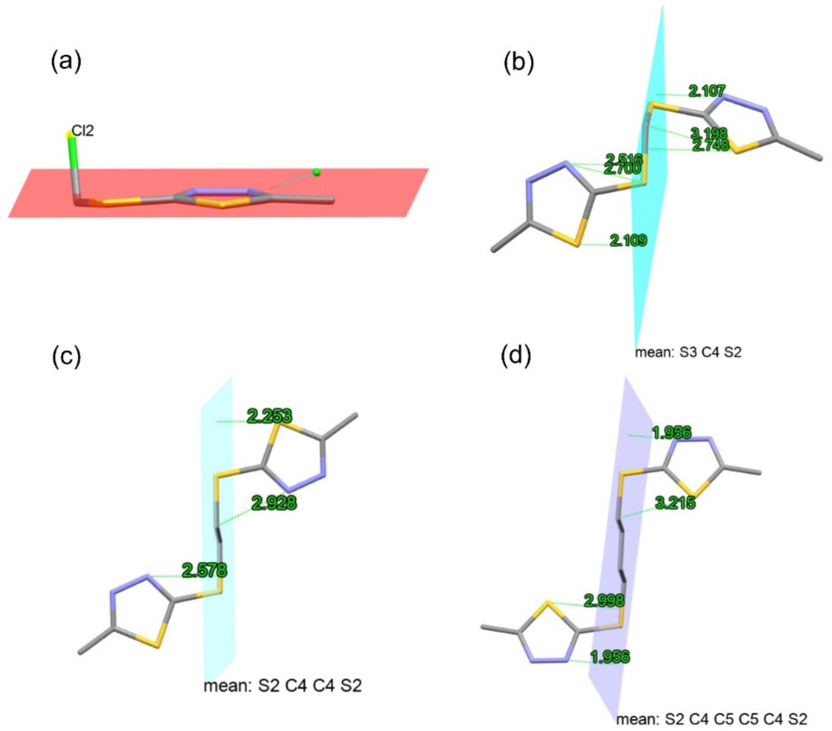

Figure S1. Depiction of (a) the observed planarity of the 1,3,4-thiadiazole motif in **2b** (b) the different rotation of the 1,3,4-thiadiazole moiety with respect to the S–C–S spacer plane in **6**, (c) and (d) the “analogical” orientation of the 1,3,4-thiadiazole motif with respect to the S–C–C–S and S–C–C–C–C–S planes in **7** and **9** although in **7** the N atoms are interacting with the spacer while in **9** the S atoms are the interacting with the spacer.

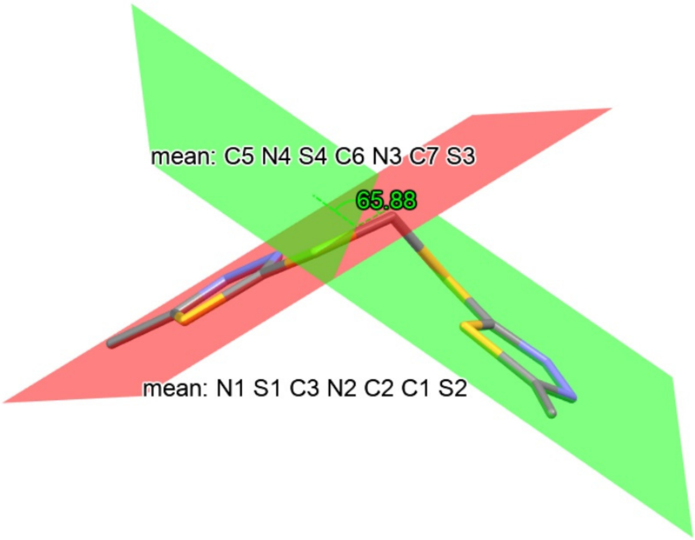

Figure S2. Depiction of the molecular geometry of **6** disclosing the planarity of the 1,3,4-thiadiazole ring and the angle their relative orientation (angle between the mean planes of 65.88°).

Section S1

NMR spectra of compound 3a

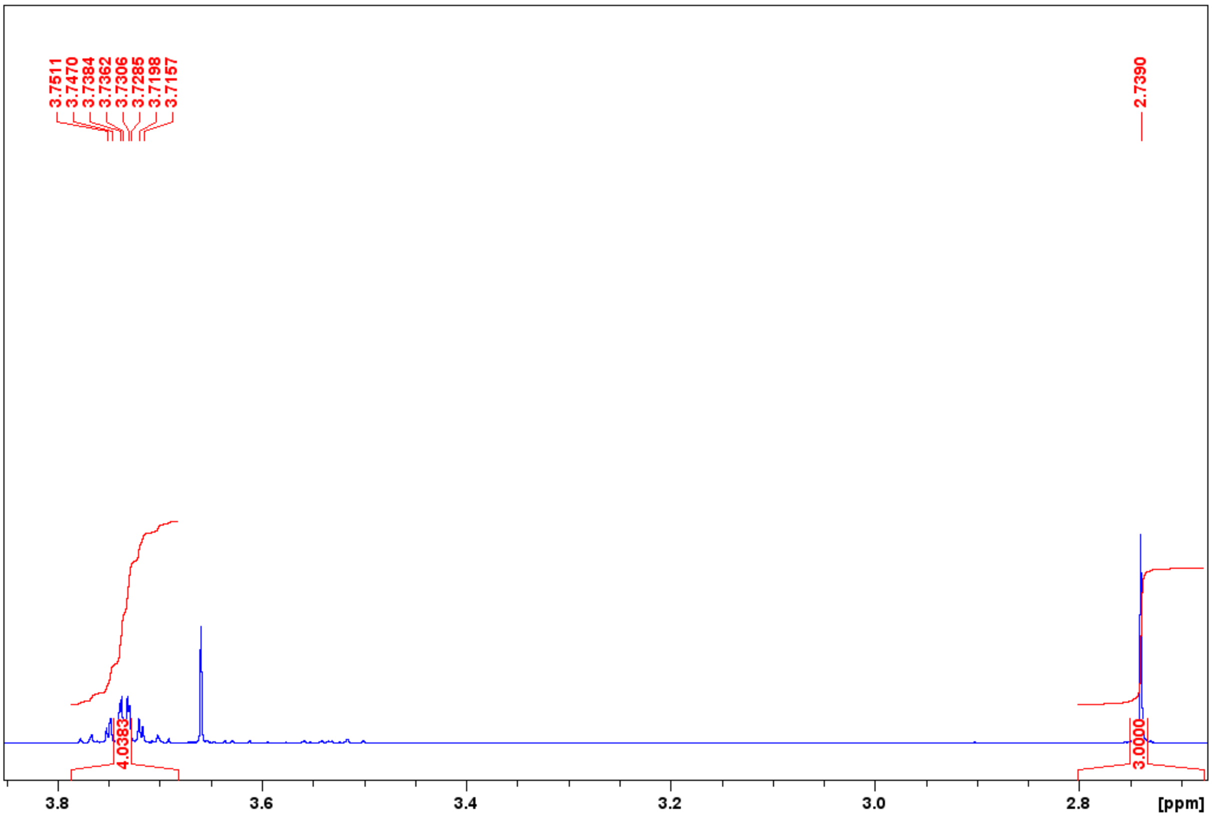

^{1}H NMR spectrum of compound **3a**.

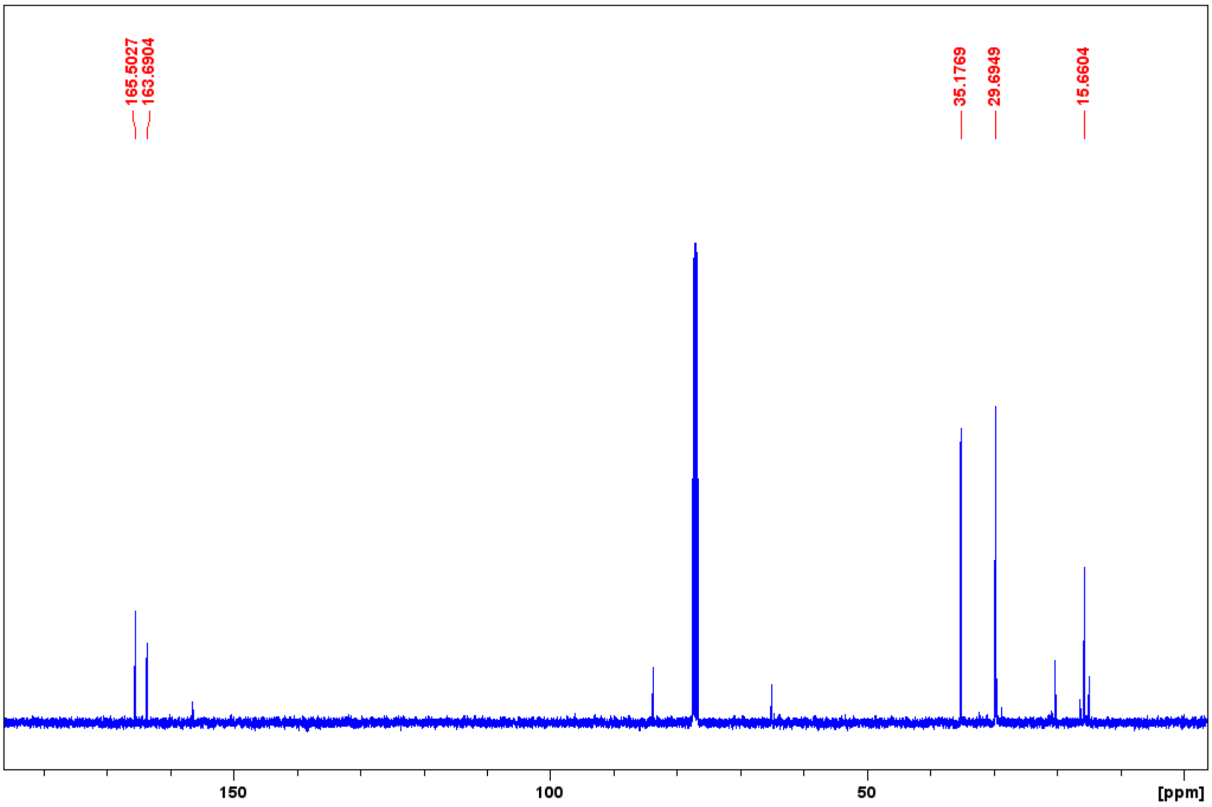

^{13}C NMR spectrum of compound **3a**.

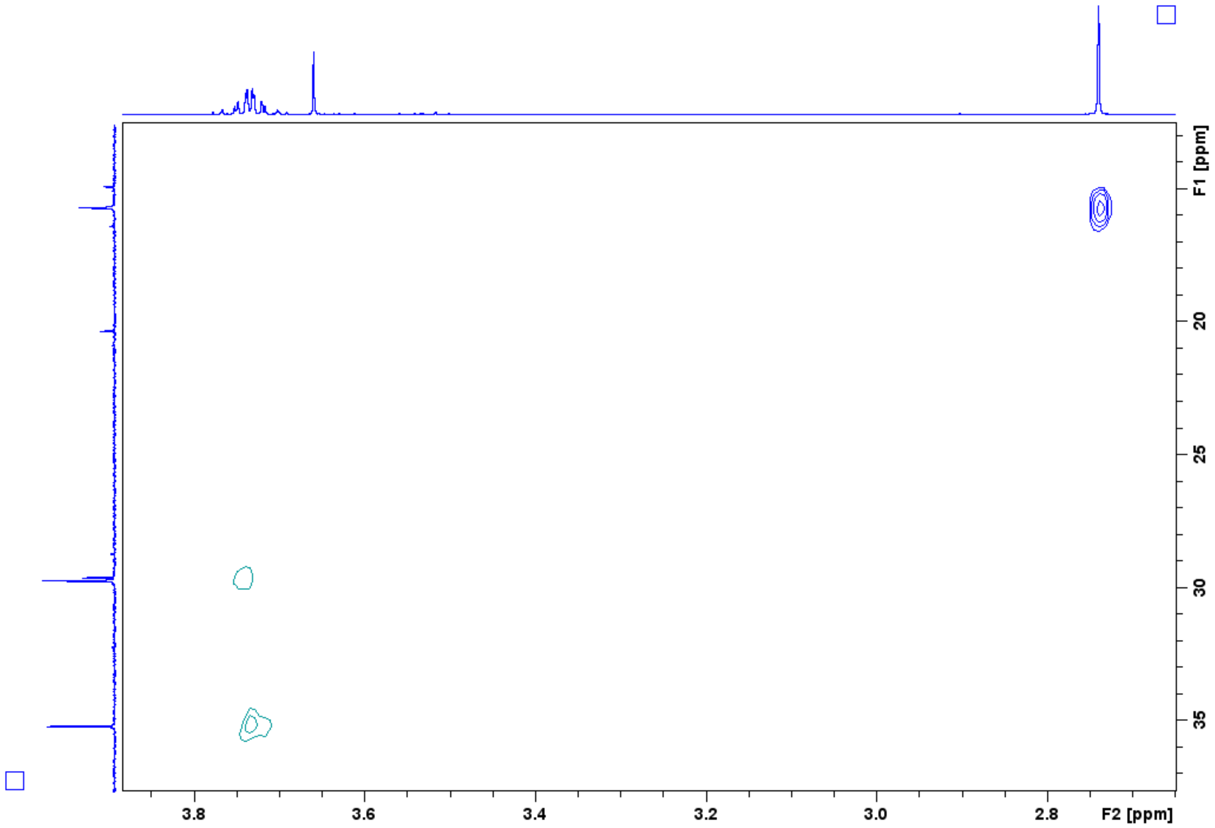

^{1}H-^{13}C HSQC spectrum of compound **3a**.

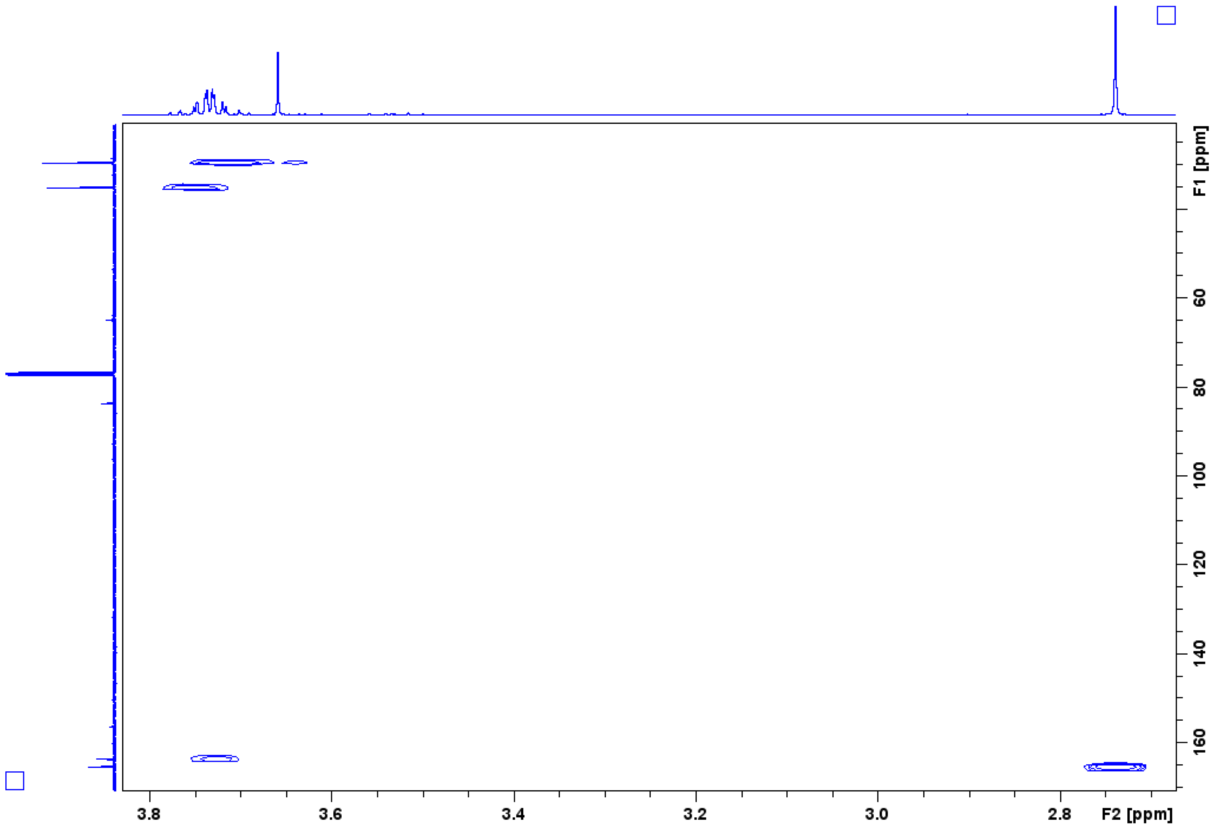

^{1}H-^{13}C HMBC spectrum of compound **3a**.

NMR spectra of compound 4a

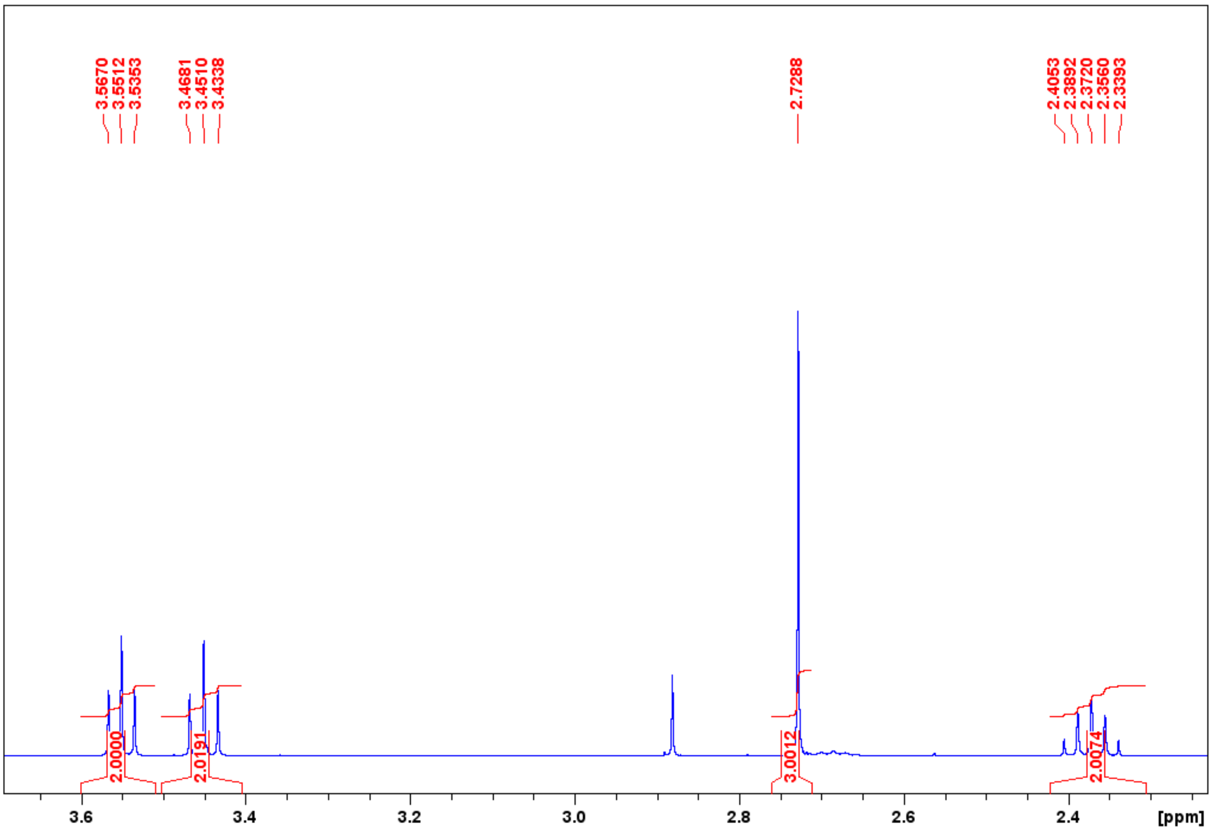

^{1}H NMR spectrum of compound **4a**.

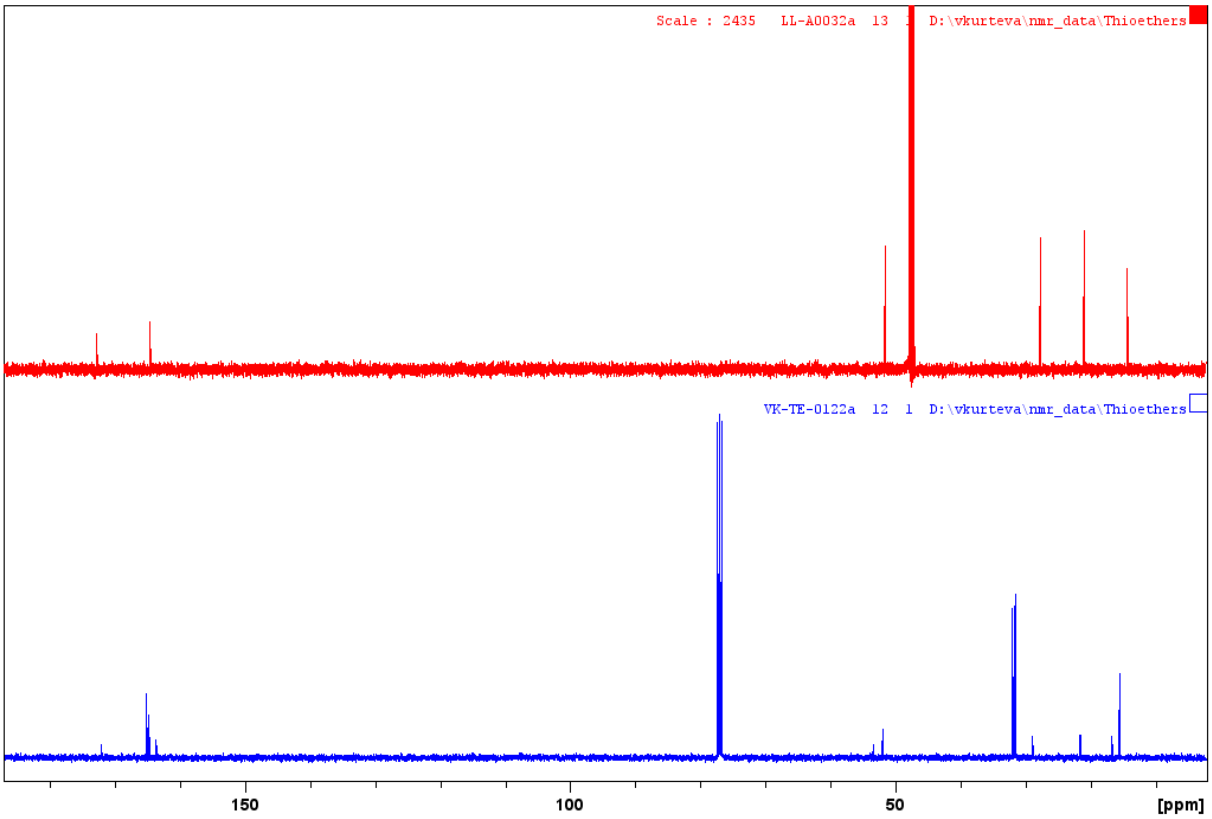

^{13}C NMR spectrum of compound **4a**; contaminated sample in $CDCl_3$ (blue) and pure one in CD_3OD (red).

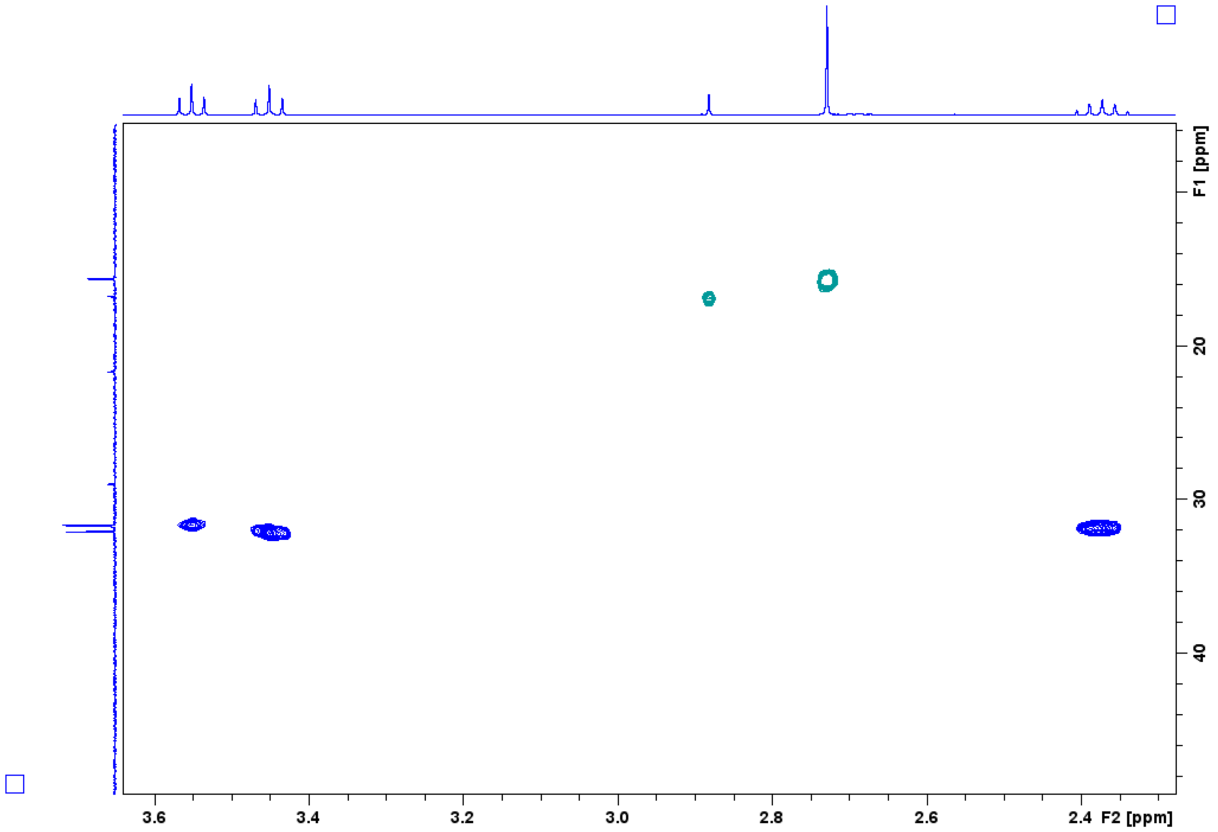

^{1}H-^{13}C HSQC spectrum of compound **4a**.

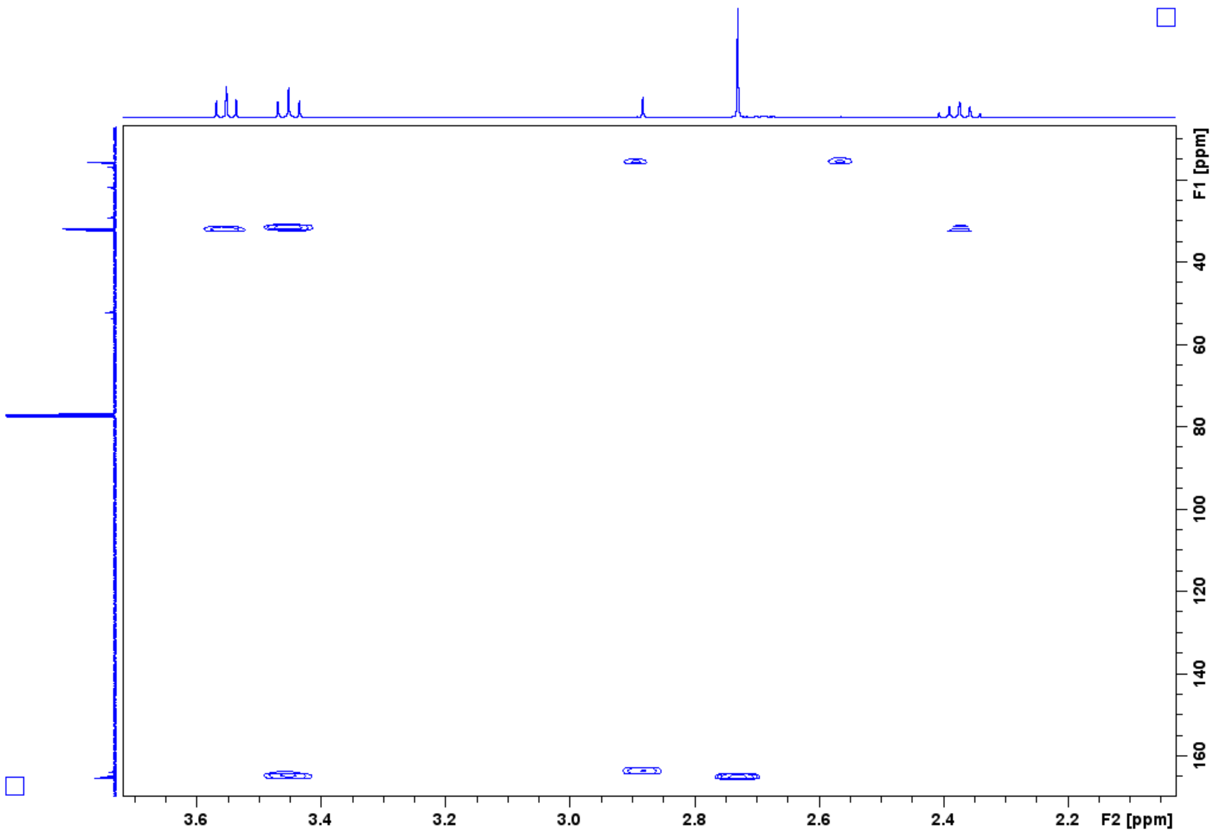

^{1}H-^{13}C HMBC spectrum of compound **4a**.

NMR spectra of compound 5a

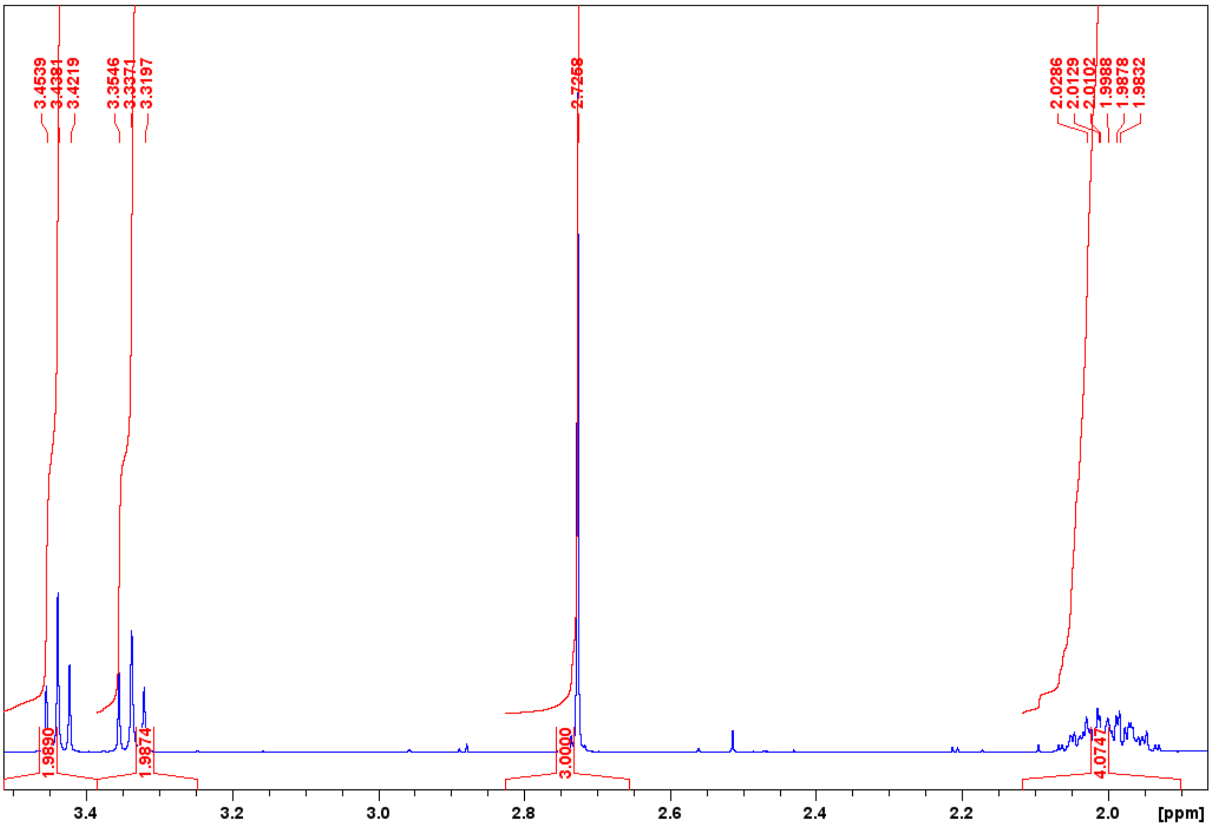

^{1}H NMR spectrum of compound **5a**.

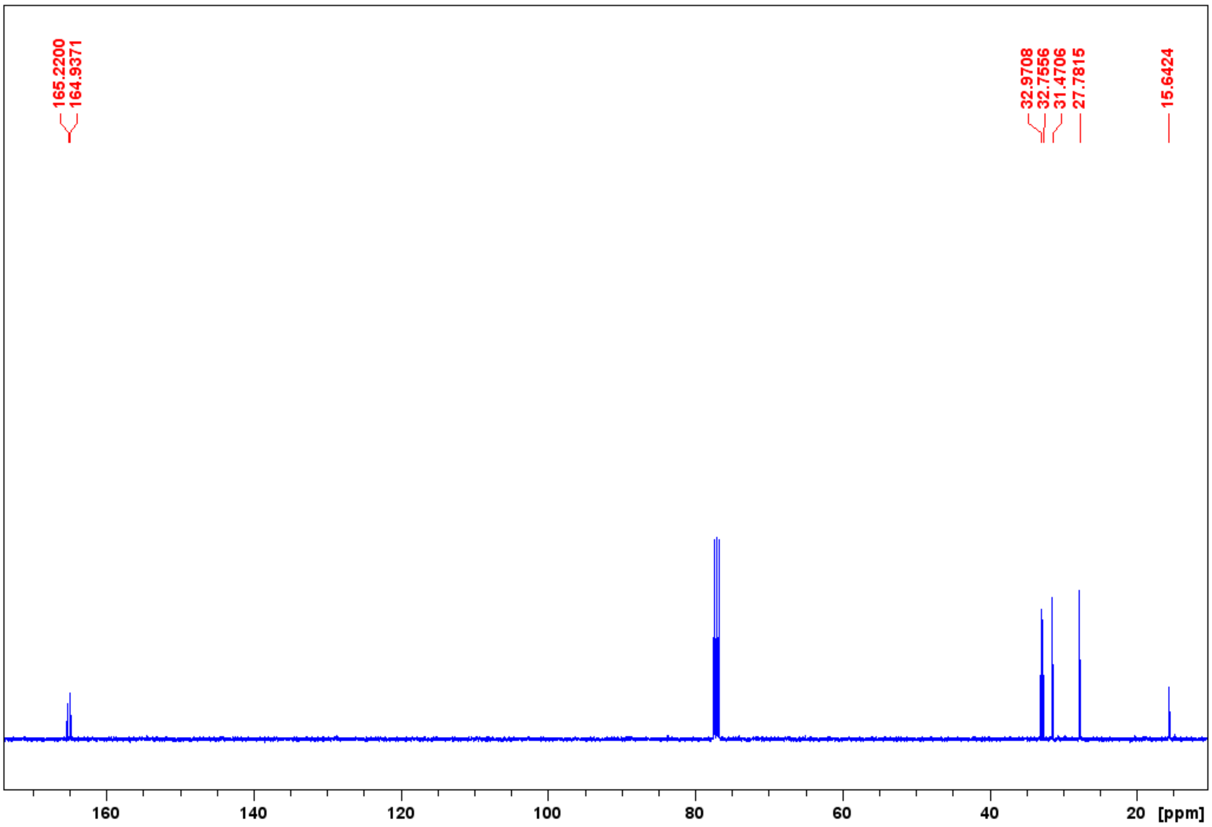

^{13}C NMR spectrum of compound **5a**.

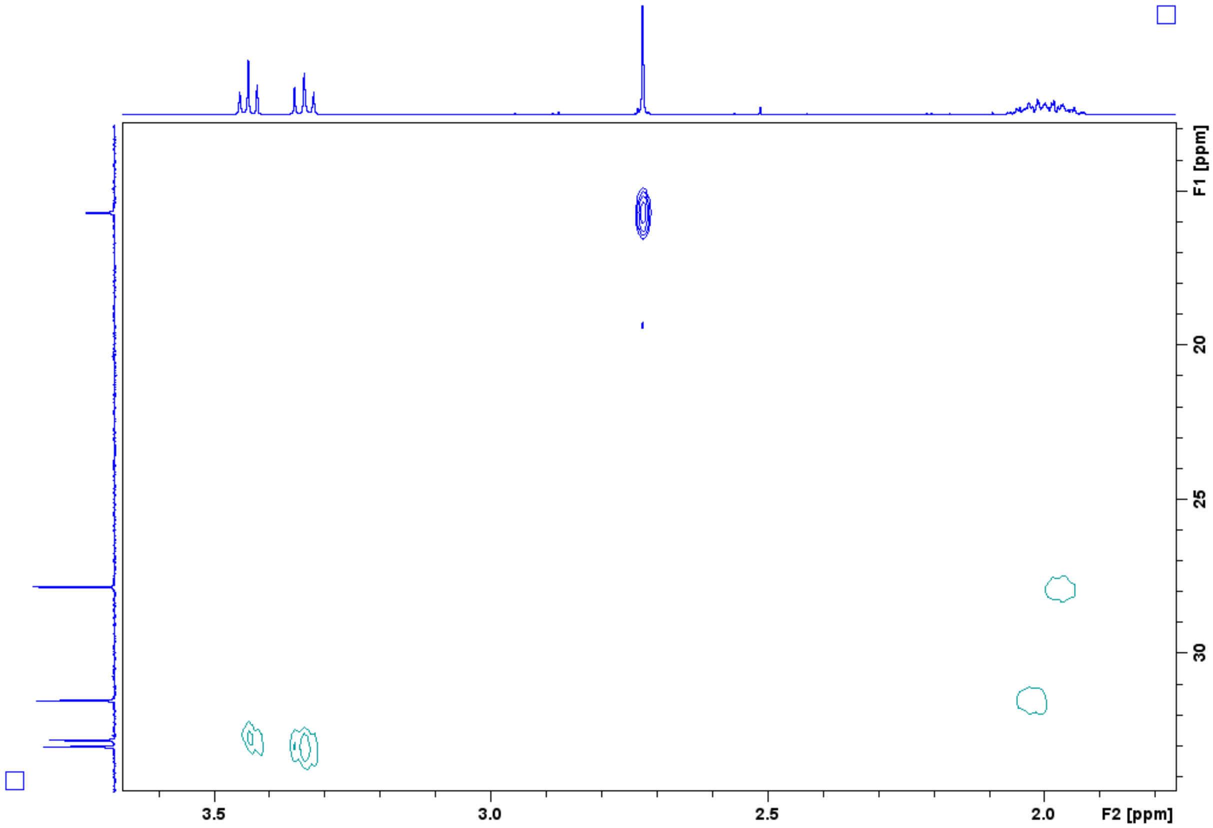

^1H-^{13}C HSQC spectrum of compound **5a**.

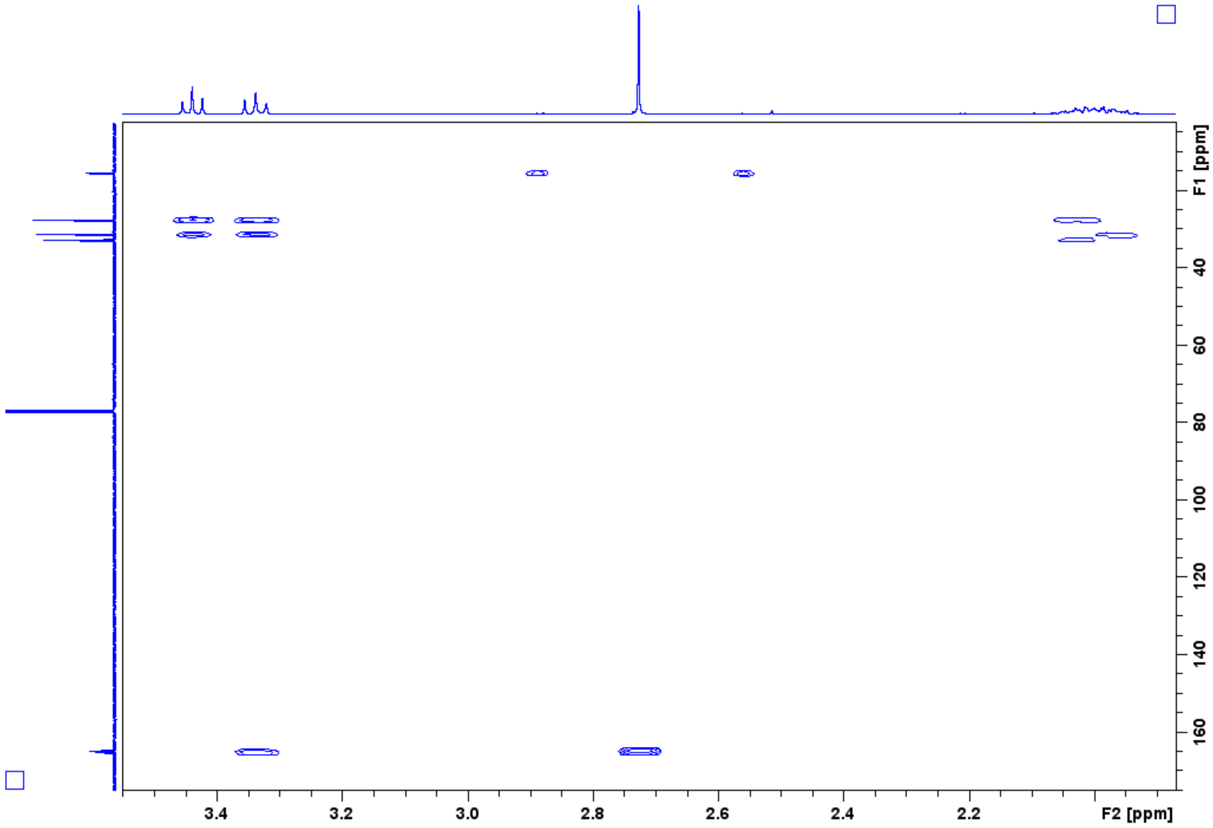

^1H-^{13}C HMBC spectrum of compound **5a**.

NMR spectra of compound 6

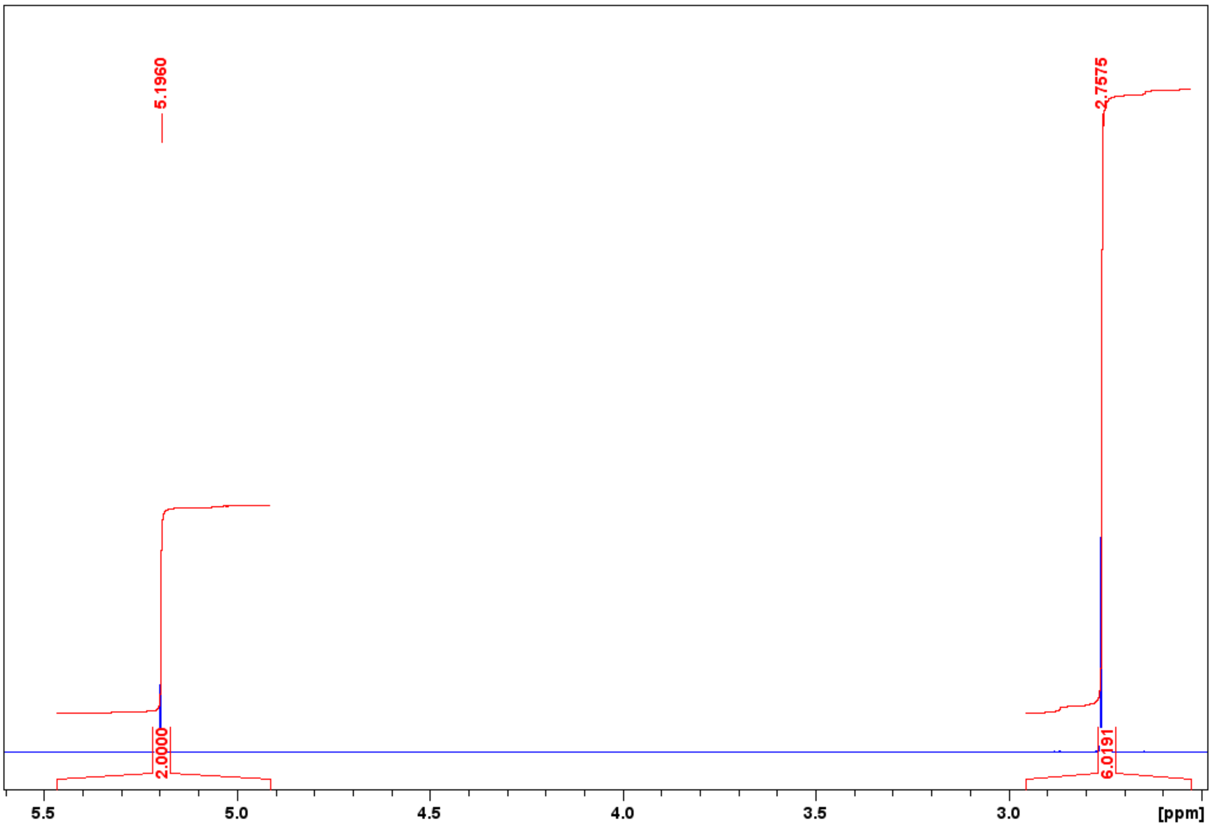

^{1}H NMR spectrum of compound **6**.

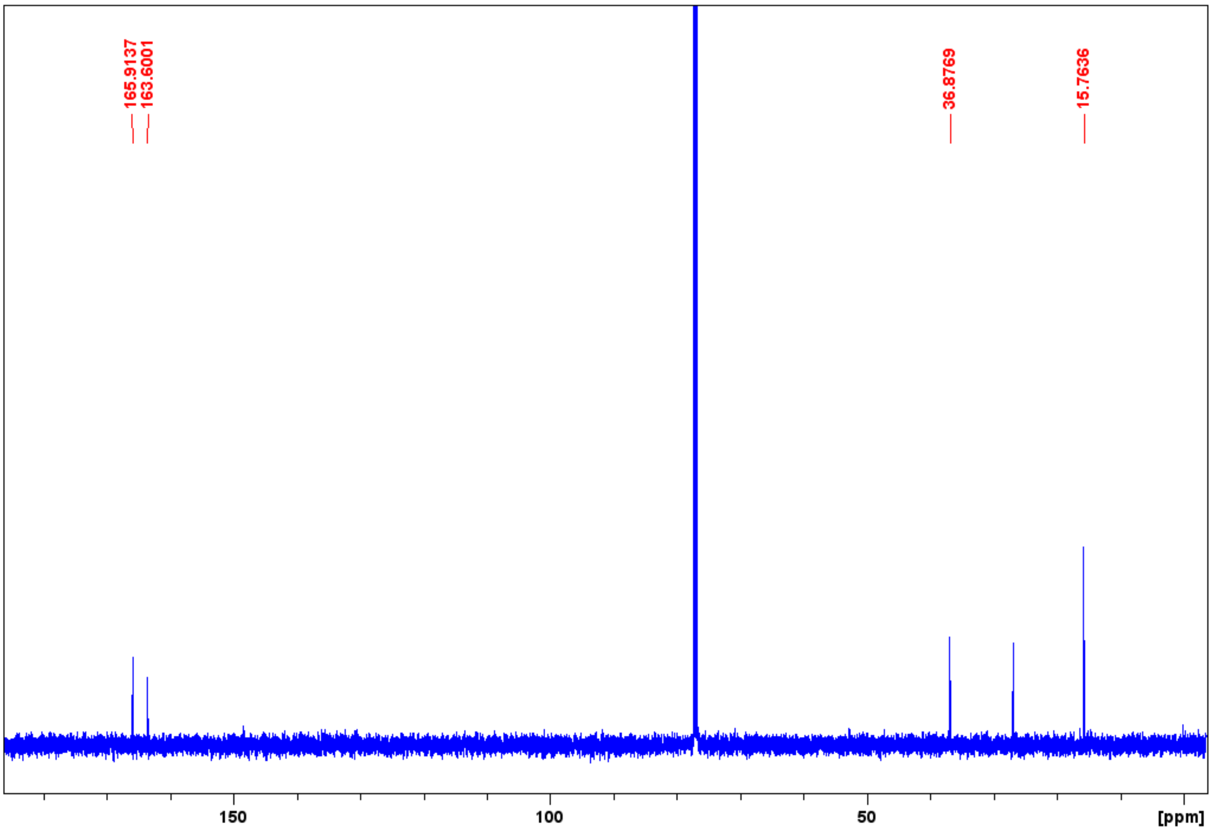

^{13}C NMR spectrum of compound **6**.

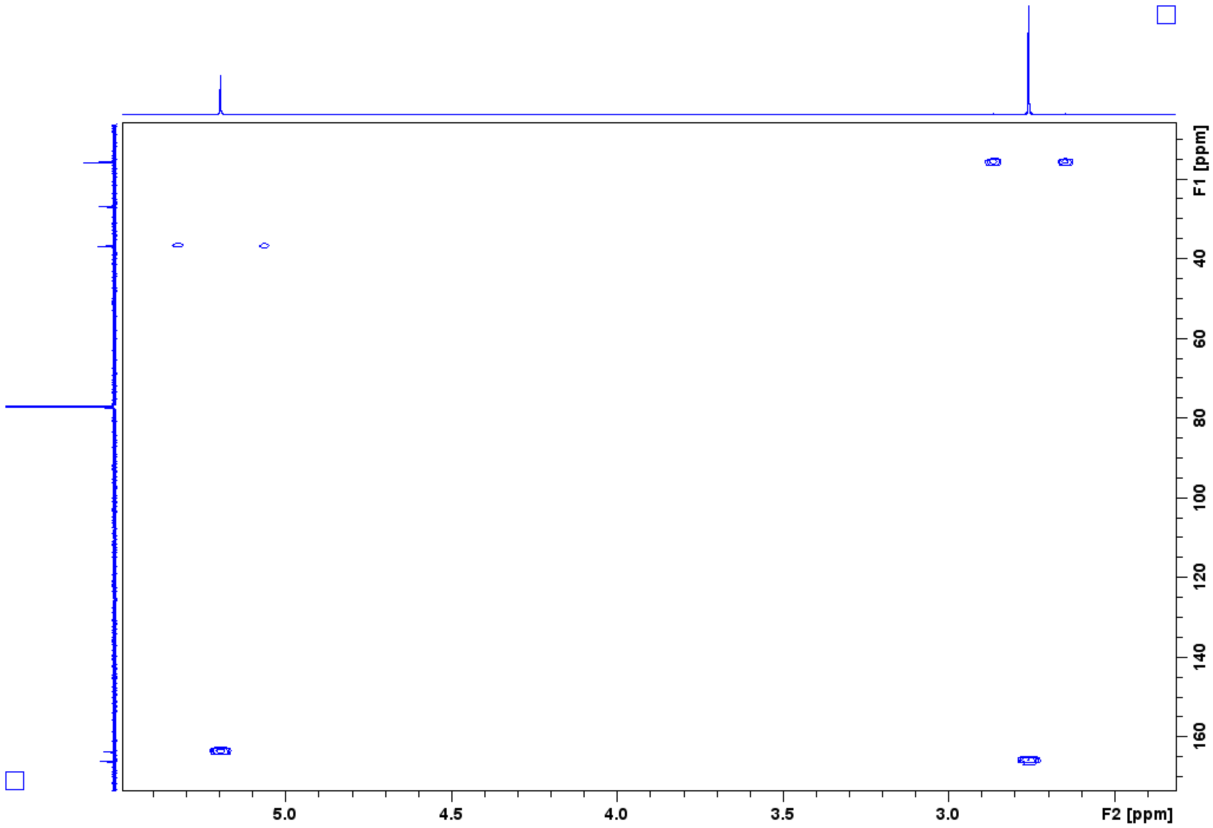

^{1}H-^{13}C HMBC spectrum of compound **6**.

NMR spectra of compound 7

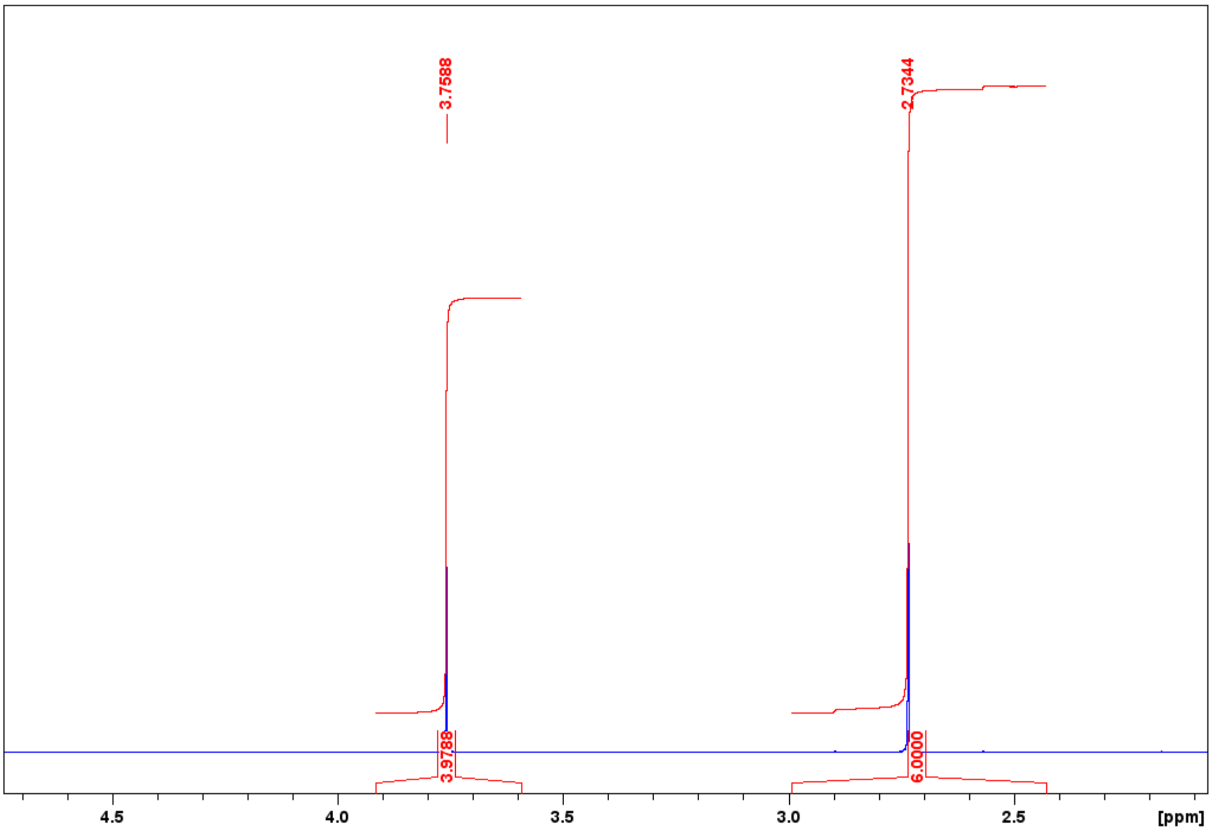

^{1}H NMR spectrum of compound **7**.

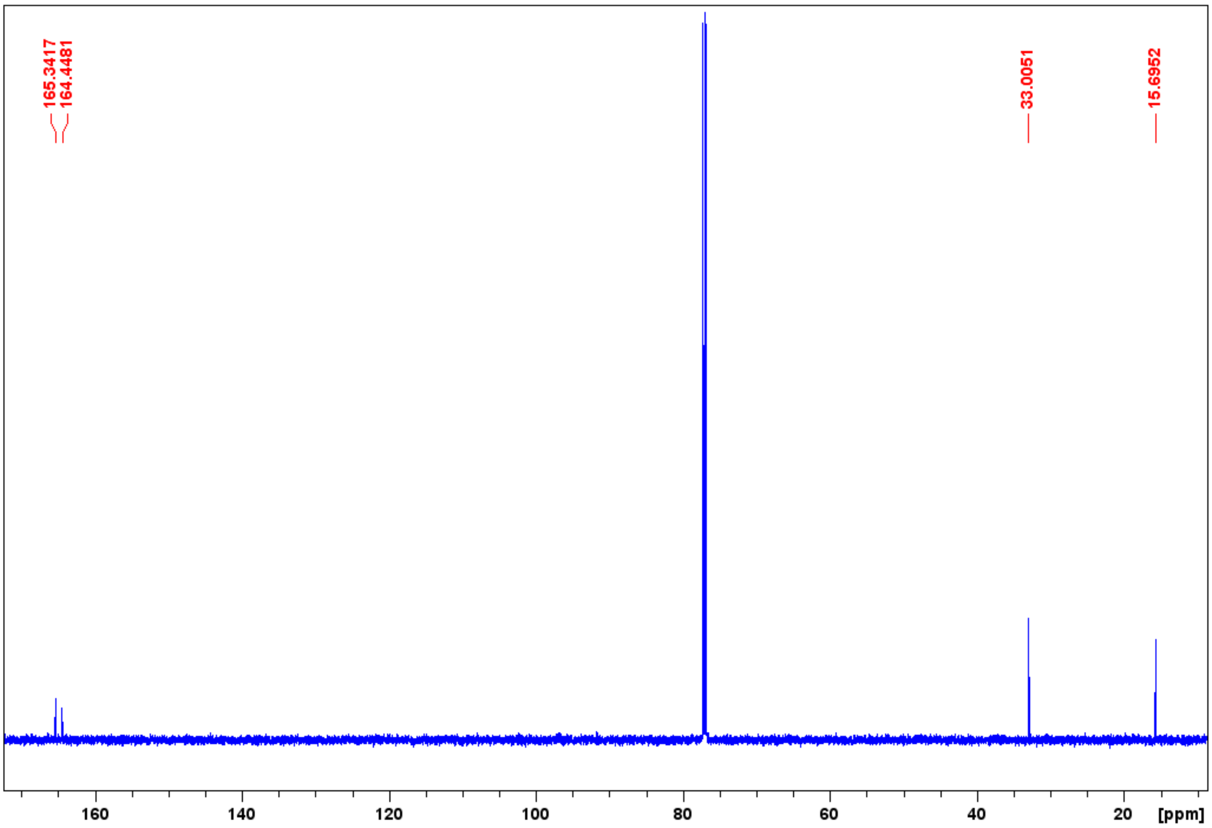

^{13}C NMR spectrum of compound **7**.

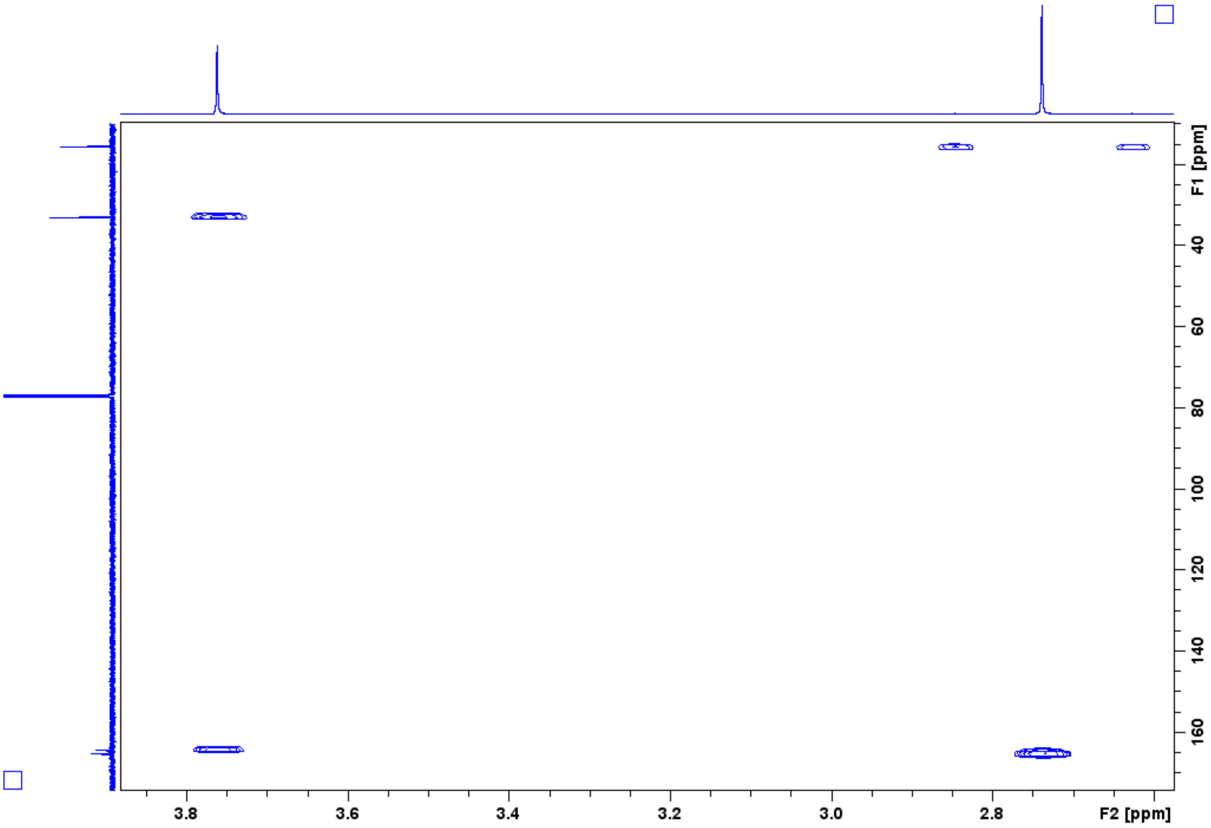

^{1}H-^{13}C HMBC spectrum of compound **7**.

NMR spectra of compound 8

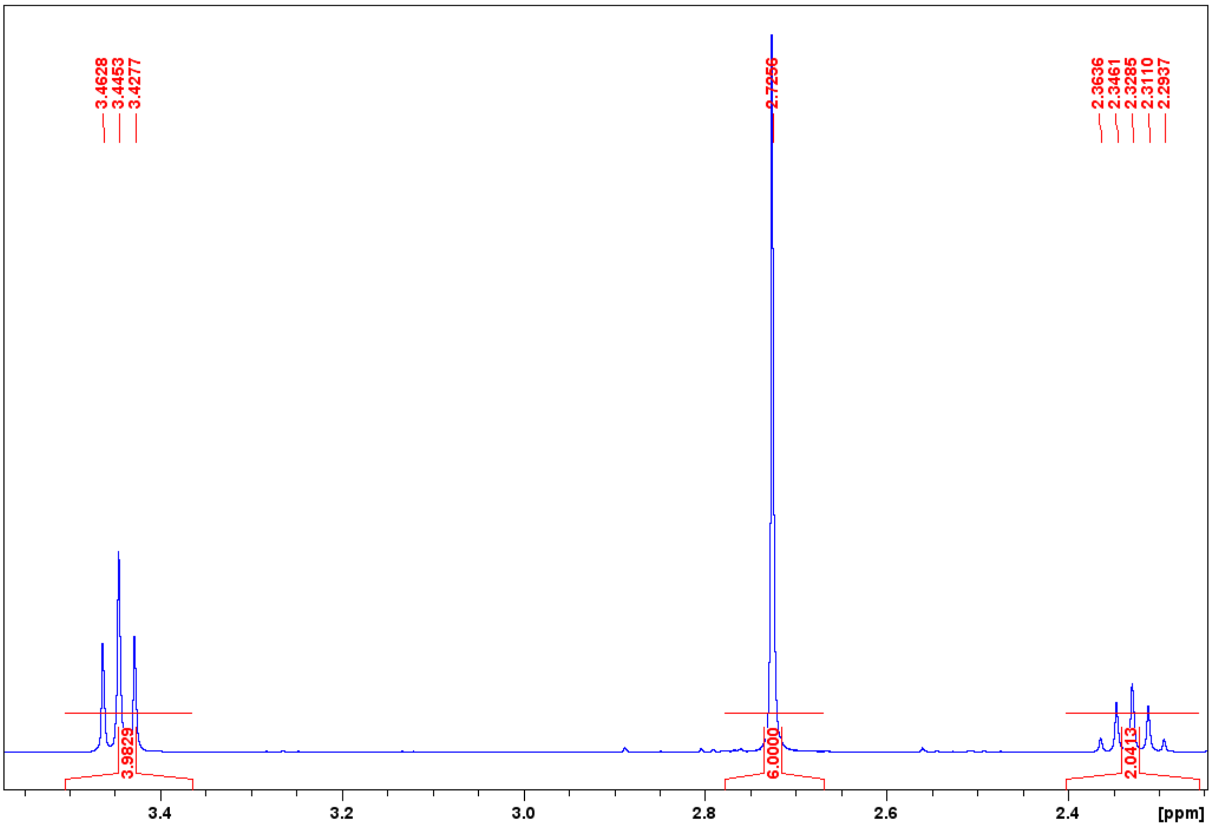

^{1}H NMR spectrum of compound **8**.

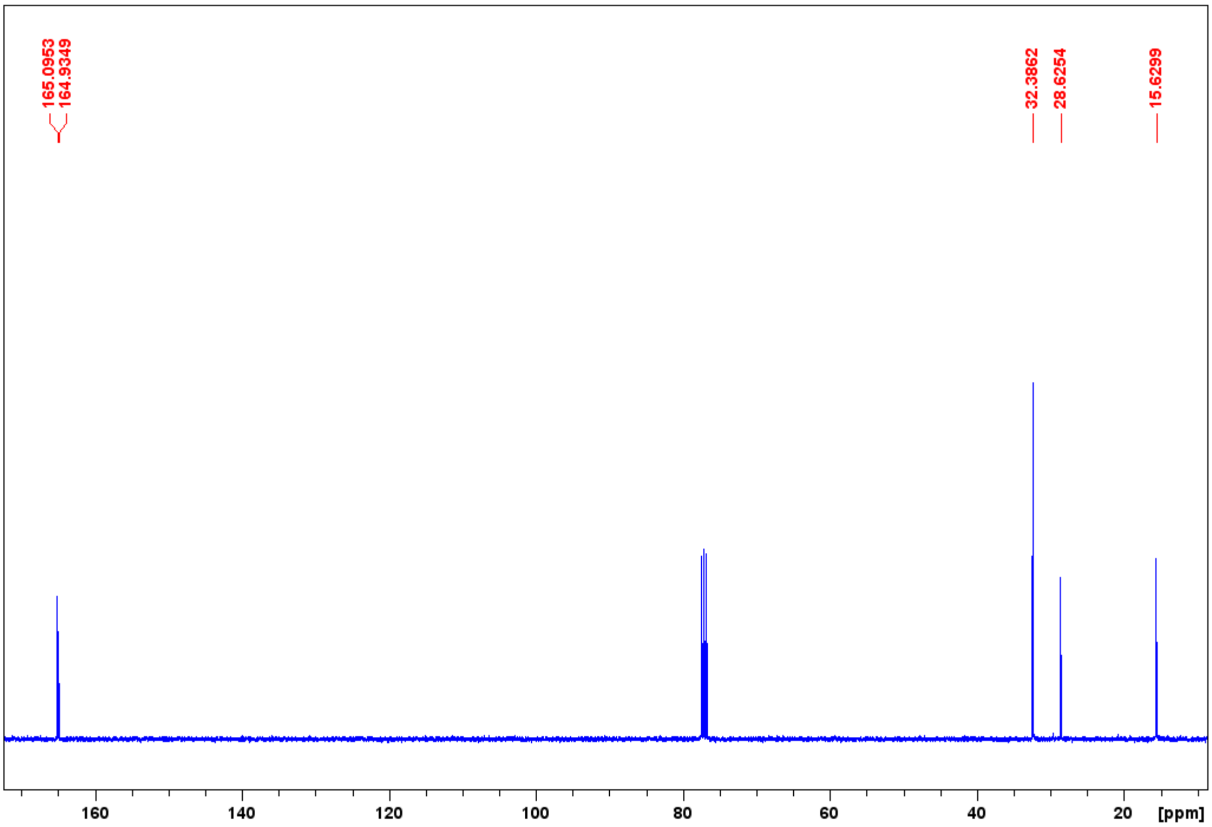

^{13}C NMR spectrum of compound **8**.

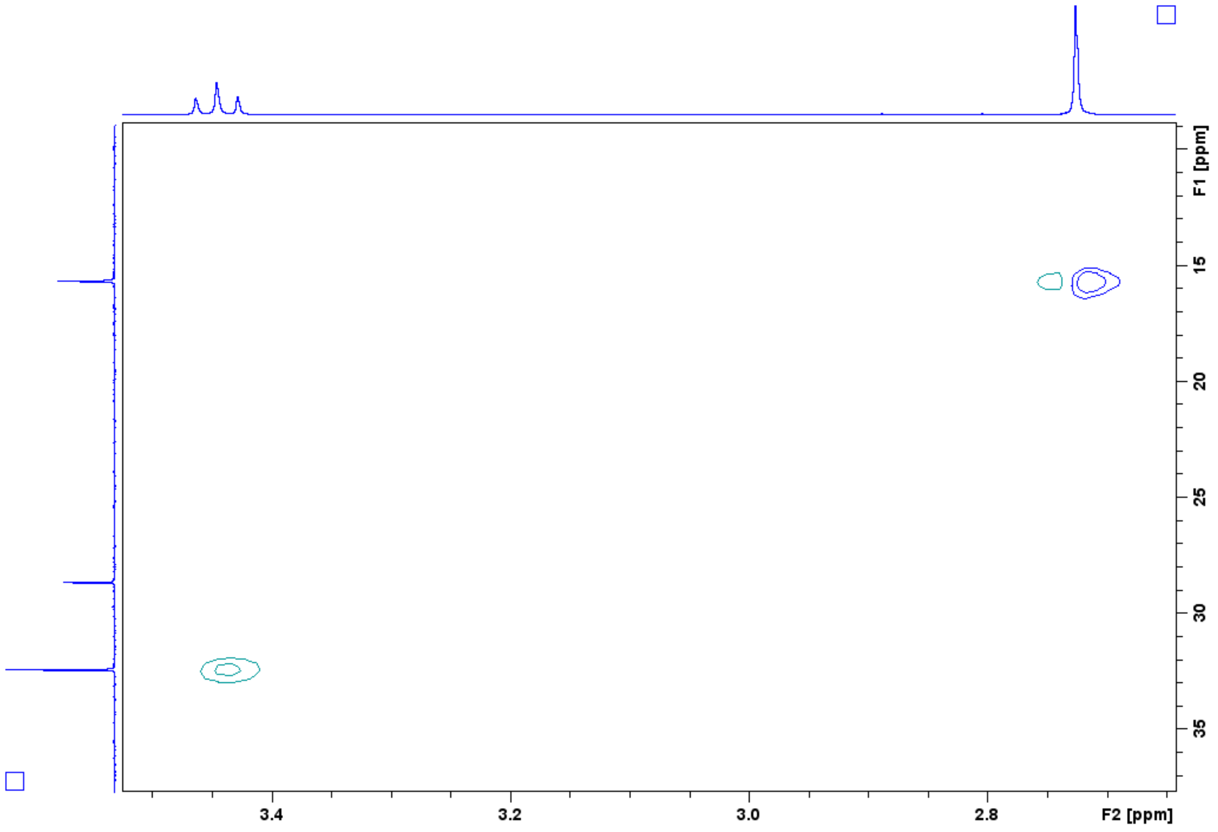

^1H-^{13}C HSQC spectrum of compound **8**.

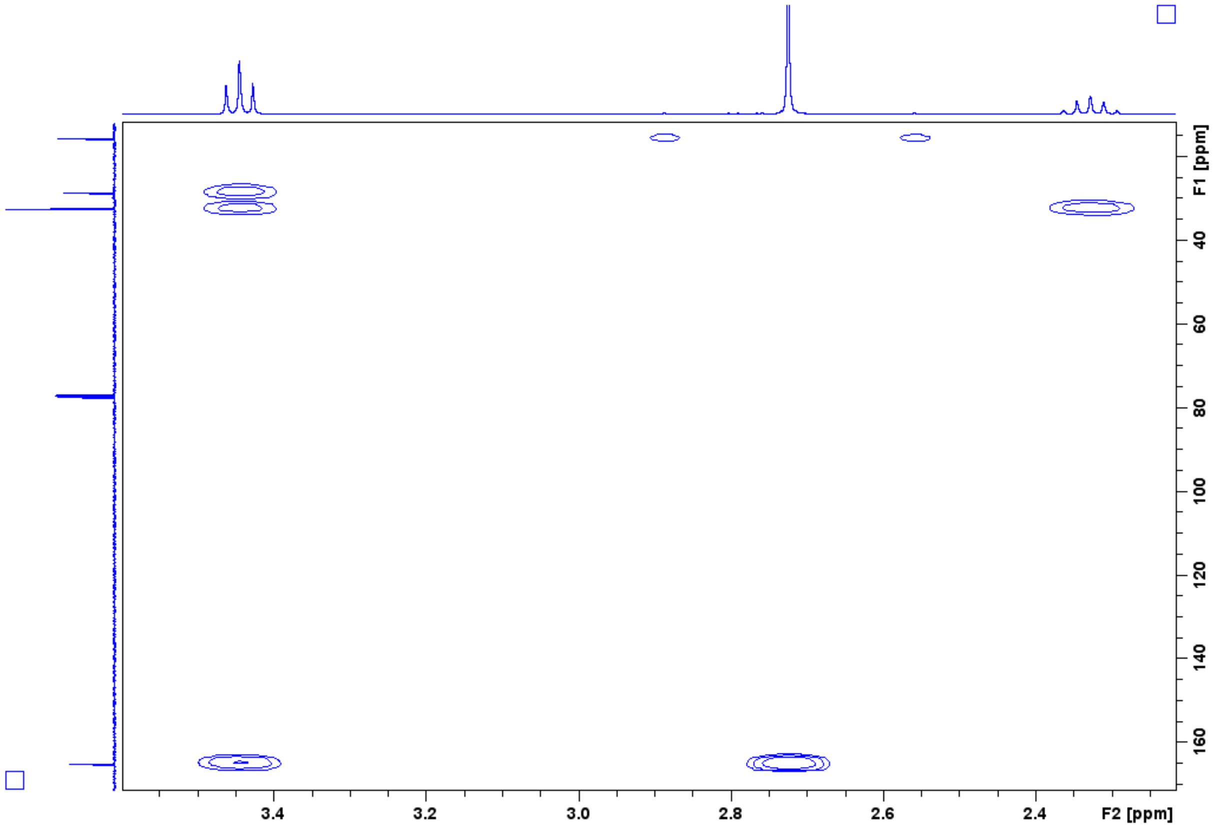

^1H-^{13}C HMBC spectrum of compound **8**.

NMR spectra of compound 9

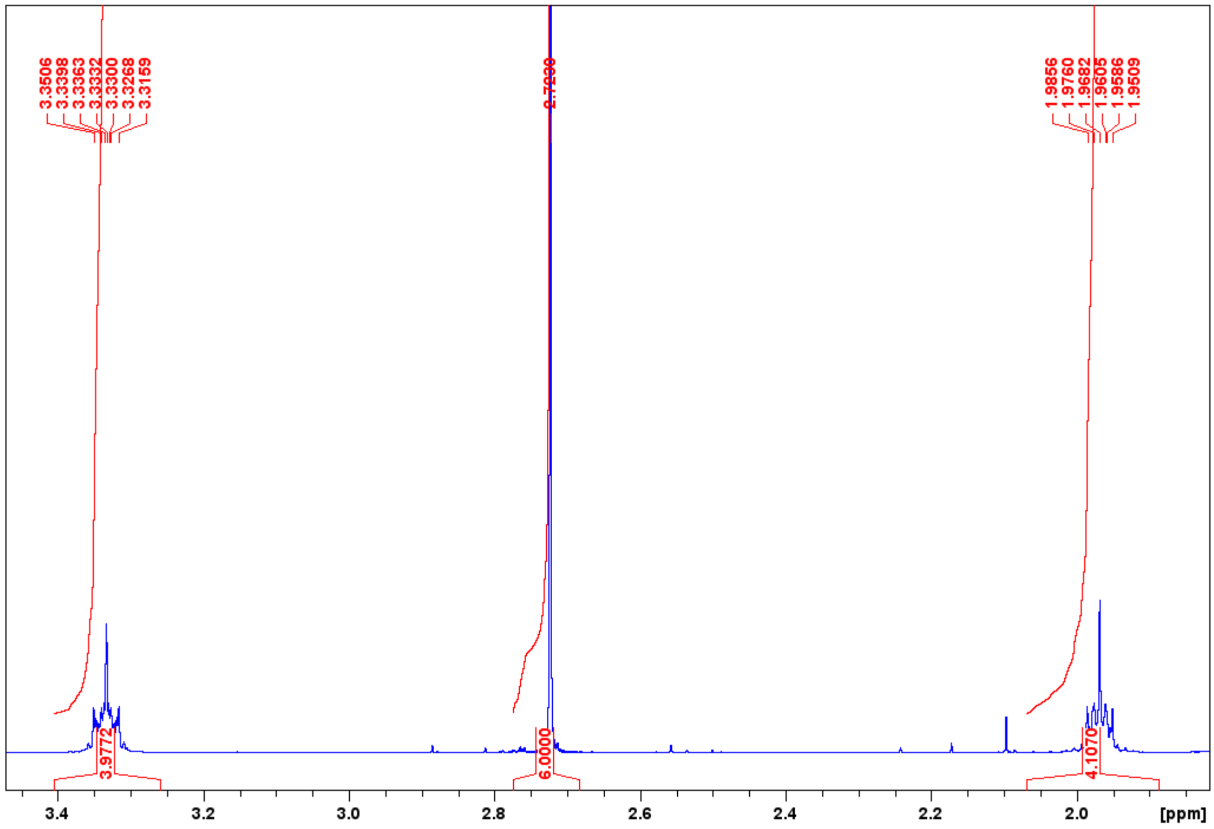

^{1}H NMR spectrum of compound **9**.

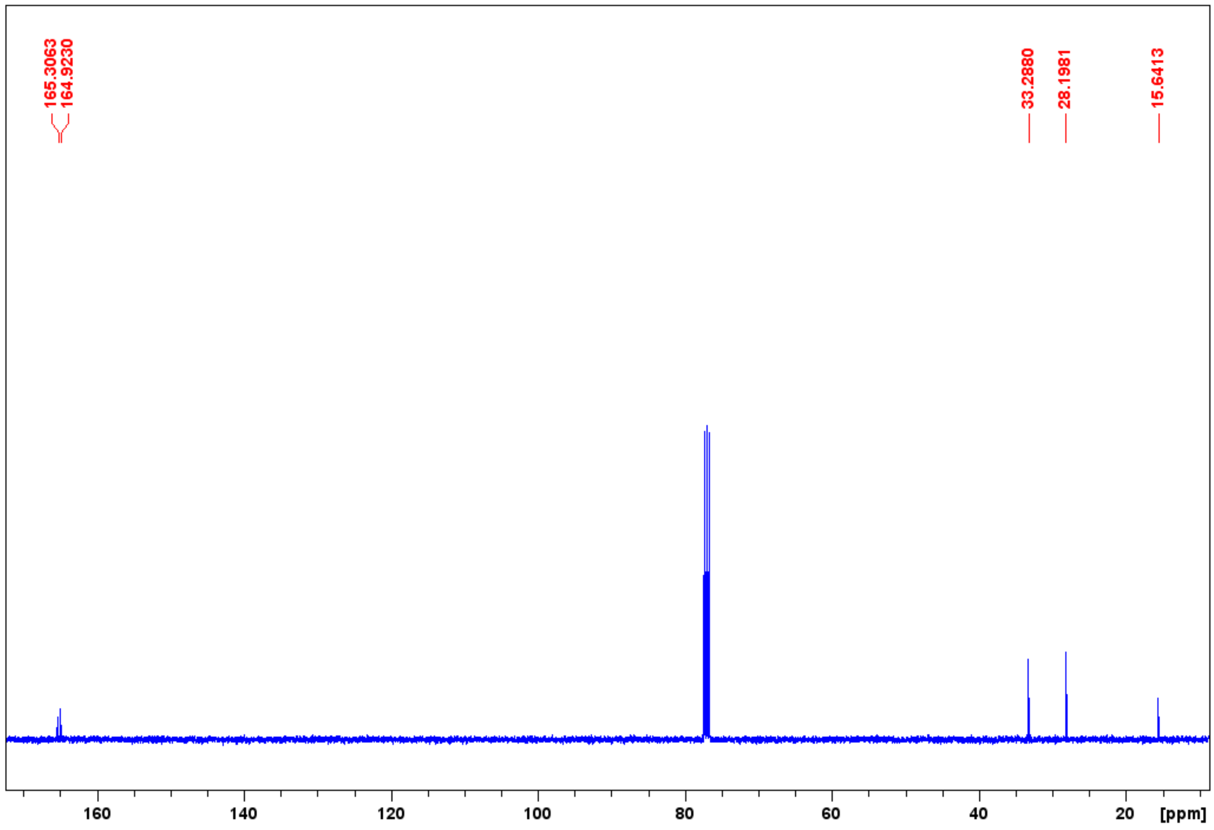

^{13}C NMR spectrum of compound **9**.

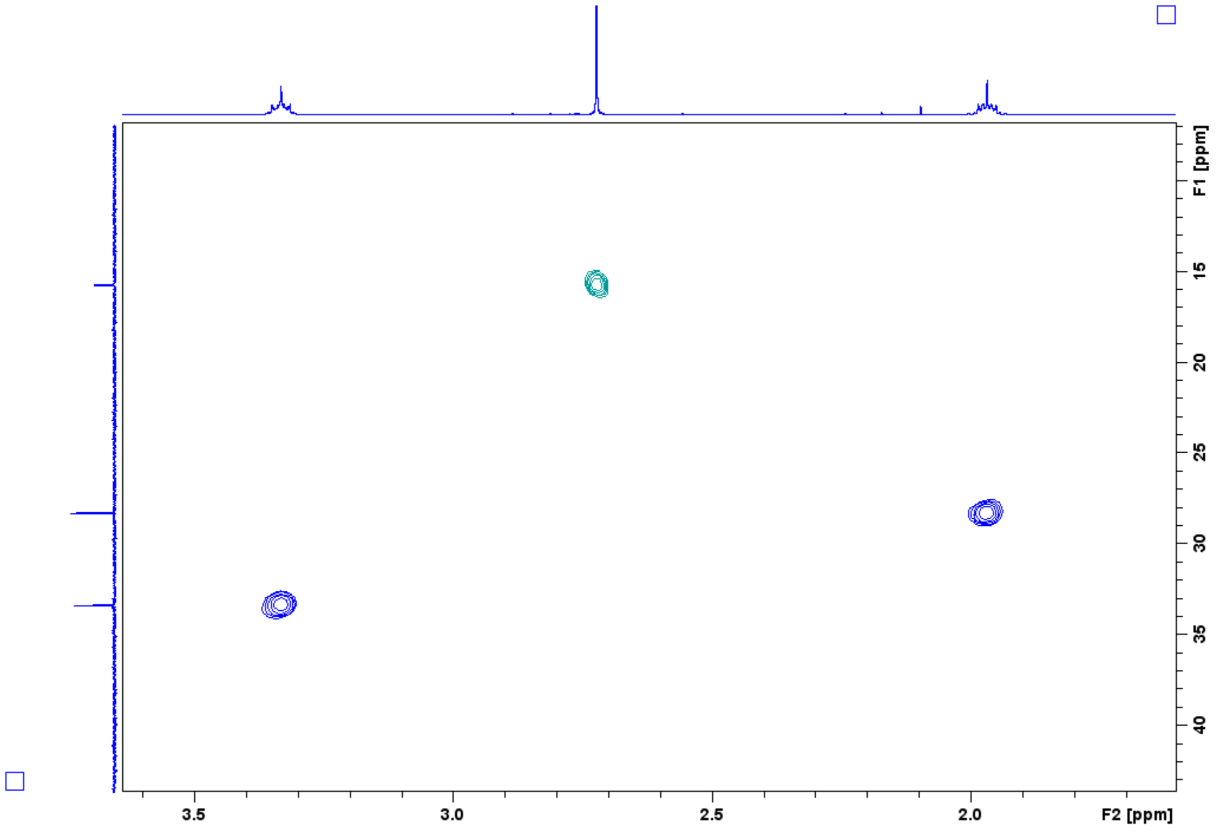

^{1}H-^{13}C HSQC spectrum of compound **9**.

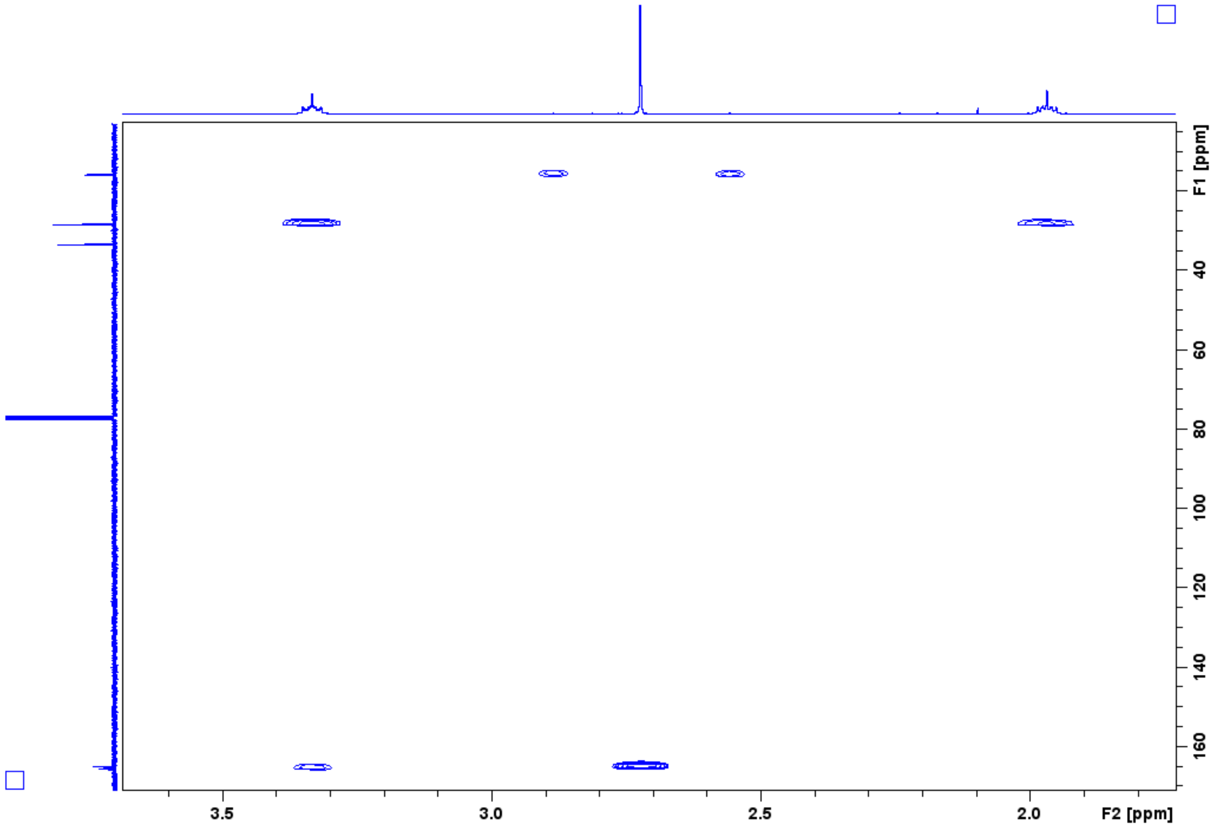

^{1}H-^{13}C HMBC spectrum of compound **9**.

NMR spectra of compound 10

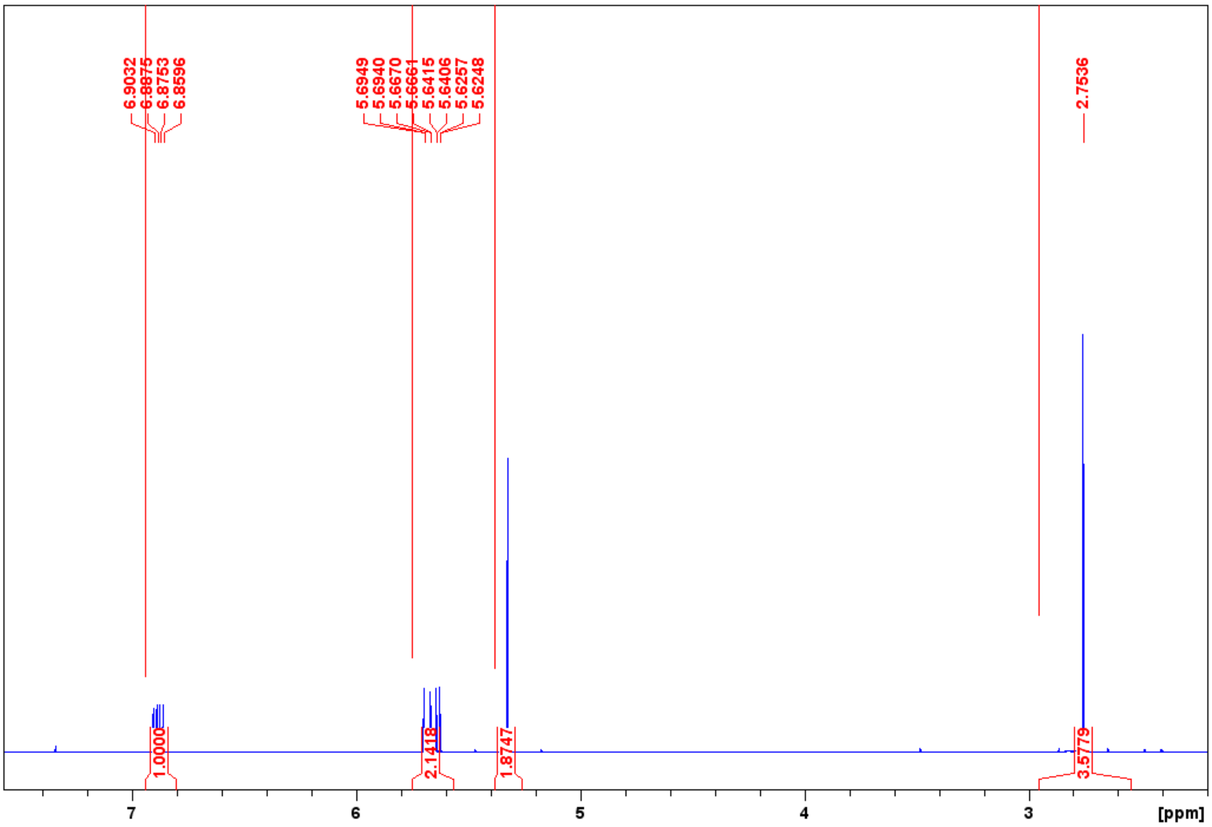

^{1}H NMR spectrum of compound **10**.

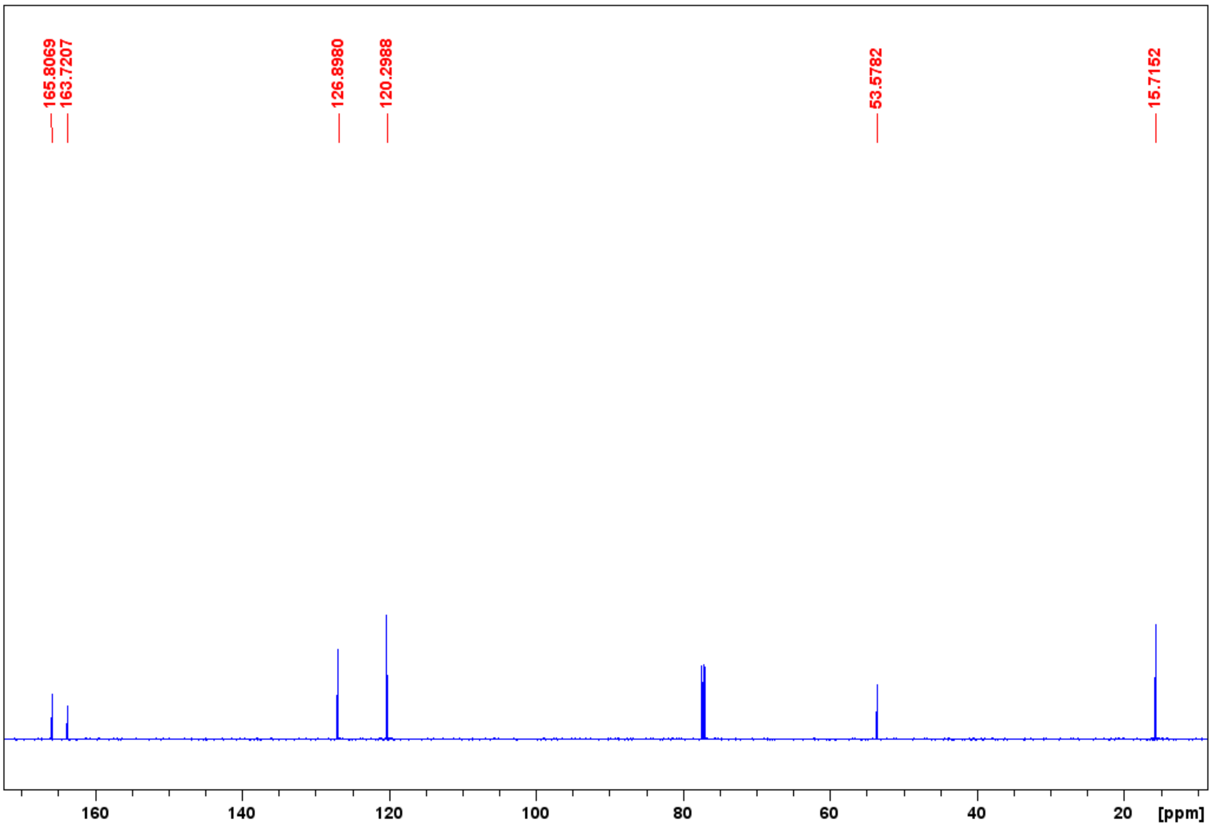

^{13}C NMR spectrum of compound **10**.

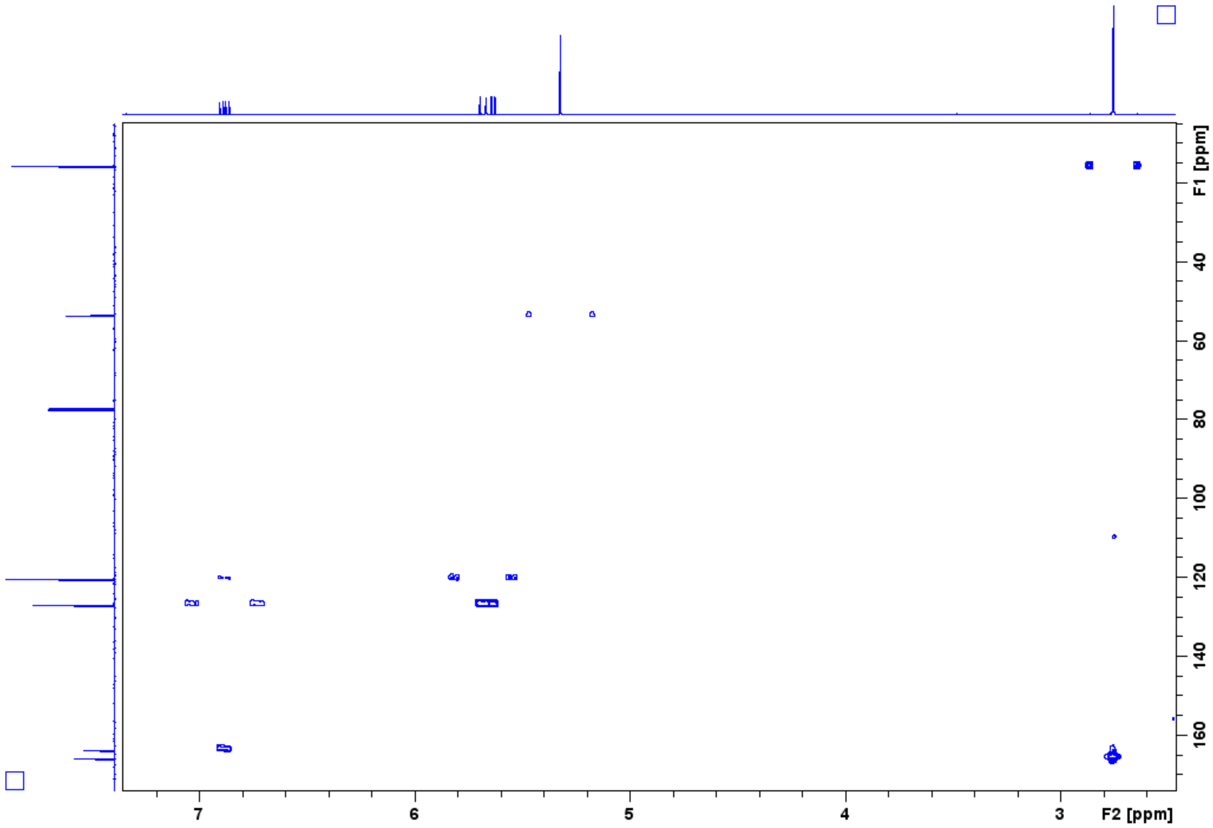

^{1}H-^{13}C HMBC spectrum of compound **10**.

NMR spectra of compound 2b

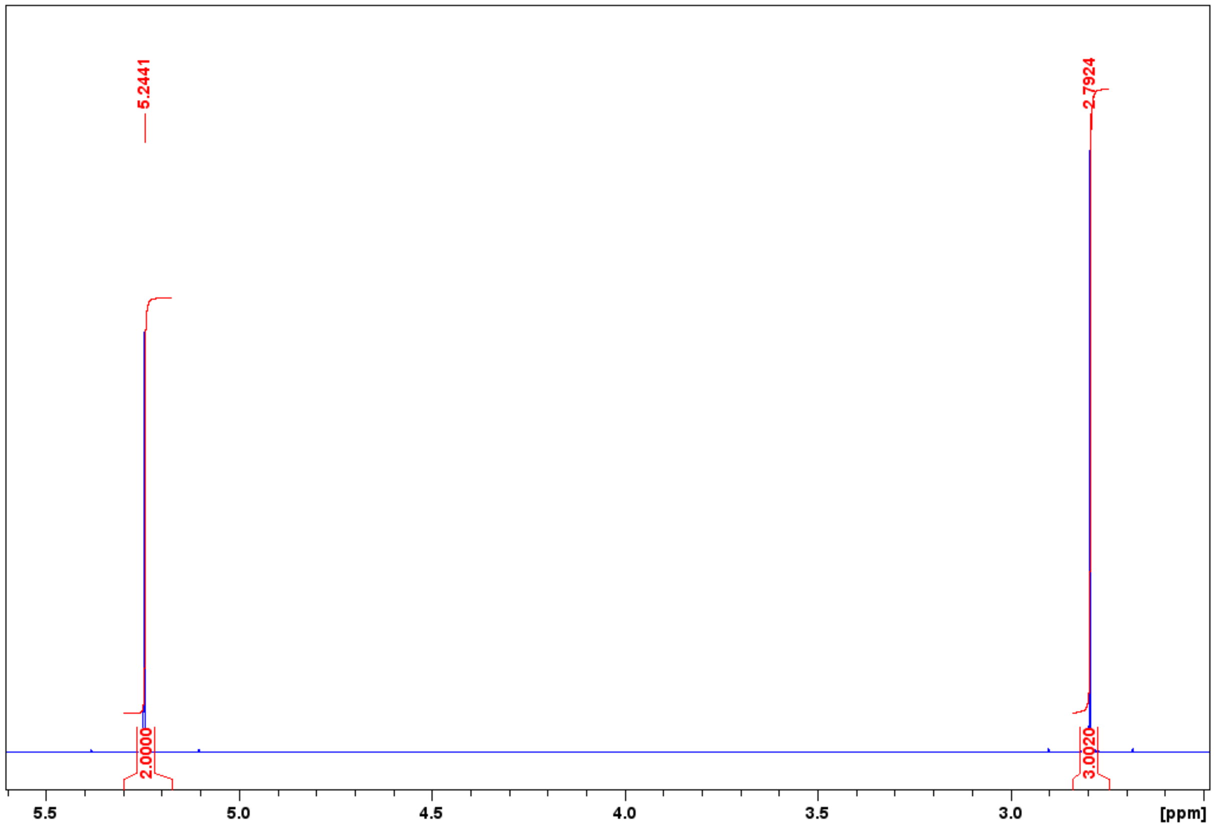

^{1}H NMR spectrum of compound **2b**.

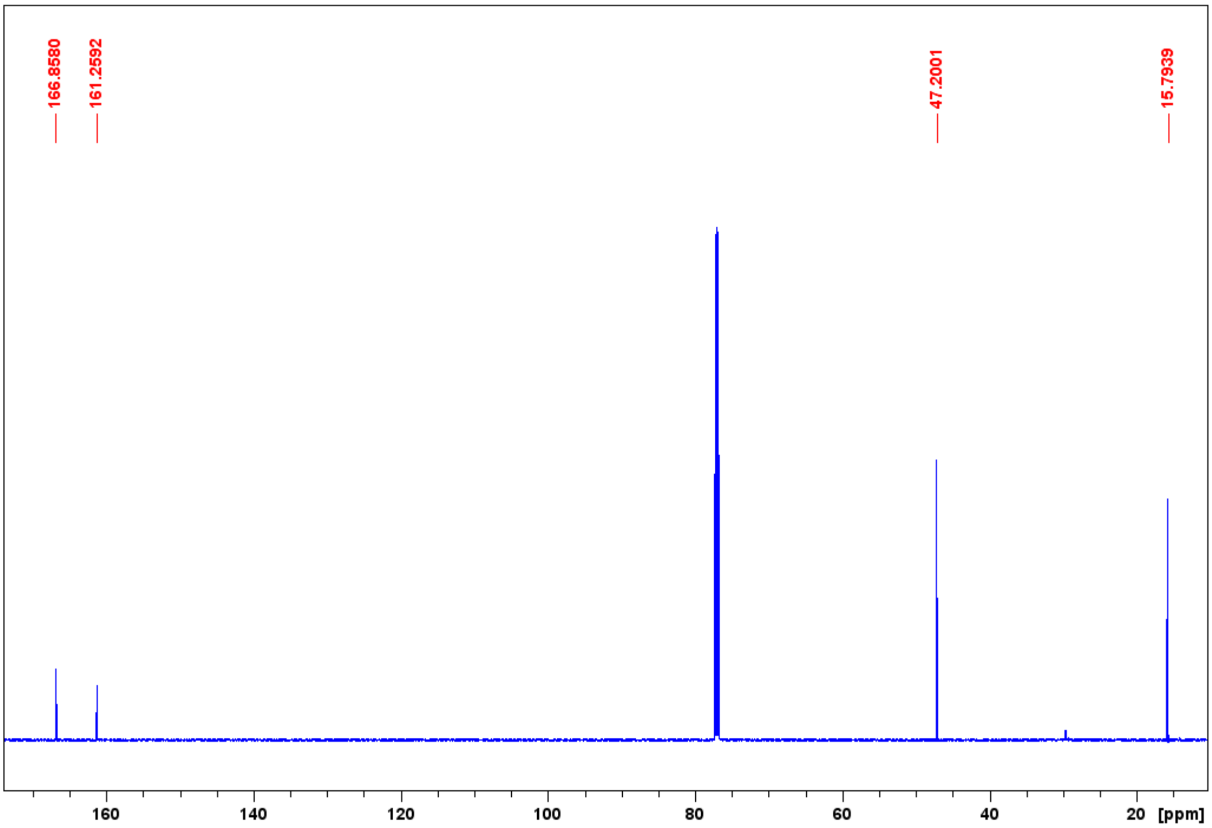

^{13}C NMR spectrum of compound **2b**.

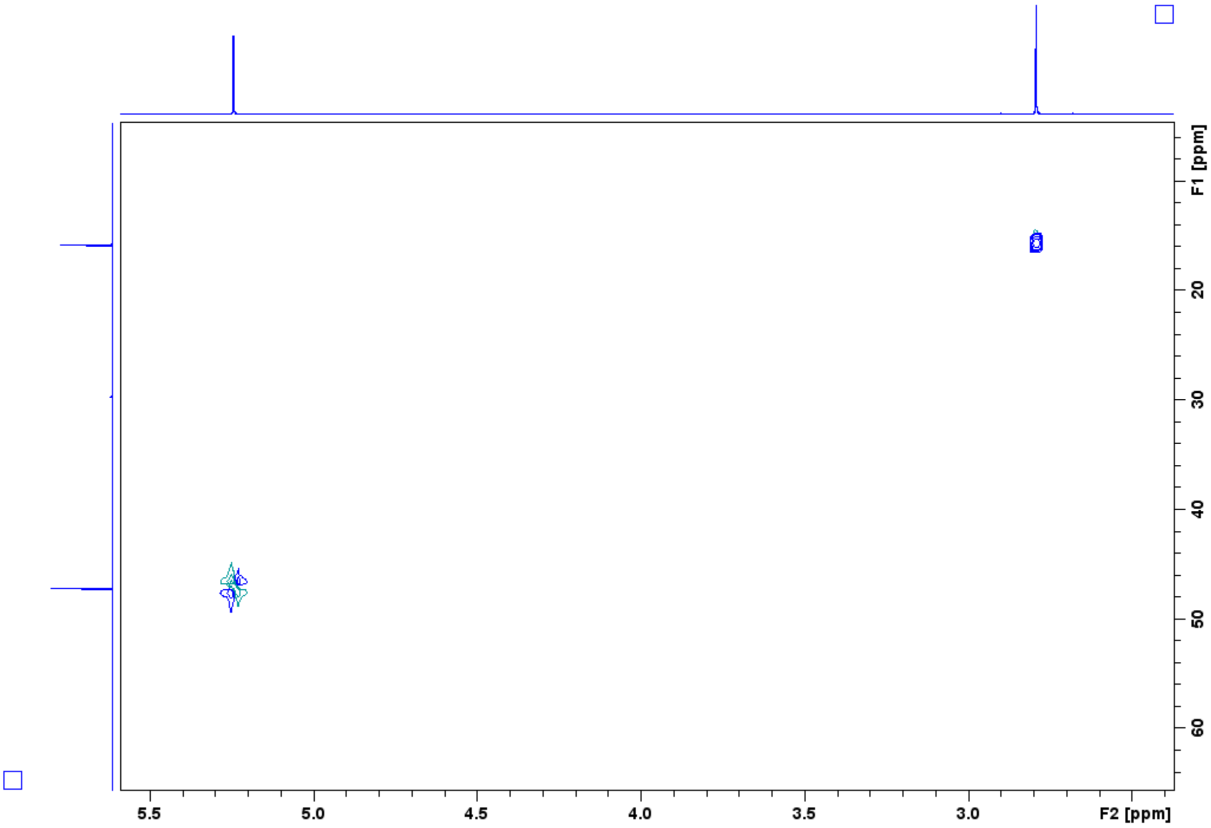

^{1}H-^{13}C HSQC spectrum of compound **2b**.

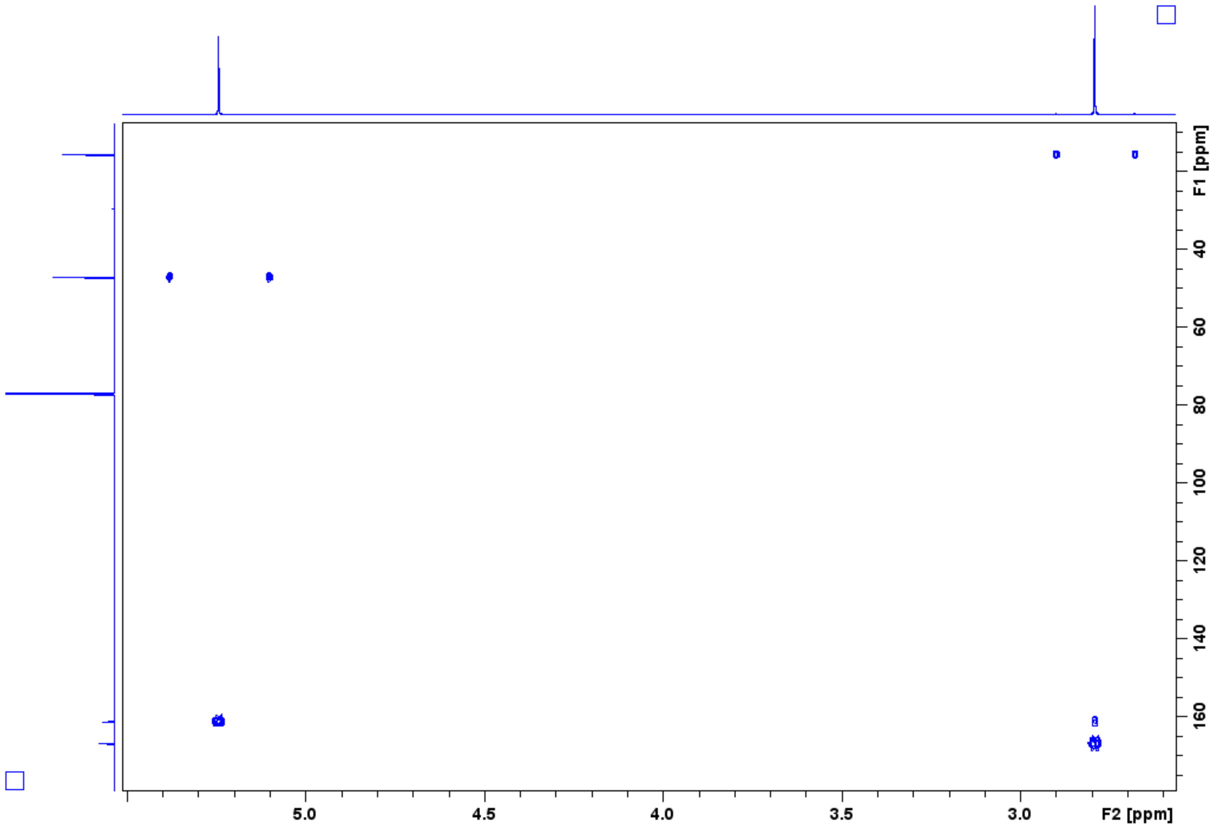

^1H-^{13}C HMBC spectrum of compound **2b**.

NMR spectra of compound 4b

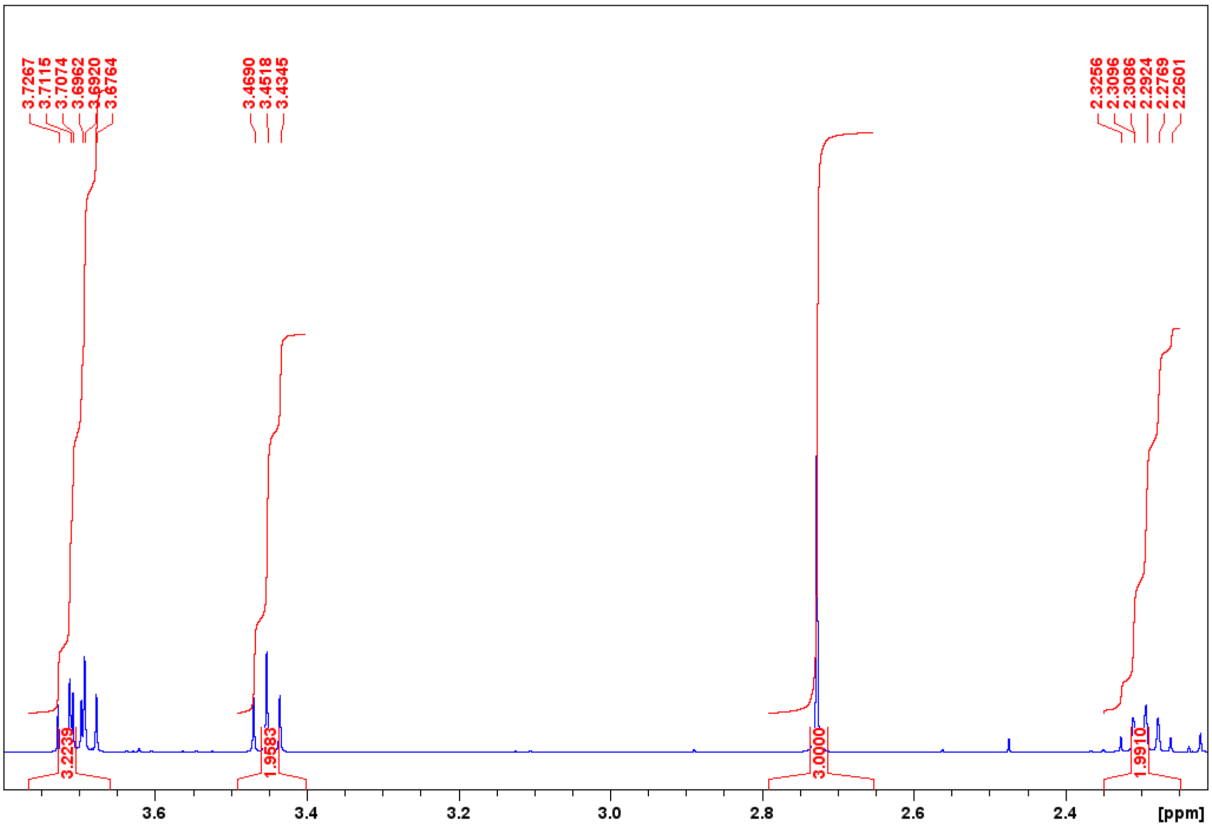

^1H NMR spectrum of compound **4b**.

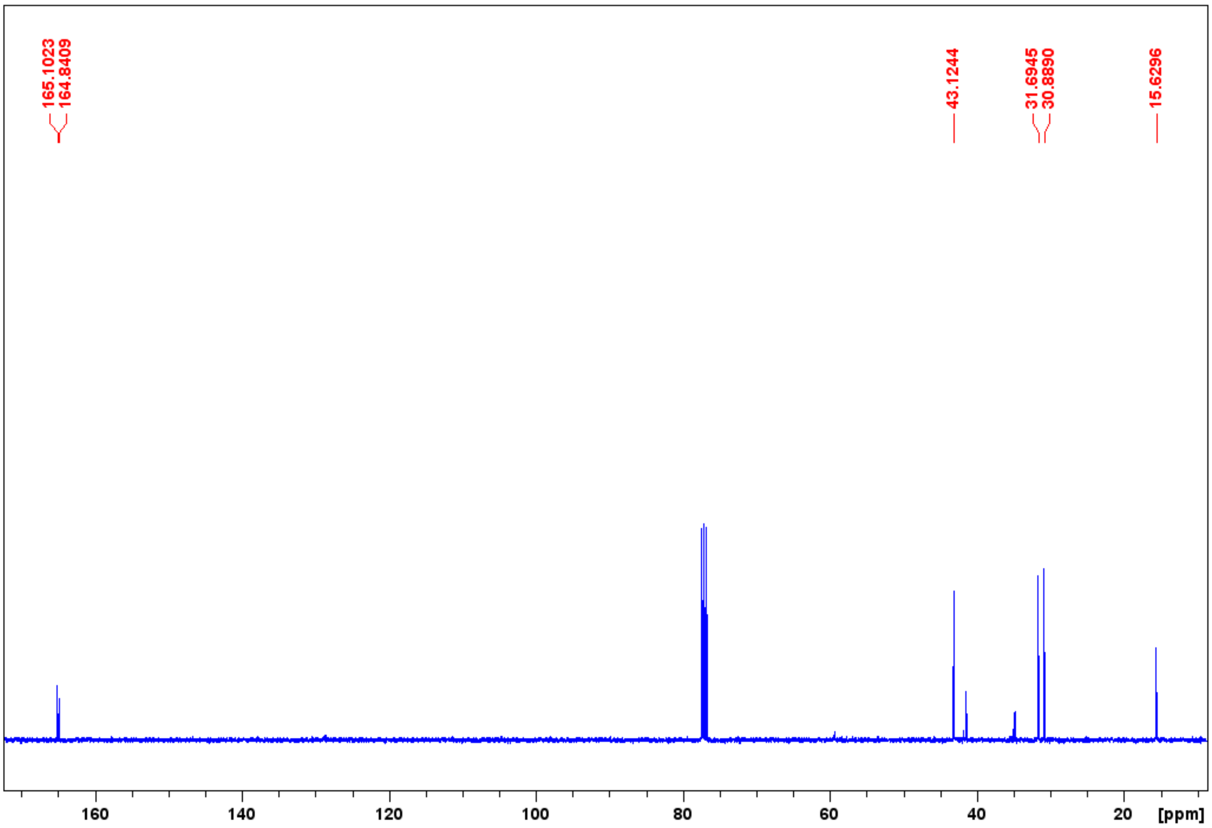

^{13}C NMR spectrum of compound **4b**.

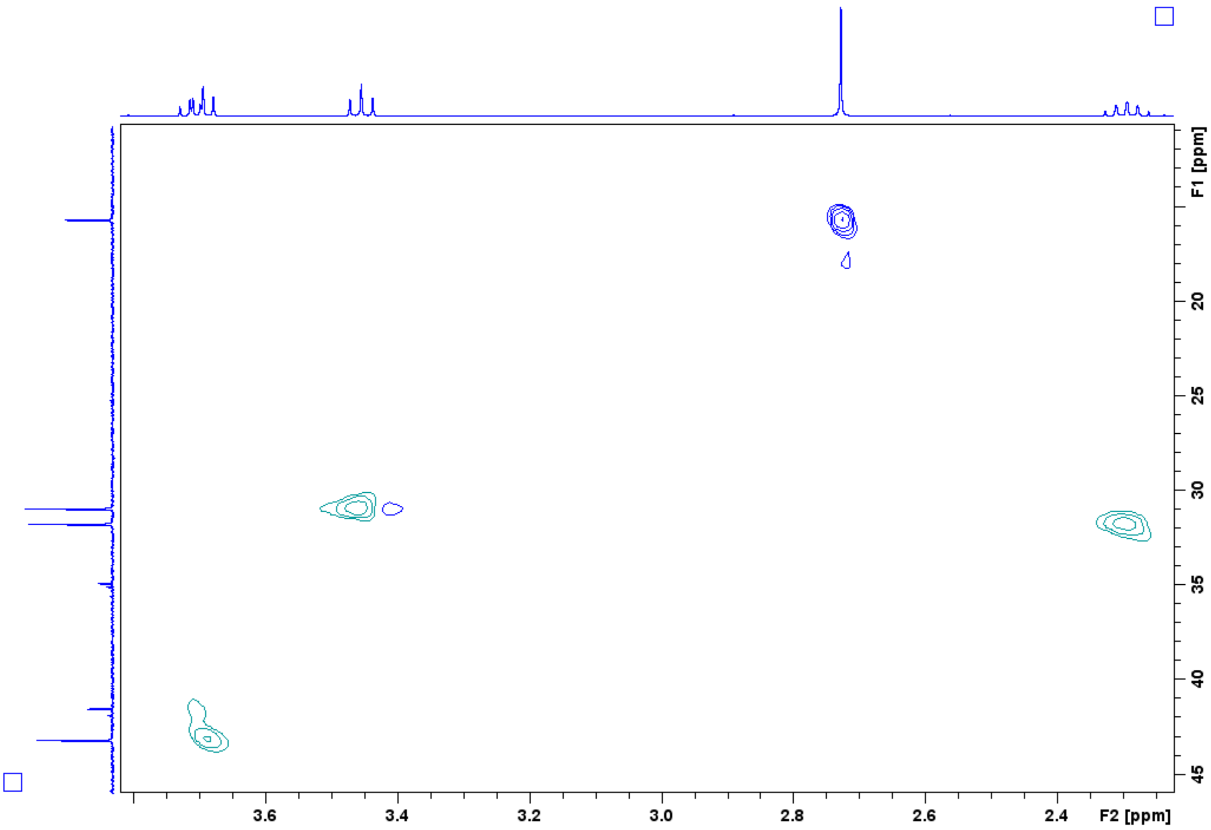

^{1}H-^{13}C HSQC spectrum of compound **4b**.

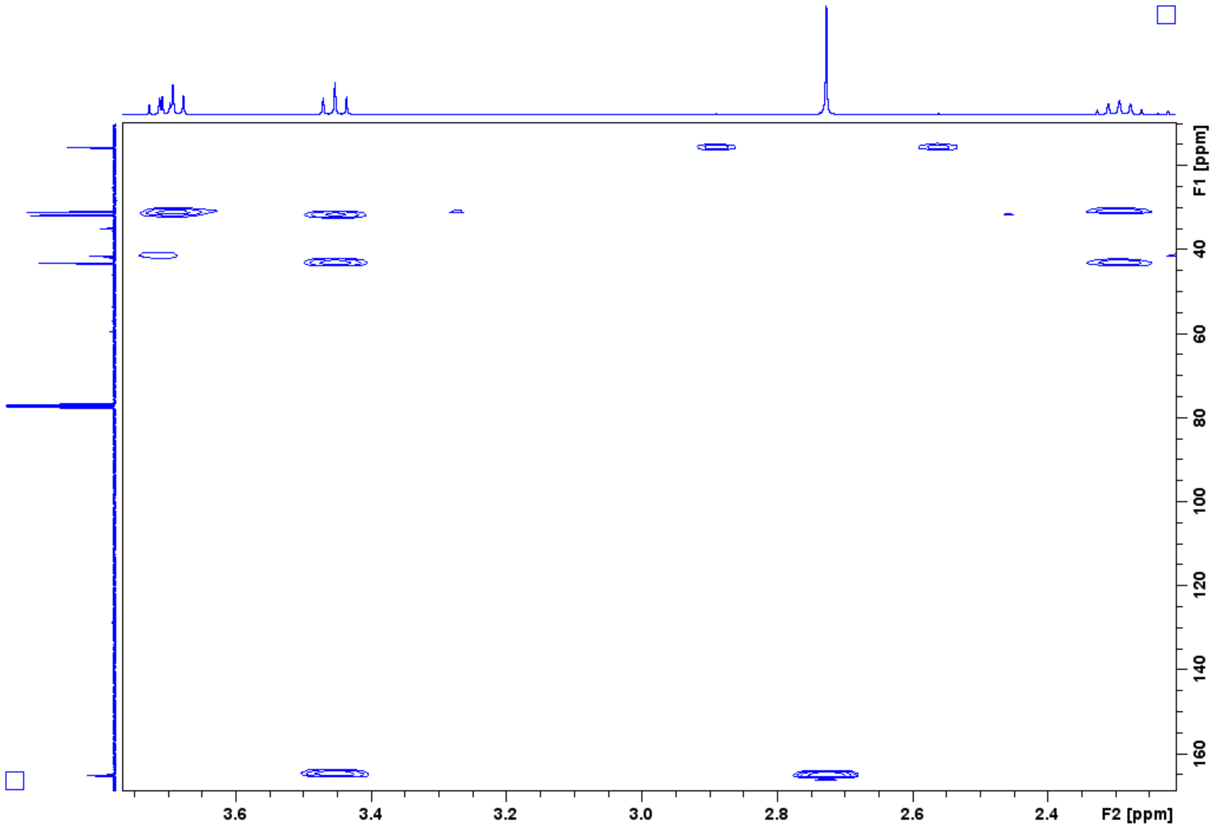

^{1}H-^{13}C HMBC spectrum of compound **4b**.

NMR spectra of compound 5b

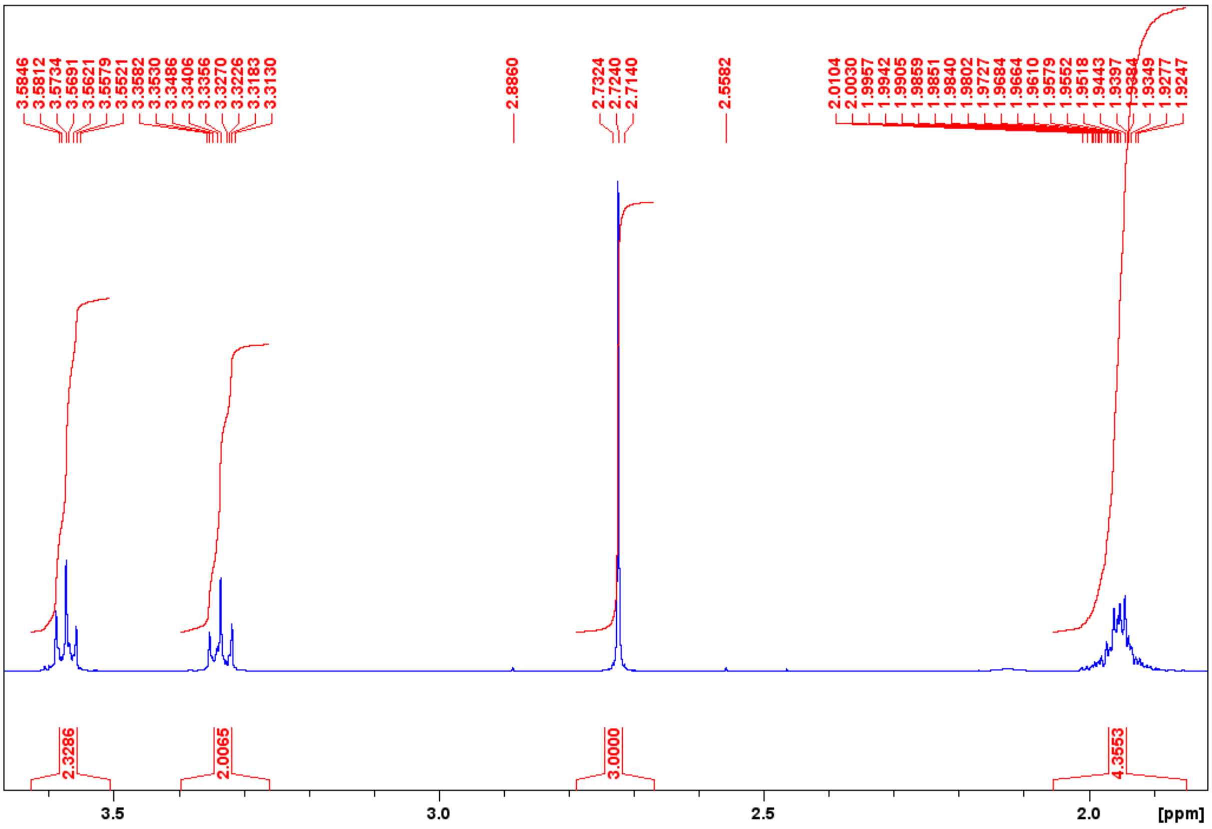

^{1}H NMR spectrum of compound **5b**.

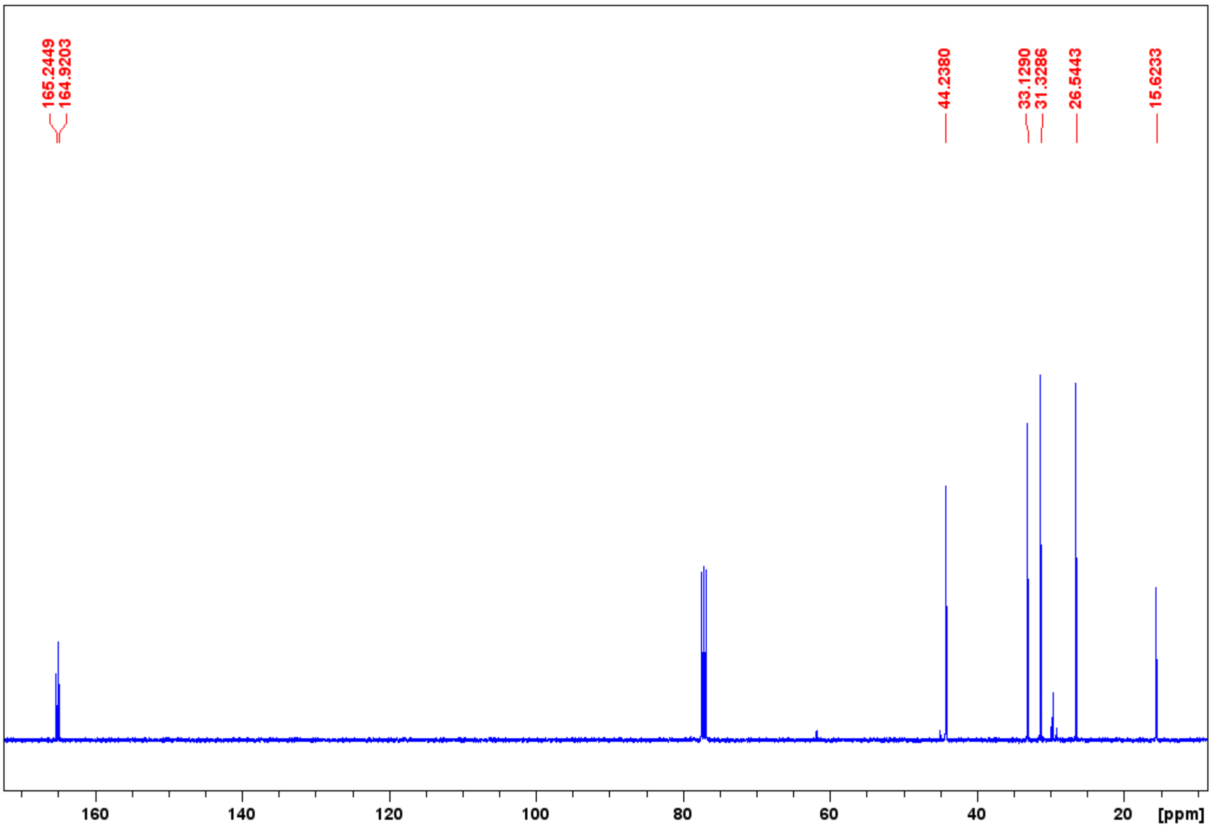

^{13}C NMR spectrum of compound **5b**.

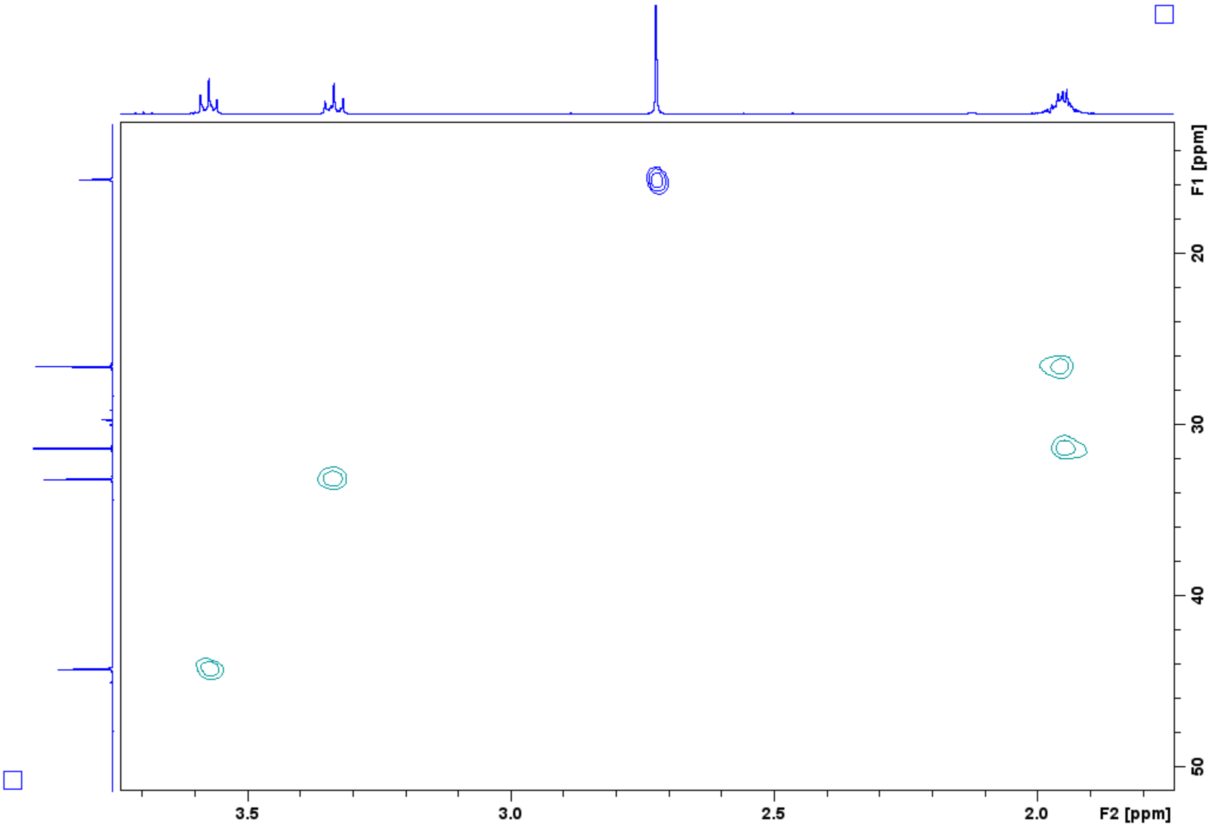

^{1}H-^{13}C HSQC spectrum of compound **5b**.

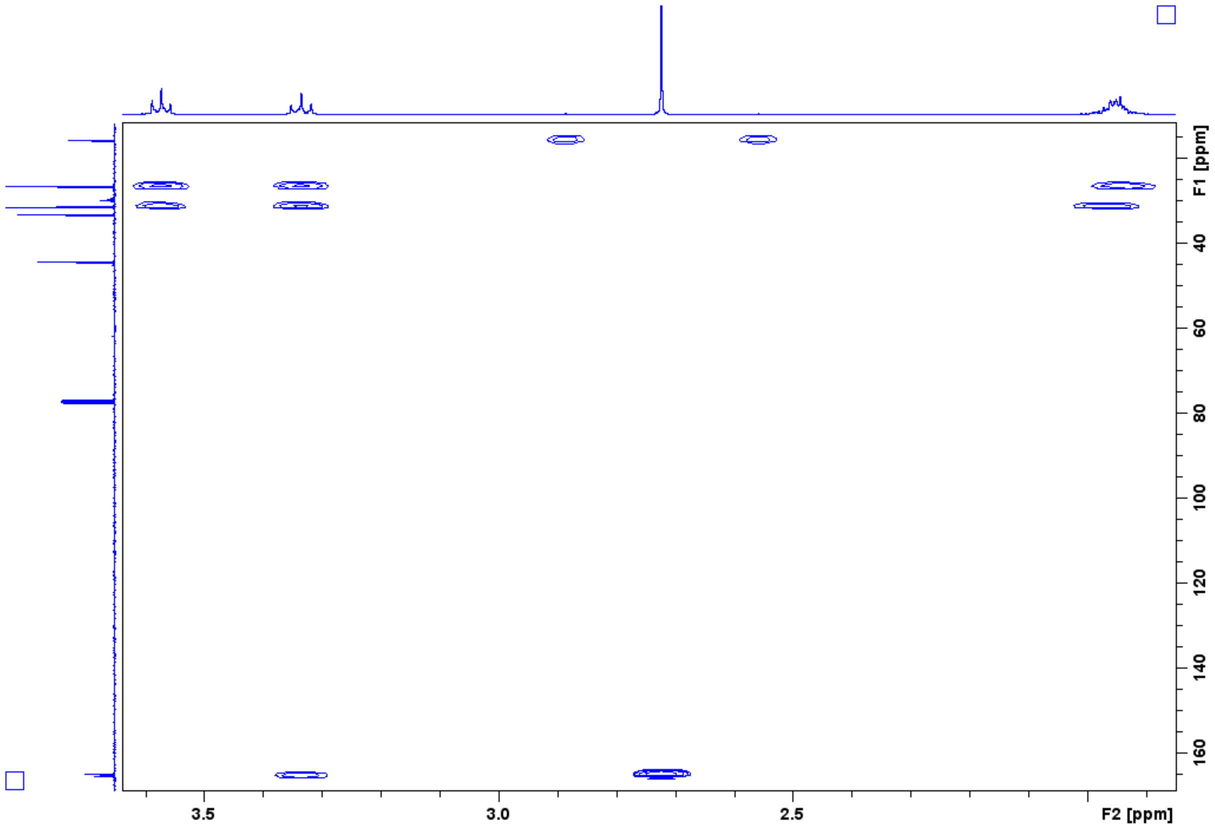

^{1}H-^{13}C HMBC spectrum of compound **5b**.

IR (ATR) spectra of the products

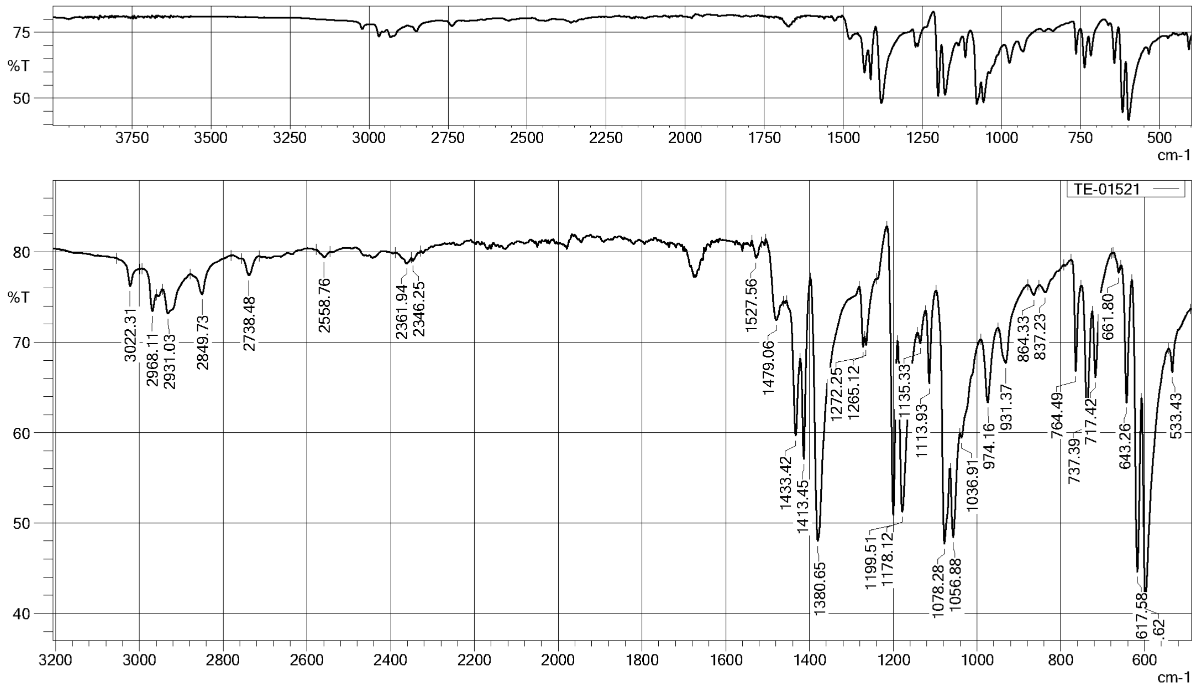

IR (ATR) spectrum of bromide **3a**.

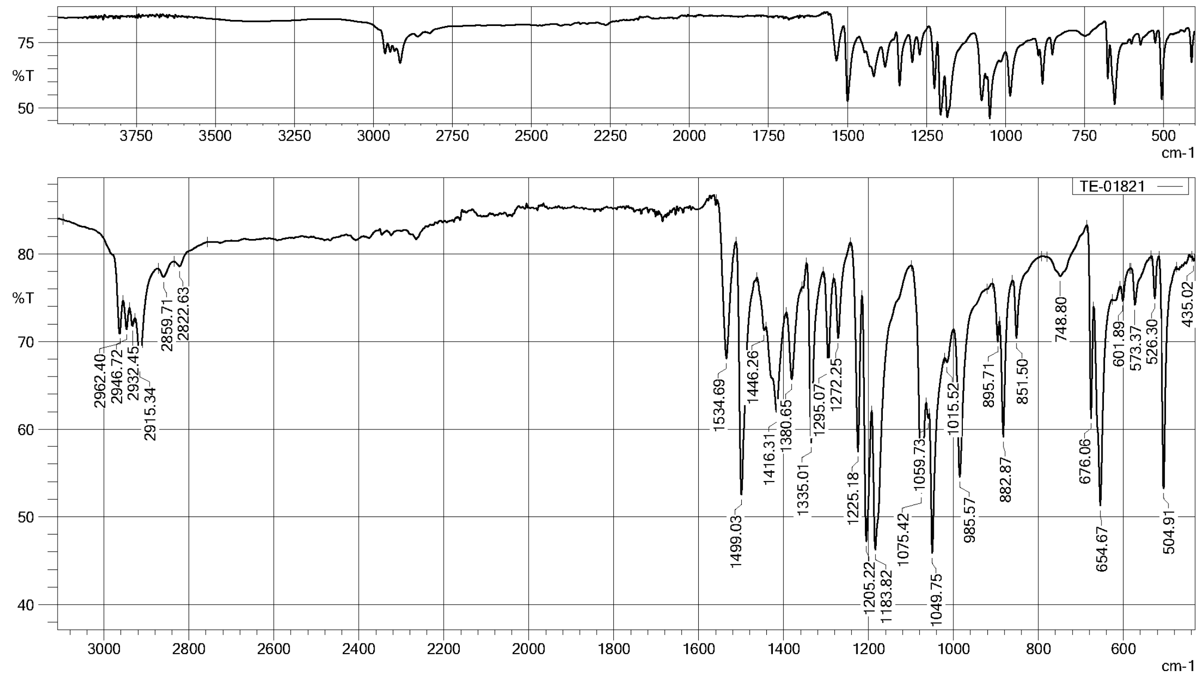

IR (ATR) spectrum of bromide **4a**.

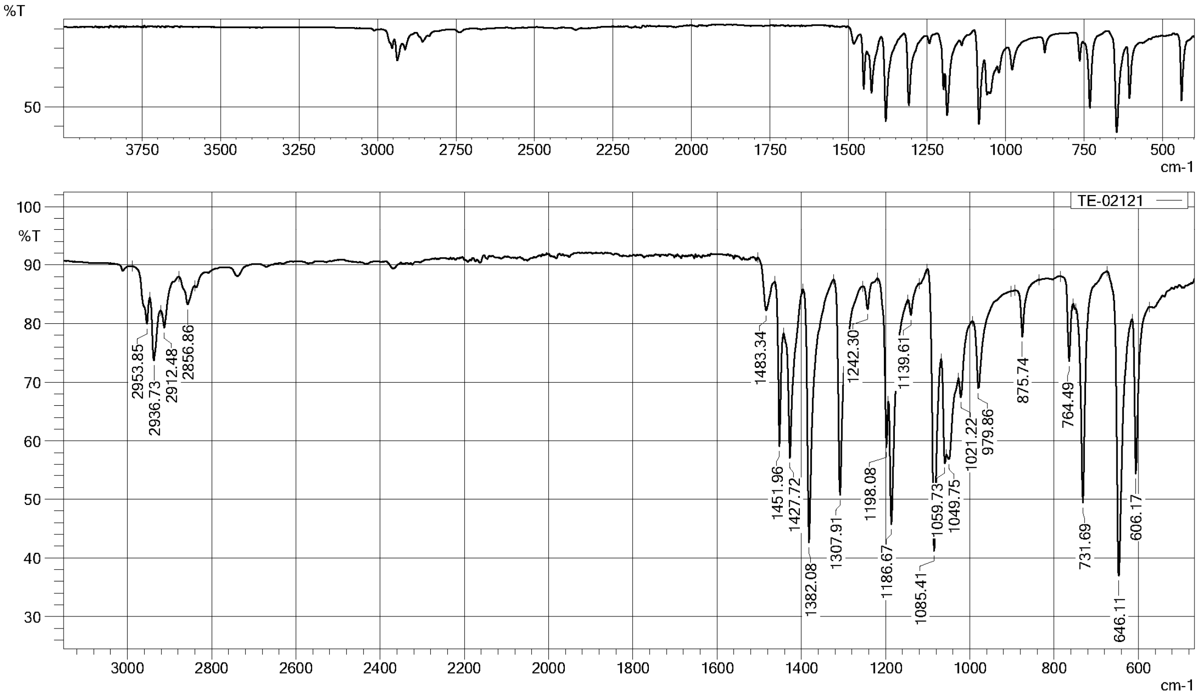

IR (ATR) spectrum of bromide **5a**.

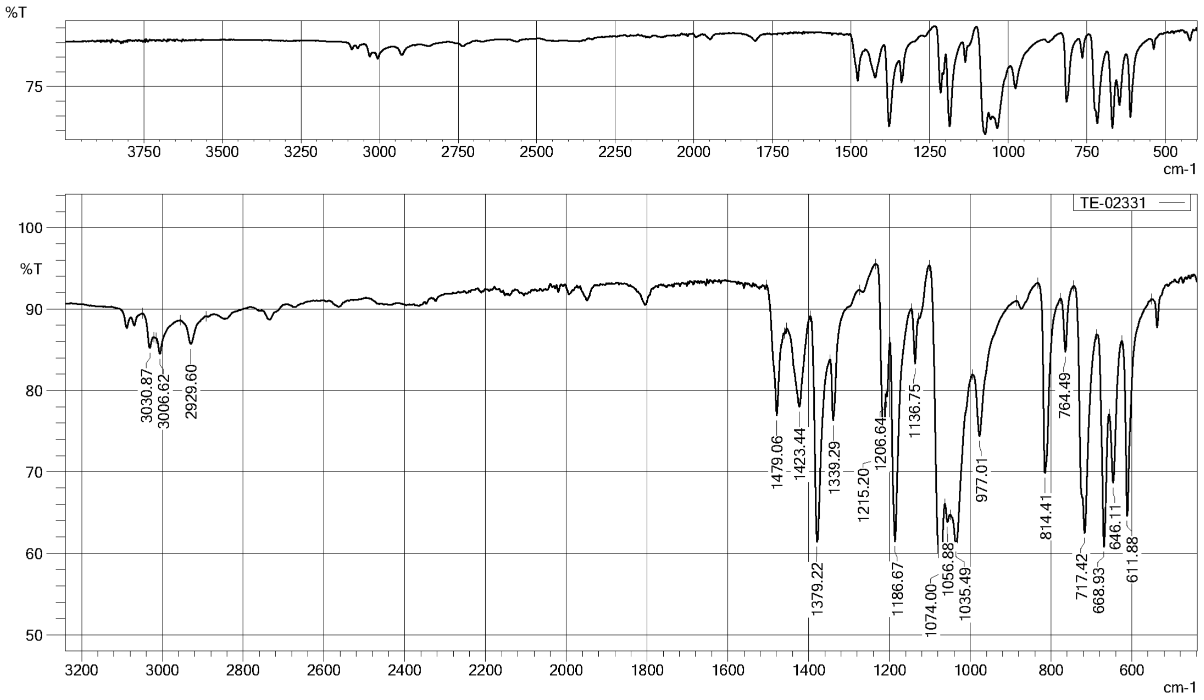

IR (ATR) spectrum of the ligand **6**.

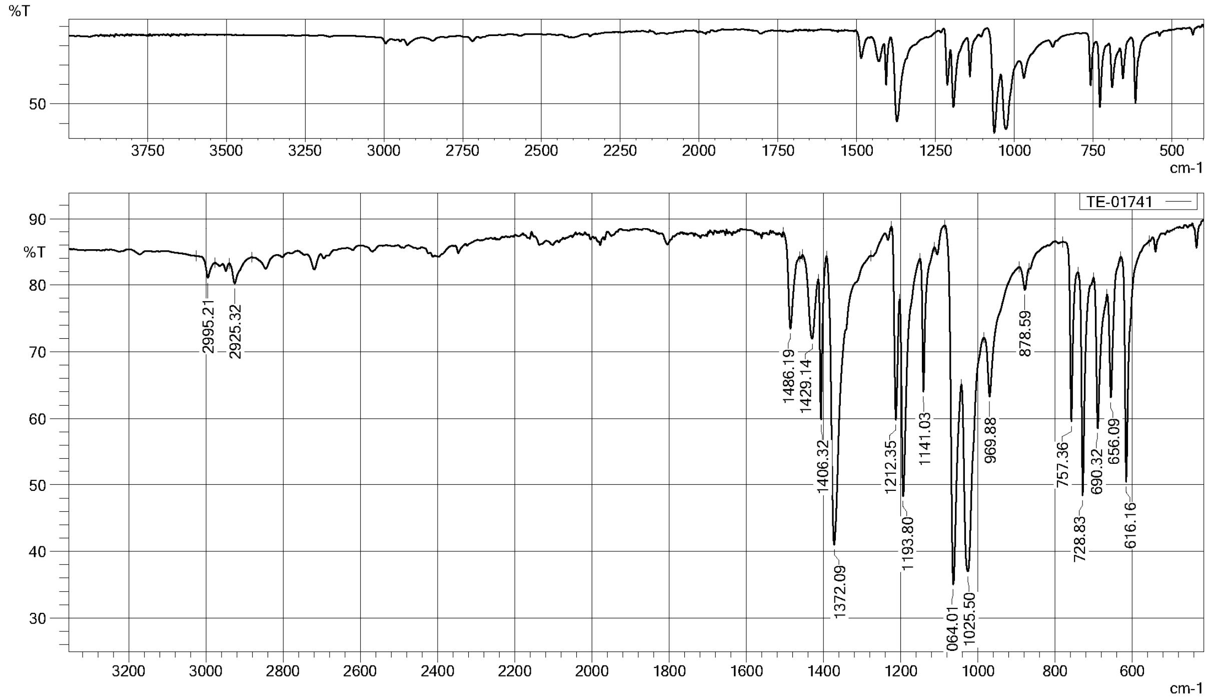

IR (ATR) spectrum of the ligand **7**.

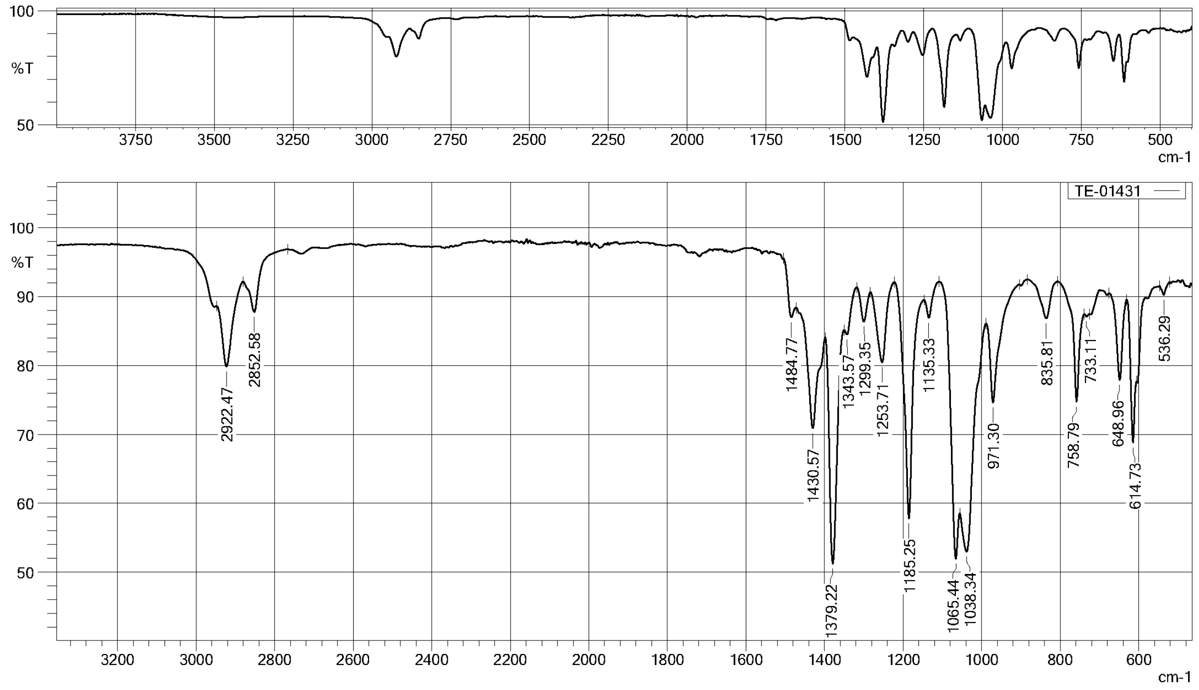

IR (ATR) spectrum of the ligand **8**.

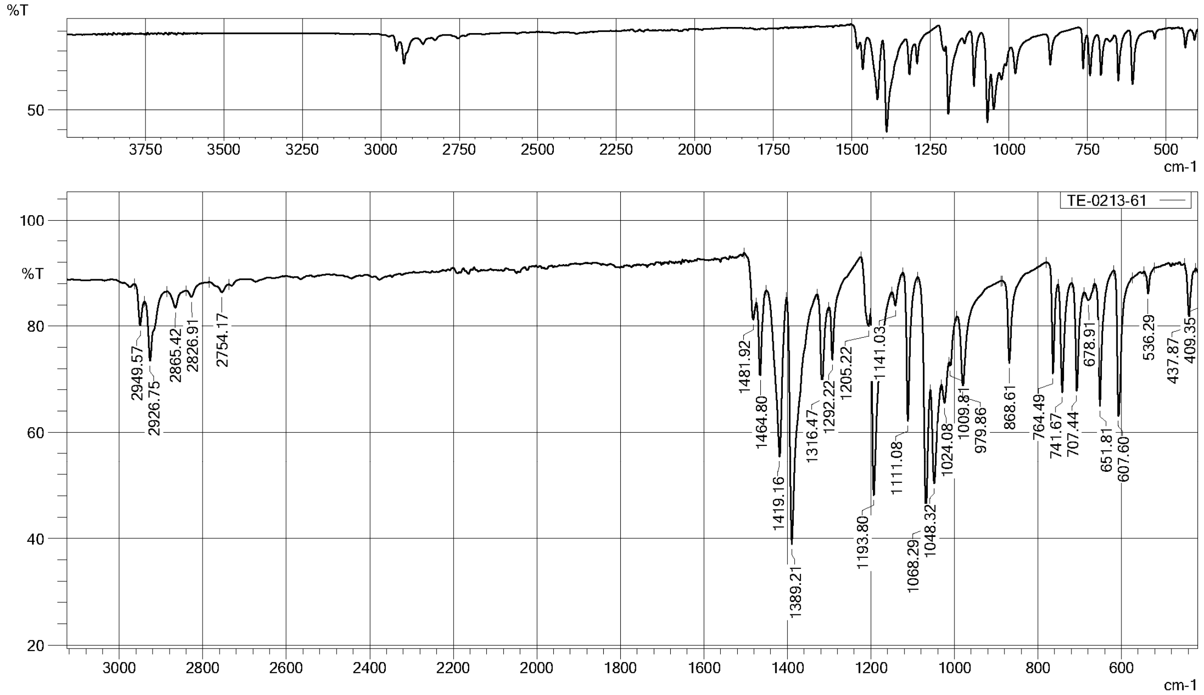

IR (ATR) spectrum of ligand **9**.

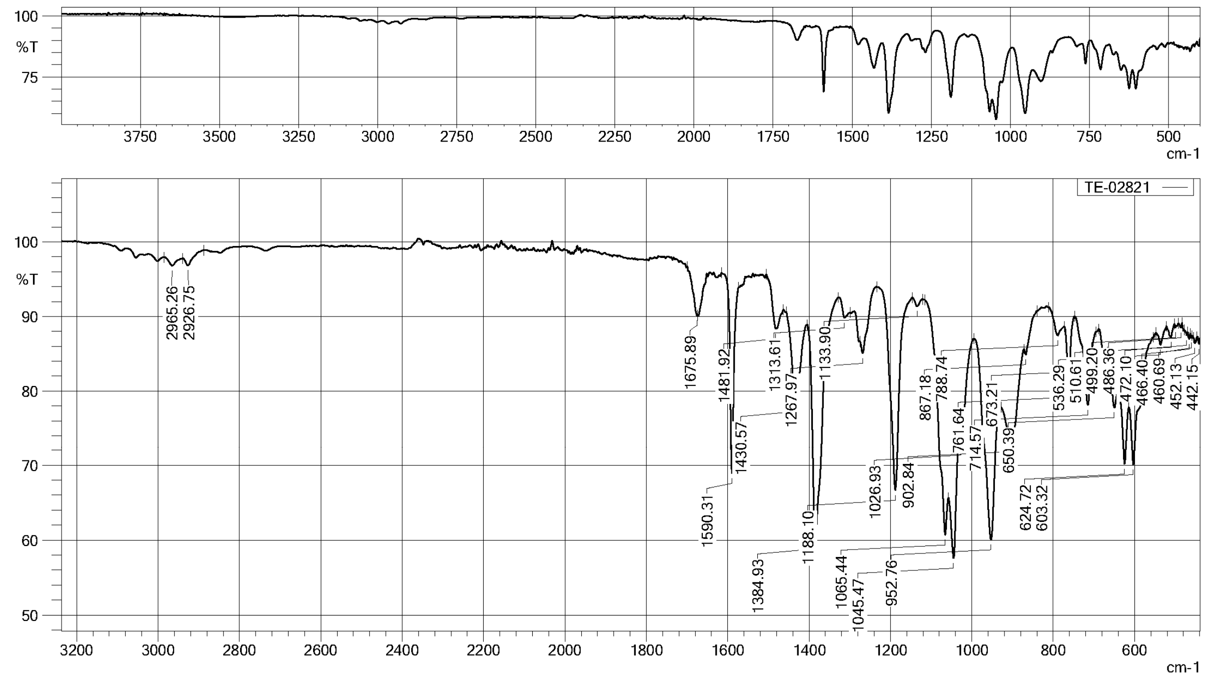

IR (ATR) spectrum of olefin **10**.

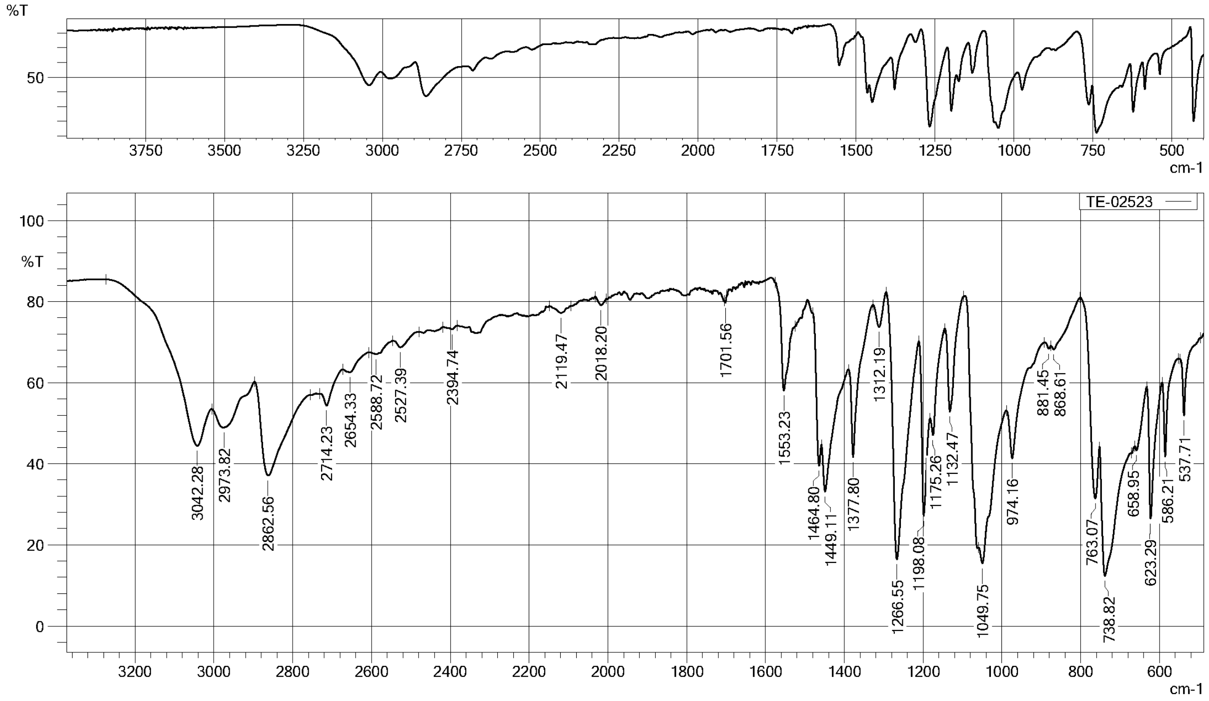

IR (ATR) spectrum of chloride **2b**.

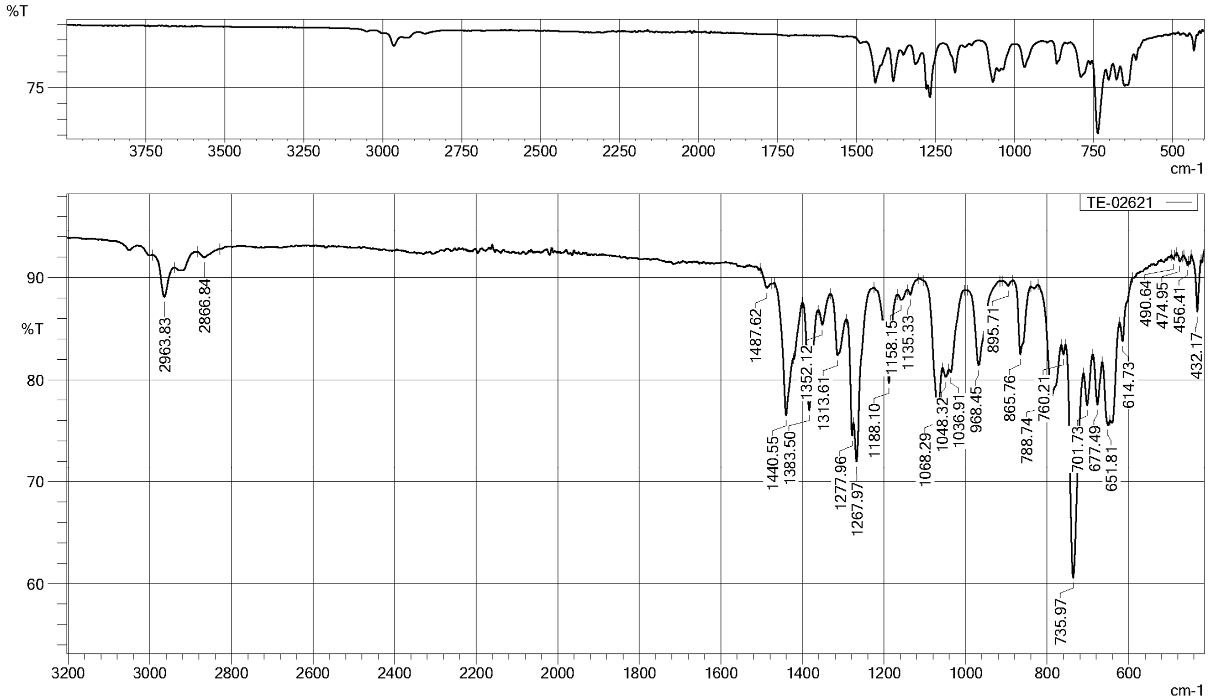

IR (ATR) spectrum of chloride **4b**.

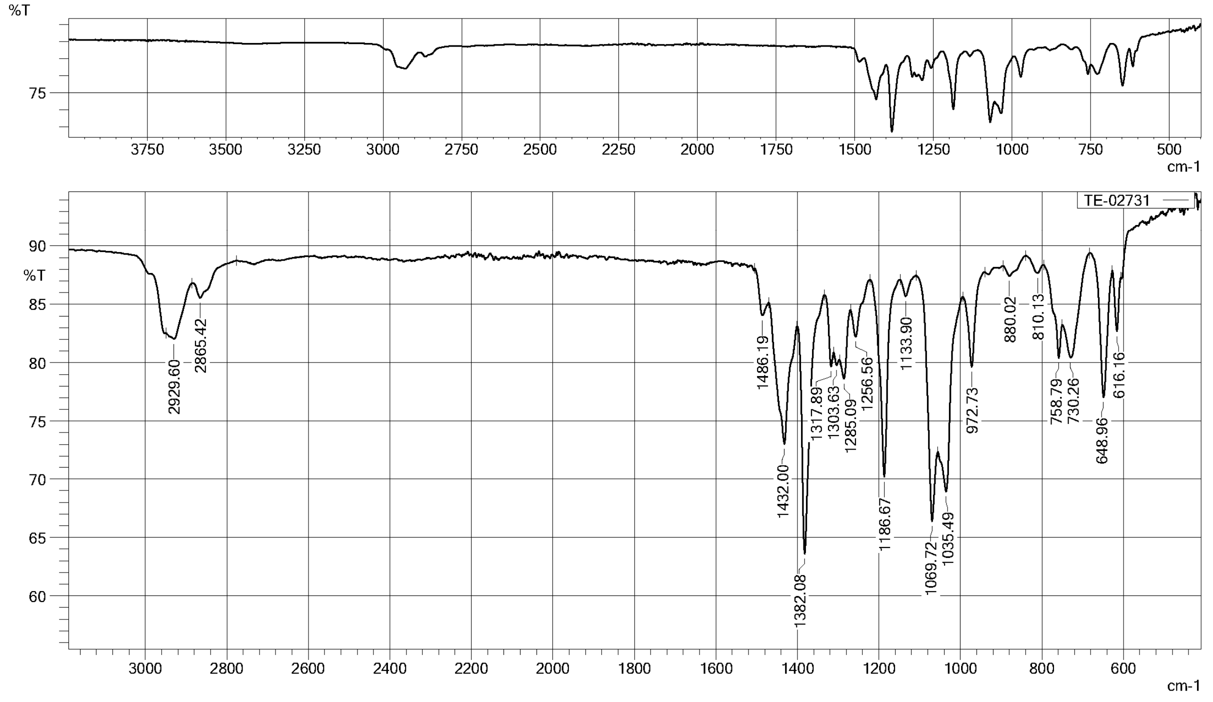

IR (ATR) spectrum of chloride **5b**.

HR-MS (HESI$^+$) spectra of the products

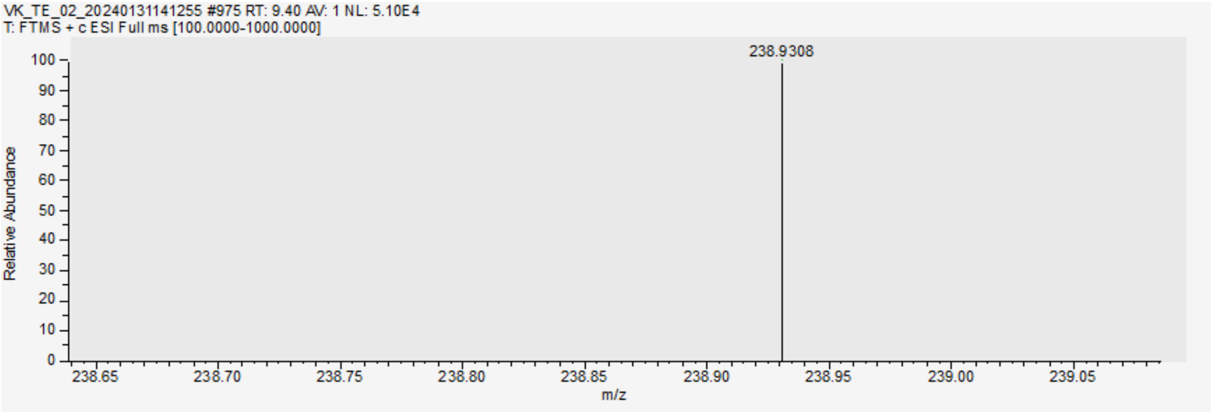

HR-MS (HESI$^+$) spectrum of bromide **3a**.

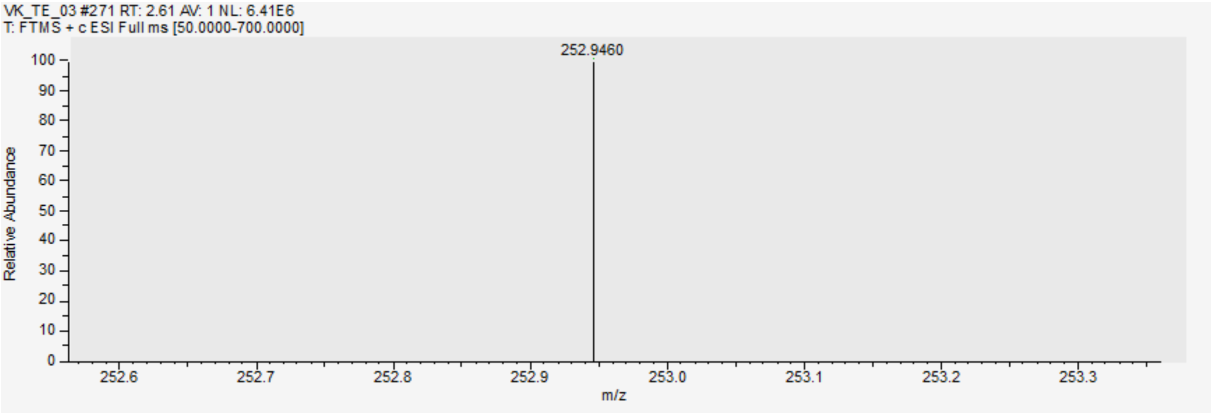

HR-MS (HESI$^+$) spectrum of bromide **4a**.

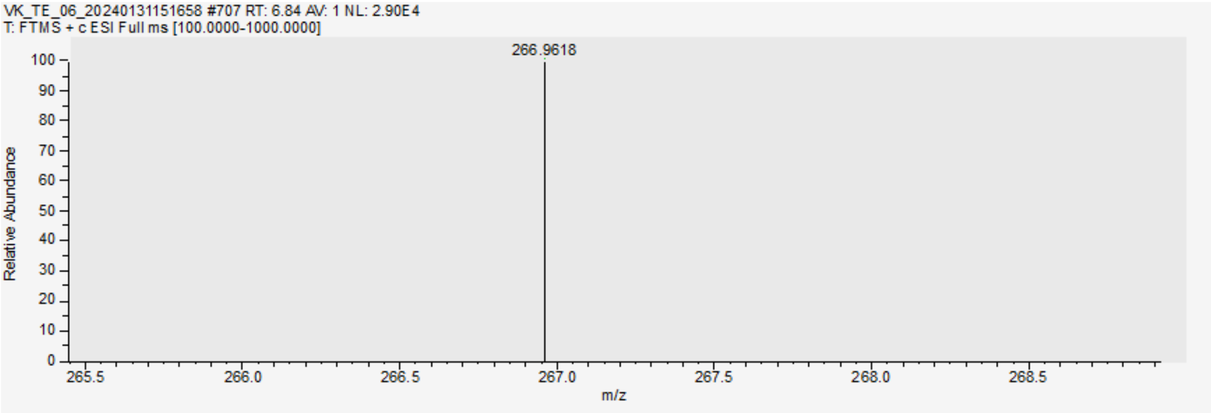

HR-MS (HESI$^+$) spectrum of bromide **5a**.

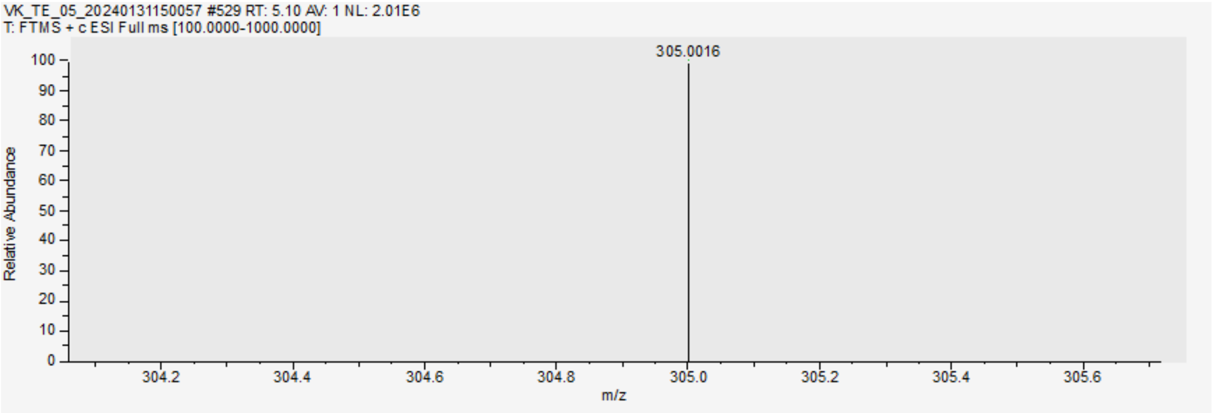

HR-MS (HESI$^+$) spectrum of the ligand **8**.

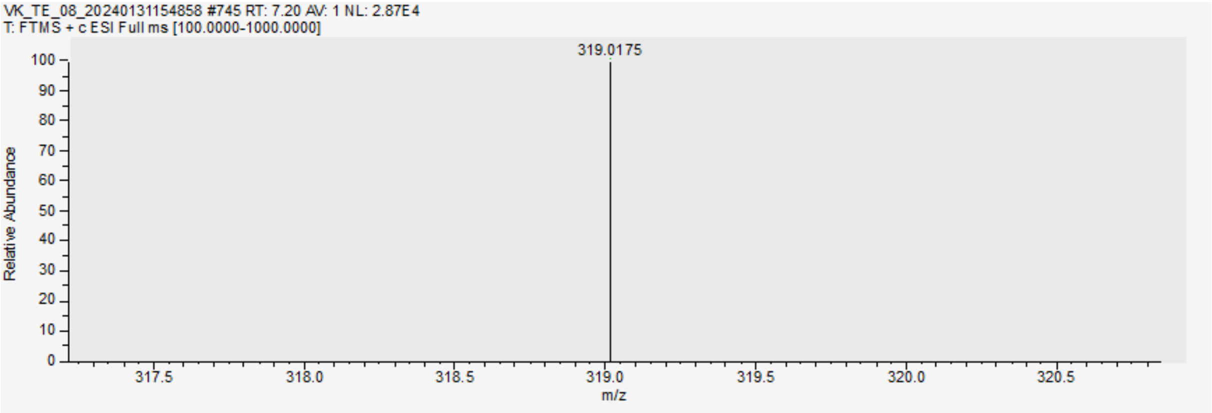

HR-MS (HESI$^+$) spectrum of the ligand **9**.

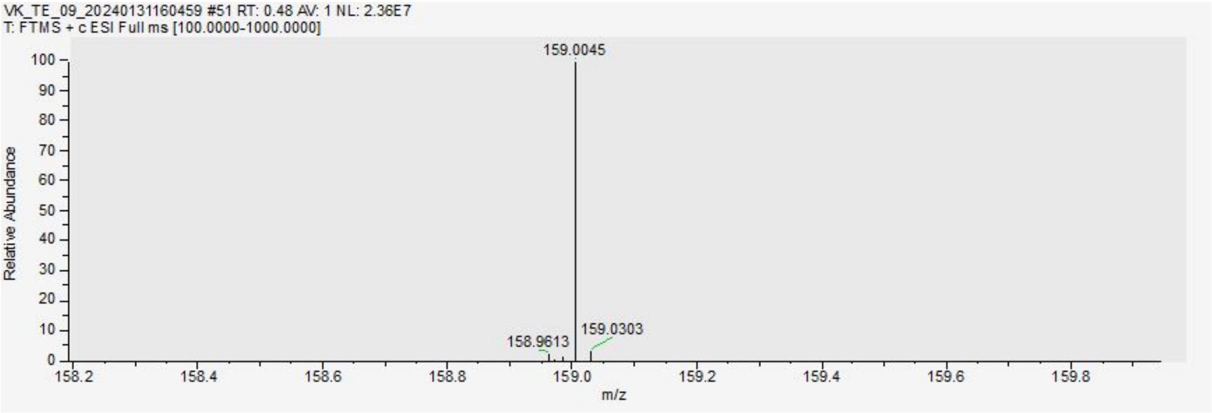

HR-MS (HESI$^+$) spectrum of olefin **10**.

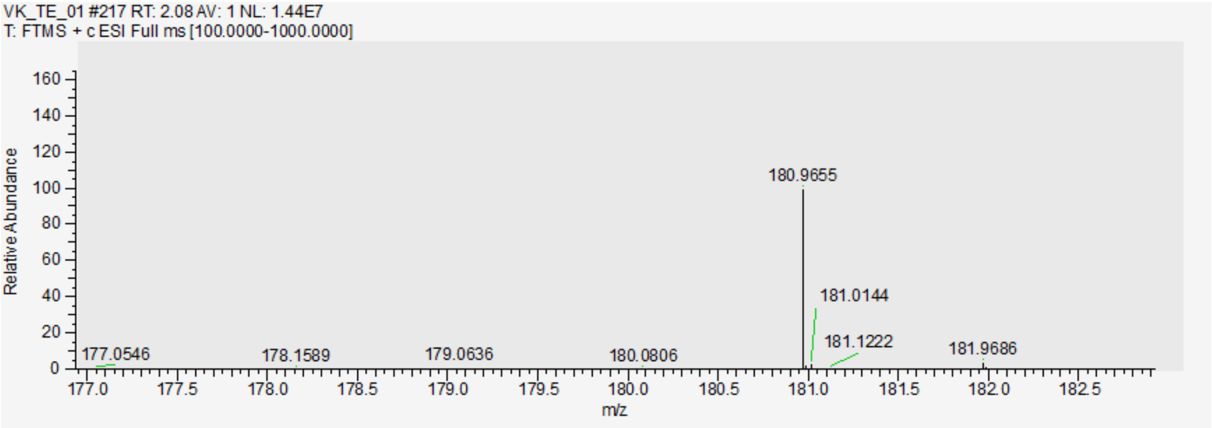

HR-MS (HESI$^+$) spectrum of chloride **2b**.

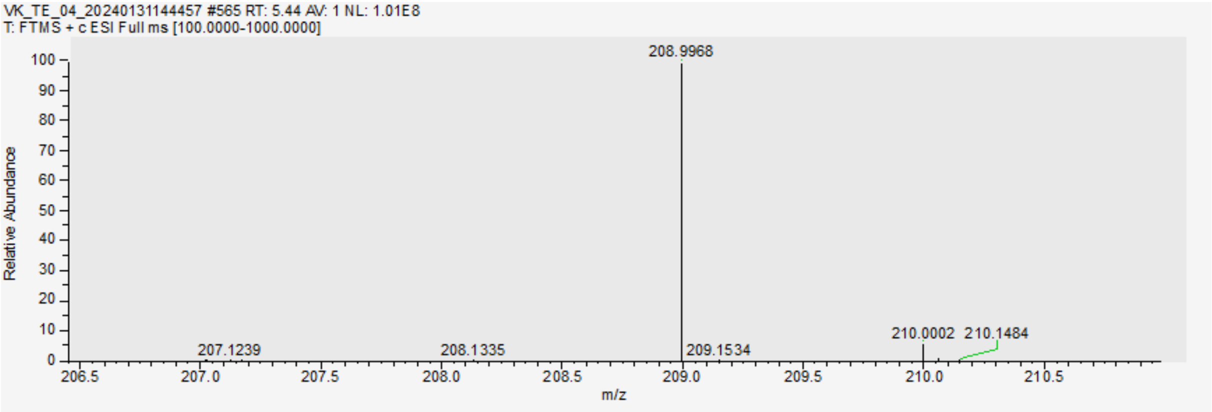

HR-MS (HESI$^+$) spectrum of chloride **4b**.

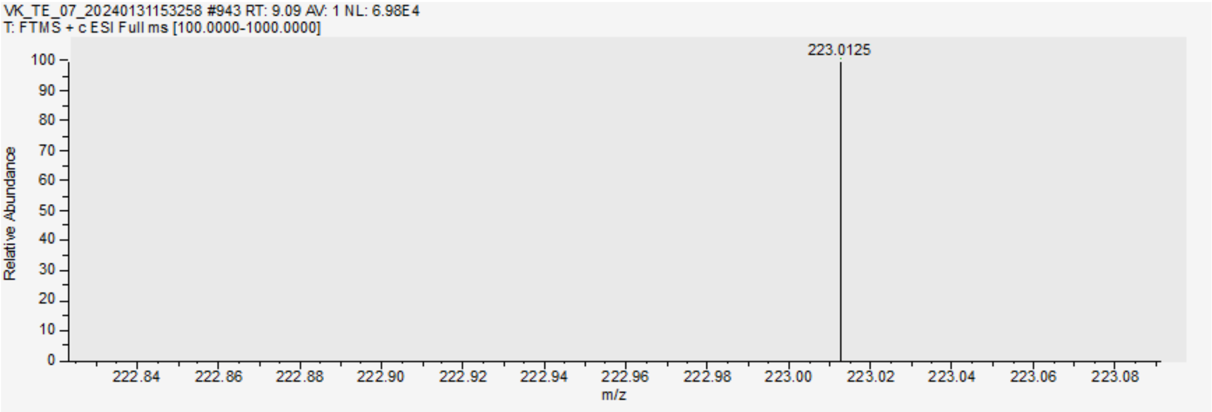

HR-MS (HESI$^+$) spectrum of chloride **5b**.
